# Induction of Chirality in MXene Nanosheets and Derived Quantum Dots: Chiral Mixed‐Low‐Dimensional Ti_3_C_2_T_x_ Biomaterials as Potential Agricultural Biostimulants for Enhancing Plant Tolerance to Different Abiotic Stresses

**DOI:** 10.1002/smll.202500654

**Published:** 2025-04-03

**Authors:** Alireza Rafieerad, Soofia Khanahmadi, Akif Rahman, Hossein Shahali, Maik Böhmer, Ahmad Amiri

**Affiliations:** ^1^ Institute for Molecular Biosciences Johann Wolfgang Goethe Universität 60438 Frankfurt am Main Germany; ^2^ Institute for Biology and Biotechnology of Plants University of Münster Schlossplatz 8 48143 Münster Germany; ^3^ Regenerative Medicine Program Institute of Cardiovascular Sciences St. Boniface Hospital Research Centre Department of Physiology and Pathophysiology Rady Faculty of Health Sciences University of Manitoba Winnipeg Manitoba R2H 2A6 Canada; ^4^ Department of Mechanical Engineering The University of Tulsa Tulsa OK 74104 USA; ^5^ Russell School of Chemical Engineering University of Tulsa Tulsa OK 74104 USA

**Keywords:** DFT calculations, enhanced plant/seedling abiotic stress tolerance, improved colloidal dispersibility, induced‐stomatal closure and ROS production mechanisms, optically asymmetric Ti_3_C_2_T_x_, right‐/left‐handed chiral MXenes, stable mixed‐low‐dimensional heterostructures

## Abstract

This work presents two advancements in the engineering design and bio‐applications of emerging MXene nanosheets and derived quantum dots. First, a facile, versatile, and universal strategy is showcased for inducing the right‐ or left‐handed chirality into the surface of titanium carbide‐based MXene (Ti_3_C_2_T_x_) to form stable mixed‐low‐dimensional chiral MXene biomaterials with enhanced aqueous colloidal dispersibility and debonding tolerance, mimicking the natural asymmetric bio‐structure of most biomolecules and living organisms. In particular, Ti_3_C_2_T_x_ MXene nanosheets are functionalized with carboxyl‐based terminals and bound feasibly with the D/L‐cysteine amino acid ligands. The physicochemical characterizations of these 2D‐0D/1D chiral MXene heterostructures suggest the inclusion of Ti_3_C_2_T_x_ nanosheets and different levels of self‐derived MXene quantum dots and surface titanium‐oxide nanoparticles, providing enhanced material stability and oxidative degradation resistance for tested months. Further, the interaction and molecular binding at cysteine‐Ti_3_C_2_T_x_/Ti‐oxide interfaces, associated ion transport and ionic conductivity analysis, and charge re/distribution mechanisms are evaluated using density functional theory (DFT) calculations and electrochemical impedance spectroscopy (EIS) measurements. The second uniqueness of this study relies on the multifunctional application of optimal chiral MXenes as potential nano‐biostimulants for enhancing plant tolerance to different abiotic conditions, including severe drought, salinity, or light stress. This surface tailoring enables high biocompatibility with the seed/seedling/plant of *Arabidopsis thaliana* alongside promoting multi‐bioactivities for enhanced seed‐to‐seedling transition, seedling germination/maturation, plant‐induced stomatal closure, and ROS production eliciting responses. Given that the induced chirality is a pivotal factor in many agro‐stimulants and amino acid‐containing fertilizers for enhanced interaction with plant cells/enzymes, boosting stress tolerance, nutrient uptake, and growth, these findings open up new avenues toward multiple applications of chiral MXene biomaterials as next‐generation carbon‐based nano‐biostimulants in agriculture.

## Graphical abstract

An innovative strategy is showcased for inducing right/left‐handed chirality in MXene‐nanosheets and derived quantum dots to form stable mixed‐low‐dimensional heterostructured‐biomaterials with enhanced colloidal dispersibility and debonding resistance, mimicking the natural asymmetric structure of most chiral biomolecules and living organisms. Theoretically and experimentally, we elucidated surface interactions and plant‐biostimulant activity of optimal chiral MXene colloids for enhanced tolerance to abiotic stresses.

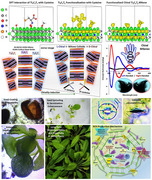

## 1 Introduction

Plants are sessile and cannot move or migrate. As such, for survival and growth, they are forced to adapt and confront various extreme environmental conditions, known as abiotic stressors, including drought, heat, and salinity, as well as light stress, such as farming under photo‐inhibition and insufficient light shade constraints. While plants have sophisticated immune mechanisms and defense responses at different cellular and tissue levels to overcome or ameliorate these challenges, exposure to severe stresses can activate various physical/chemical changes in their structures and physiological traits promptly or over time, limiting their development and yield.^[^ ^1^, ^2^, ^3^, ^4^, ^5^ ^]^ Indeed, agricultural productivity has been reported to be significantly impacted by these stressors alongside substantial losses in crucial farming staples, including *Oryza sativa*, *Zea mays*, *Triticum aestivum*, and *Hordeum vulgare*. Based on the available statistics, the yield of major food crops can be relatively reduced by ≈20–50%, and by up to 70% under severe cases and regions with specific seasonal variations, depending on the stressors’ severity and exposure duration.^[^ ^6^, ^7^, ^8^, ^9^, ^10^, ^11^ ^]^ This fact performs as a barrier against the plant's natural development worldwide, and in critical farming conditions, most of these crops can only grow at not more than their 30% genetic potential.^[^ ^7^, ^12^, ^13^, ^14^ ^]^ It is obvious that the sensitivity and resistance of plants to abiotic stress conditions vary at different types and or developmental stages, determining their susceptibility to adverse environmental influences. Therefore, stimulating/fertilizing the plants and crops with case‐specific agrochemicals is essential, which despite, their effectiveness, can be costly and pose health‐related and ecological concerns when implemented in large‐scale farming.

Over the recent decades, there has been a remarkable advancement in developing synthetic agrochemical biostimulants and amino acid‐containing fertilizers for enhancing plants’ tolerance to stressors, nutrient uptake, and growth based on specific environmental or farming conditions.^[^ ^15^ ^]^ These agrochemical management strategies have been proven effective in plants to overcome harsh abiotic stressors. However, the large‐scale implantation of agrochemical products is not without ecological concerns and has been reported to impose negative impacts on the environment, soil health, livestock, and human health, as well as the challenges associated with the production costs and operational risks for farm workers. Thus, alleviating these limitations and the pressure on agriculture have been among the global focuses of modern farming.

More recently, emerging agricultural nanotechnologies have received substantial attention to address these long‐standing challenges by designing eco‐friendlier plant biostimulants to safeguard the crops from abiotic stressors either alone or in combination, increase their yields while focusing on enhanced biocompatibility and sustainability properties alongside tunning with slower release, and reducing/minimizing their long‐term risks to human health and ecological impacts.^[^ ^16^, ^17^, ^18^, ^19^, ^20^, ^21^, ^22^, ^23^, ^24^ ^]^ In this particular context, the field has witnessed a growing interest in designing and developing newer and smarter generations of functional nano‐biomaterials in different forms, including single, hybrid, composite, and heterostructures with enhanced physicochemical properties, biological interactions, and phytoactive functionalities toward practical applications in actual agricultural settings.^[^ ^18^, ^25^, ^26^ ^]^ Consequently, the boundaries of interdisciplinary nano‐agricultural research has been expanded by rational materials‐based strategies for enhancing plant systemic resistance mechanisms against distinct abiotic stressors to efficiently boost their survival, growth, and productivity likewise the key functions of many related agrochemical stimulants, but in more eco‐efficient fashions.^[^ ^21^, ^27^, ^28^, ^29^, ^30^, ^31^, ^32^, ^33^, ^34^, ^35^ ^]^ Among them, ample attention has been given to low‐dimensional bio‐polymers such as chitosan and its derivations and carbon‐based nanomaterials like fullerenes, carbon quantum dots, carbon nanotubes, graphene nanospheres, and reduced/graphene oxide sheets due to their large specific area and programmable physicochemical/biological properties.^[^ ^36^, ^37^, ^38^, ^39^, ^40^, ^41^, ^42^, ^43^, ^44^ ^]^

MXenes are the latest‐ and largest‐discovered family of carbon‐based nanomaterials. Since the first report on the synthesis of the first MXene nanosheets in 2011, and subsequently quantum dots in 2017, they have found extensive research credits due to their multi‐functional properties.^[^ ^45^, ^46^, ^47^, ^48^, ^49^, ^50^, ^51^ ^]^ MXene nanomaterials have the general formula of M_n__+1_X_n_T_x_ (*n* = 1−4), where “M” stands for one or multiple transition metals in the periodic elements, “X” is carbon and or nitrogen, and “T_x_” represents an abundance of functional oxygen, hydrogen, nitrogen, and/or fluorine/chlorine‐based surface terminations. 2D MXenes are most commonly synthesized through top‐down methods such as wet‐acid, molten‐salt, or acid‐free selective etching of the “A” layers (the elements of group 13–14 of the periodic table) from the structure of their MAX‐phases (M_n+1_AX_n_).^[^ ^51^, ^52^, ^53^ ^]^ This “newer”, “smarter”, and “more tunable” generation of low‐dimensional materials has been also reported to possess unique negatively charged hydrophilic surface properties at different pH levels, ranging from 3 to 11, and relatively‐active surface optical absorption properties at different wavelengths.^[^ ^54^, ^55^, ^56^, ^57^, ^58^, ^59^, ^60^, ^61^, ^62^, ^63^, ^64^ ^]^

MXenes of specific compositions at controlled doses have been also reported to offer high biocompatibility with a wide range of human cells and mammalian tissues and organs, as well as living seedlings and plant systems.^[^ ^56^, ^65^, ^66^ ^]^ These MXene‐based biomaterials have also the capacities to be readily and spontaneously taken up by specific cells, tissues, and organisms without any uptake‐enhanced techniques, interacting with diverse biological systems at different levels to stimulate, inhibit, delivery, detect, and/or monitor specific biological/immunological responses related to immunity, growth and metabolism, gene expression, and signaling pathways.^[^ ^54^, ^63^, ^67^, ^68^, ^69^, ^70^, ^71^, ^72^, ^73^, ^74^, ^75^, ^76^ ^]^ Furthermore, recent studies reported the new properties of specific MXenes as potential electrode materials for water/air filtration and removal of heavy elements like mercury.^[^ ^74^ ^]^

The multifunctional bio‐properties/activities of specific MXenes have shed light on their potential for future applications in the eras of nanomedicine, applied biology, and the environment. Hence, the rationale design and utilization of MXene biomaterials in nano‐agriculture has been an emerging field with limited reports of their properties or applications thus far. Hitherto, the agricultural perspectives of MXenes (mostly titanium carbide Ti_3_C_2_T_x_) have been showcased for the sustained release of pesticides (e.g., *Avermectin* and *Emamectin*) and elsewhere as a nano‐carrier tool for delivering *β‐Cyfluthrin* insecticide.^[^ ^77^, ^78^, ^79^ ^]^ Moreover, there have been few reports on the capacity of these MXenes to be used as wearable and or wireless plant sensors for the detection of phytohormones and sensing *aflatoxin* and *malathion* in some agricultural products and ecological samples.^[^ ^80^, ^81^, ^82^ ^]^ Further, the impacts of Ti_3_C_2_T_x_ MXene are reported on the increment of *Torreya grandis*’s tolerance against lead stress by reducing its accumulation in the soil systems and protecting these plant types against root rot disease.^[^ ^83^, ^84^ ^]^ Another work studied the antioxidant properties of a Ti_3_C_2_T_x_ MXene quantum dots‐based composite as a potential scavenger for reactive oxygen species (ROS) in cotton plants.^[^ ^66^ ^]^ Moreover, we recently demonstrated an innovative plant‐immunoengineering and triple biotic antipathogenic modes‐of‐action of surface‐modified Ti_3_C_2_T_x_ MXene‐based nanosheets, as well as their impacts on eliciting, priming, and regulation of specific primary and or secondary defense‐related genes and phytohormones in mature *Arabidopsis thaliana* plants to enhance their phytopathogenic diseases resistance systemically.^[^ ^85^ ^]^ In particular, we have reported on possible activation of the induced systemic resistance (ISR) mechanisms likely through regulation of the jasmonic acid‐related pathways in model plants tested. These studies along with the other available literature have significantly expanded the scope/boundaries of MXene biomaterials in nano‐agriculture beyond conventional bioactive nanomaterials, opening up new avenues of study to advance their practical agriculture and environmental applications.

It is important to note that despite the knowledge advances obtained in the biocompatibility behaviors of MXene materials, the understanding of their long‐term biological and immunological interactions, especially with plant systems, soil biological organisms, and beneficial symbionts, is still in its infancy and early stages. Further research investigations are essential to attain detailed and precise information about their safety, optimal doses, phyto‐interactions, treatment methods, and effective time points. This is time‐consuming and cost‐effective due to the nature of research on living plants and cultivar settings. Hence, designing facile and efficient strategies to increase the biocompatibility of MXenes to their higher levels alongside improving their long‐term colloidal stability in aqueous media while maintaining their key characteristics is a nascent materials science technology, and highly beneficial for nano‐agriculture. One of the promising approaches is tailoring the surfaces of MXenes by bioactive molecules to simultaneously enhance their biocompatibility and reduce susceptibility to oxidation, degradation, and or decomposition in aqueous media.

From agricultural point of view, it is essential that the desirable surface functionalization support high levels of phyto‐compatibility and activities to effectively interacting with the living plants and beneficial soil organisms. Chirality is a pivotal factor in the biological and biochemical properties of many bio‐molecules and living systems.^[^ ^86^ ^]^ In particular, the amino acids, DNA, RNA, proteins, carbohydrates, lipids, hormones, and cell metabolites are chiral, or in most cases, have innate chirality in their complex structures.^[^ ^87^, ^88^, ^89^ ^]^ Given this, the chirality aspect has been credited in the production of specific commercial agrochemical fertilizers and plant stimulants by inducing the asymmetric structure into their original patterns for a similar concept of improving surface interactions and biocompatibility of these products with the surrounding bio‐environments.

Indeed, chirality plays a significant and crucial role in living biological systems, influencing their physiological processes and the specificity of interactions. A key phenomenon on the importance of chirality is that molecular symmetry affects biological mechanisms, as most of the molecular components of living organisms intrinsically possess chiral properties. Receptors, regardless of their functions in bio‐systems, enable living organisms to recognize, perceive, and regulate all physicochemical and electrical changes that occur in their surrounding environments. Because receptors naturally consist of chiral molecules have a higher tendency to interact and bind with bio‐substances or synthetic stimulant biomaterials, which are enantiomeric, or have induced chiral asymmetric structural characteristics. For this reason, synthesizing chiral‐active biomaterials as biostimulants may not only improve their microstructure and stability aspects, but also desirably enhance their functional properties, interactions, and compatibility with biological systems.

From the plant biology perspective, it is well understood that even though plants do not exhibit apparent asymmetry in their physiological shapes, organ arrangement, or composing cells, plants produce various chiral bio‐molecules, including alkaloids, flavonoids, and terpenes, which play significant roles in their growth, signaling processes, and defense mechanisms. Therefore, plants enable chiral characteristics, which for instance, contribute to developing radially symmetric growth. Considering these accounts, it may support the phenomenon that plants behave compatible with chiral‐active molecules and interact better with them. Hence, synthetic biomaterials with induced chirality may reasonably possess good phyto‐compatibility along with a reduced risk of toxicity upon direct contact with plants. This biocompatibility may subsequently support their interaction for functional bioactivity mechanisms in plants. This rationale might be one of the key aspects behind the development of chiral‐active agro‐stimulants or fertilizers. Designing highly biocompatible chiral materials with large active surface areas is therefore at the forefront of the field toward developing plant nano‐stimulants and stress resistance promoters once they have proven sufficiently safe for agriculture.

With this in mind, in the current study, we designed and developed stable right‐ or left‐handed chiral MXene‐based biomaterials with improved aqueous colloidal dispersibility for enhancing plant tolerance to abiotic stressors. These chiral MXene colloids showed high biocompatibility with living plants at tested concentrations; and effectively induced *in‐planta* stomatal closure and ROS generation mechanisms to boost alertness and tolerance to drought, salinity, and light‐stress. To our knowledge, this study is the first report on the design, development, and application of chiral MXene biomaterials to enhance plant tolerance to different abiotic stresses. These findings are expected to contribute to establishing new paradigms toward practical applications of chiral MXenes, especially as next‐generation nano‐biostimulants in agriculture. To highlight the novelty and significance of this work, we presented a comprehensive view of the previously reported synthetic chiral materials (Table S1, Supporting Information). This tabulated literature encompasses the most available chiral‐induced materials (except and other than MXenes), including conventional nano‐carbons and metal‐organic frameworks.

## 2 Results and Discussion

### 2.1 Schematic Illustration of the Surface Functionalization and Chirality Induction

**Figure 1:**
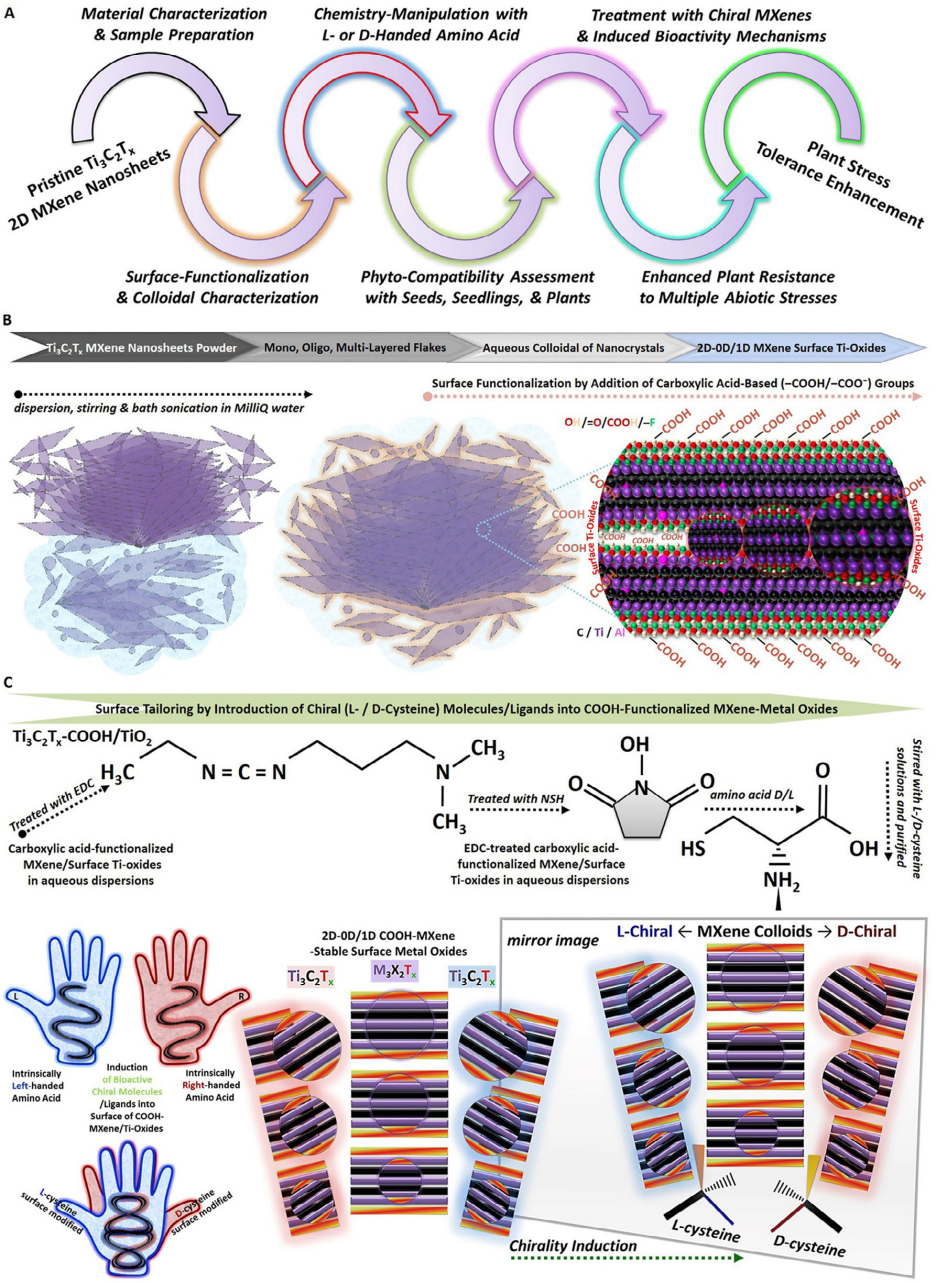
The work plan and schematic illustration of converting 2D Ti_3_C_2_T_x_ MXene sheets to right‐ or left‐handed mixed‐low‐dimensional chiral MXene heterostructures. A–C) The cartoon model depicting the study's step‐by‐step workflow from design to phyto‐applications, including the surface functionalization and right‐/left‐handed chirality induction in the structure of carboxyl‐modified Ti_3_C_2_T_x_ colloids in aqueous colloidal suspensions using EDC/NHS (C_8_H_17_N_3_ · HCl / C_4_H_5_NO_3_) crosslinking.

A schematic overview of the study's workflow, preparation, and functionalization processes, as well as an atomic structure model and synthesis route for surface/chemistry tailoring of Ti_3_C_2_T_x_ nanosheets to create stable mixed‐low‐dimensional chiral MXene heterostructures in the form of aqueous colloidal dispersions are demonstrated in **Figure** **1**A–C. The applied magnetic‐stirring and bath‐sonication of these pristine accordion‐like MXene flakes assist in the partial delamination and further expansion of the dispersed flakes alongside imposing a gradual relative surface oxidation, including titanium oxides. Notably, the subsequent treatment of these dispersed mono‐, oligo‐, and multi‐layers in pure MilliQ water, followed by the applied bath‐ or probe‐sonication of the mixtures in different ratios of citric acid solutions, contributes to effectively functionalizing the surface of these nanosheets in all groups with carboxyl‐based surface functional terminations (e.g., –COOH, −COO⁻, C═O). This process results in the formation of colloidal mixtures containing 2D Ti_3_C_2_T_x_ sheets and self‐derived 0D MXene quantum dots with stable decorations of 1D surface titanium‐oxide nanoparticles clustered at different ratios in their final compositions.

Subsequently, the stable induction of right‐ or left‐handed chirality was applied and introduced into the surface of these functionalized Ti_3_C_2_T_x_ colloids through *N*‐(3‐Dimethylaminopropyl)‐*N*‐ethylcarbodiimide hydrochloride/N‐hydroxysuccinimide (EDC/NHS) method as an established universal cross‐linking with a slight modification and parameters adjustment. This facile surface‐modification approach feasibly enables the stable bonding of the bioactive chiral molecules (here, D‐/L‐cysteine amino acids) on the surface of MXene nanosheets and self‐derived low‐dimensional particles, contributing to enhancing the dispersibility and stability of pristine Ti_3_C_2_T_x_ nanosheets against oxidation in aqueous media. Additionally, these specific chiral MXene biomaterial designs have shown the efficacy of being customized into other forms beyond the aqueous colloidal dispersions, including spin‐coated films and vacuum‐dried flakes at around room temperature. Using facile methodology and without the necessity of complex laboratory equipment, the right‐ or left‐handed chirality was innovatively introduced to these MXenes’ surfaces, creating 2D‐0D/1D mixed‐low‐dimensional heterostructures for the targeted plant biostimulation applications in nano‐agriculture. Table S2 (Supporting Information) presents the labeling of as‐prepared chiral MXenes (B0.3L, B0.3D, B0.15L, B0.15D, P0.15L, P0.15D, P1L, and P1D) based on the applied treatments, parameters, and the concentration of additive reagents.

### 2.2 Proposed Reactions for Converting 2D MXene Nanosheets to 2D‐0D/1D Chiral MXenes

To further elaborate on the underlying chemical reactions and mechanism associated with the conversion of 2D Ti_3_C_2_T_x_ MXene nanosheets to mixed‐dimensional heterostructure and formation of 2D‐0D/1D chiral MXenes, we proposed a step‐by‐step stoichiometry reactions based on three primary equations. In particular, as shown in the proposed reactions 1 to 3 and Figure S1 (Supporting Information), Ti_3_C_2_T_x_ MXene nanosheets interact with the air‐available or dissolved oxygen molecules in water and subsequently bond with citric acid monohydrate (C_6_H_8_O_7_ · H_2_O), EDC (C_8_H_17_N_3_ · HCl), NHS (C_4_H_5_NO_3_), and D‐/L‐cysteine amino acid (C_3_H_7_NO_2_S) in aqueous solutions as below.

- **Step 1**: Dispersion, oxidation, and hydrolysis reaction:

The step involves the formation of titanium oxide (e.g., TiO_2_) on the surface of the MXene flakes. The current reaction is primarily a surface interaction rather than a permanent covalent bond, but it forms a reasonably stable structure in water at room temperature.

(1) $$Ti_{3}C_{2}T_{x}+H_{2}O+O_{2}\to Ti_{3}C_{2}T_{x}/\mathrm{Ti}O_{2}+H_{2}$$

- **Step 2**: EDC/NHS activation and cross‐linking reaction:

The hydroxyl (−OH) and carboxyl groups (−COOH) on the surface of Ti_3_C_2_T_x_/TiO_2_ are increased through treating with citric acid monohydrate and activated using the EDC and NHS cross‐linking reagents. EDC activates the ‐COOH groups by forming an O‐acylisourea intermediate, while NHS (C_4_H_5_NO_3_) stabilizes the intermediate by forming an NHS ester (−O−C_4_H_4_NO_3_). It prevents further hydrolysis of the intermediate and ensures efficient coupling. The active surface of Ti_3_C_2_T_x_/TiO_2_ can then interact with other functional groups through a Ti−O−C bond.

(2) $$\begin{array}{c} Ti_{3}C_{2}T_{x}/\mathrm{Ti}O_{2}-\mathrm{COOH}+\mathrm{EDC}+\mathrm{NHS}\to \\ Ti_{3}C_{2}T_{x}/\mathrm{Ti}O_{2}-C(=O)-O-C_{4}H_{4}NO_{3} \end{array}$$

- **Step3**: Chiral functionalization with D‐ or L‐cysteine amino acid ligands:

The NHS ester on the surface of Ti_3_C_2_T_x_/TiO_2_ reacts with the amine group (–NH_2_) of D‐/L‐cysteine. This reaction forms an amide bond (C−N), where the NHS group is displaced and released into the solution as a water‐soluble by‐product. The reaction includes the C−N, formed between the amine group of D‐ or L‐cysteine and the activated carboxyl group. Further, there is a possible covalent bond between the titanium surface and these functional groups (Ti−O−C).

(3) $$\begin{array}{c} Ti_{3}C_{2}T_{x}/\mathrm{Ti}O_{2}-C(=O)-O-C_{4}H_{4}NO_{3}+C_{3}H_{7}NO_{2}S\to \\ Ti_{3}C_{2}\mathrm{Tx}/\mathrm{Ti}O_{2}-C(=O)-\mathrm{NH}-C_{3}H_{7}NO_{2}S+C_{4}H_{5}NO_{3} \end{array}$$

The final chiral MXene products, therefore, contain Ti‒O‒C and C‒N covalent bonds with the D‐ or L‐cysteine molecules (Ti_3_C_2_T_x_/TiO_2_−C(═O)−NH−C_3_H_7_NO_2_S). The by‐product includes the displaced NHS group, which forms water‐soluble C_4_H_5_NO_3_ that could be thoroughly washed away from these colloidal solution systems during the purification stage, creating chiral MXenes with an almost similar pH to the pure water.

### 2.3 Phase Analysis and Purity of Surface‐Functionalized and Chiral MXenes

In the first microstructural characterization, we evaluated the phase purity of these materials to confirm the success of applied surface functionalization of pristine Ti_3_C_2_T_x_ MXene nanosheets in aqueous dispersions and provide a comparative overview picture of their crystallization patterns. The grazing incidence X‐ray diffraction (GIXRD) was selected over conventional XRD due to the mixed‐low‐dimensional characteristic of these samples, which are colloidal dispersions of treated Ti_3_C_2_T_x_‐based nanosheet powder in pure MilliQ water. As a consequence of these processes, it was expected that 2D flakes of Ti_3_C_2_T_x_ MXene are surface‐decorated with self‐assembly of 0D quantum dots and modified with stable 1D titanium oxides nanoparticles, forming a 2D/0D‐1D heterostructures. Thus, it was essential to preserve the nature of samples and analyze them in their colloidal states without significant agglomeration and avoid alterations that likely arise during drying into powder. To ensure an accurate assessment of their phase purities, we have spin‐coated the aqueous colloidal dispersions on a popular glass coverslip substrate at room temperature to maximize the GIXRD's sensitivity to identifying the crystalline patterns of the obtained thin‐film surface layers with reduced risk of collocating obscured phase information from bulk analysis and unwanted oxidation during sample preparation. Furthermore, for a robust comparison, the commercial powders of pristine Ti_3_C_2_T_x_, D‐/L‐cysteine, and citric acid were phase‐characterized by sticking them on the slide substrates using commercial high‐vacuum amorphous silicon grease.

**Figure 2:**
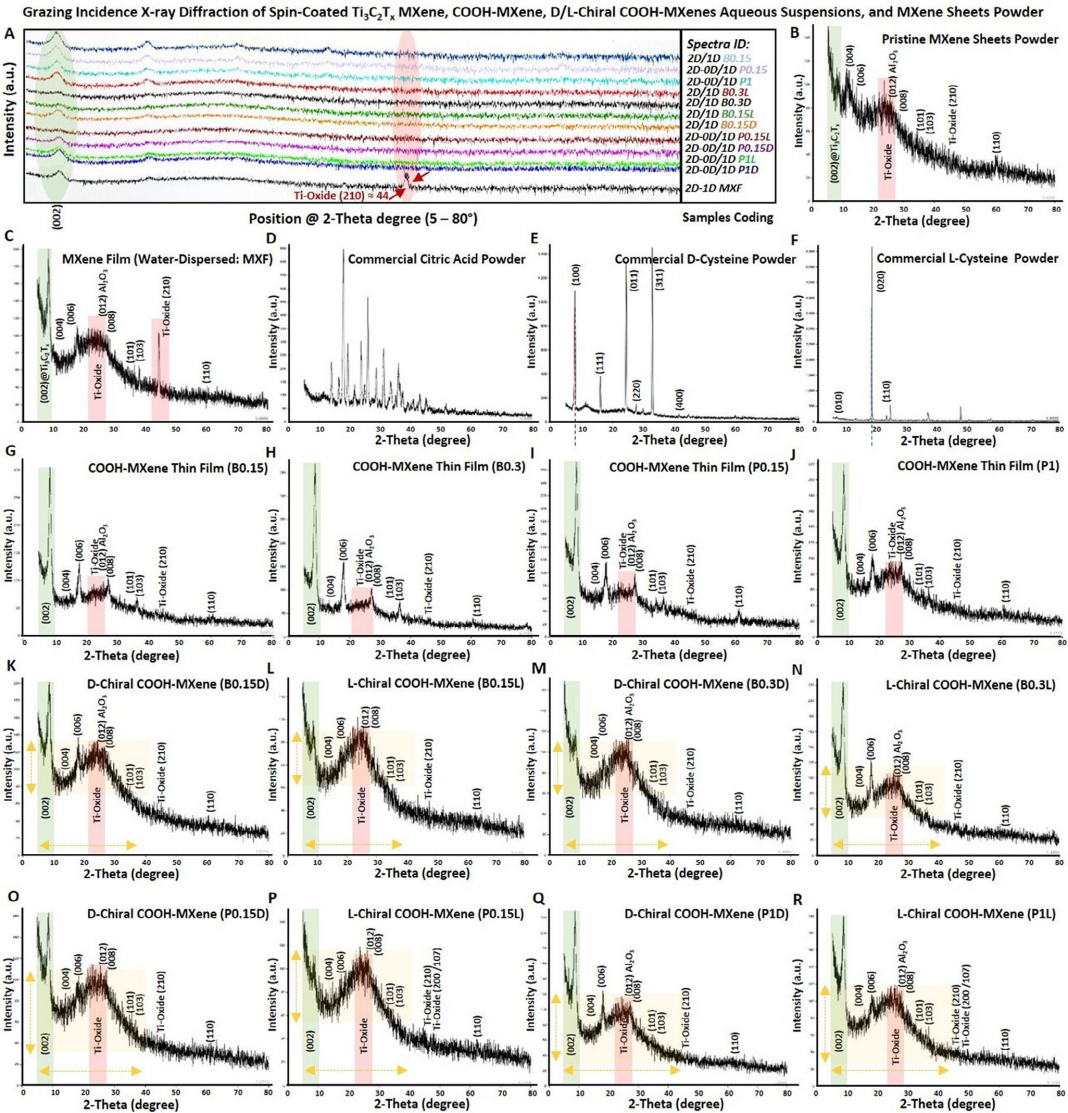
GIXRD phase characterization of pristine Ti_3_C_2_T_x_ MXene nanosheets powder, carboxyl‐functionalized MXene, and chiral COOH‐functionalized Ti_3_C_2_T_x_ MXene‐based colloid in the form of thin films. A) The merged spectra of these MXene‐based samples aqueous colloidal dispersions spin‐coated thin films in the form of thin films, B) pristine Ti_3_C_2_T_x_ MXene powder and C) the nanosheets dispersed in MilliQ water overnight and spin‐coated on glass substrate. D–F) GIXRD of the commercially available L‐cysteine, D‐cysteine, and citric acid powders that have been loaded/stacked on the glass coverslip using high‐vacuum amorphous silicon grease. G–J) GIXRD crystalline patterns of the bath‐ and probe sonication‐treated MXenes with different molarities of citric acid after spin‐coating and drying at room temperature. K–R) The GIXRD patterns of these carboxyl‐functionalized samples at different concentrations of citric acid, with and without surface attachment with the right‐ or left‐handed chiral amino acid ligands. The characteristic (002) peak of in the GIXRD spectra of these samples shows the identification of the typical phase structure of Ti_3_C_2_T_x_ MXene.

As shown in **Figure** **2**A–C, the GIXRD spectra of these MXene‐based film materials depicted a minor identification of aluminum or aluminum oxide‐based phases, suggesting their significant elimination from the pristine structure of the used Ti_3_C_2_T_x_ MXene nanosheets powders. Also, the phase characteristic (002) peaks were detected in the GIXRD analysis of these nanosheets at 2θ of ≈8°. Additionally, the crystalline patterns of these samples confirmed the detection of a relatively lower intensity peak at 2θ ≈ 39°, which typically corresponds to the parent Ti_3_AlC_2_ MAX‐phase structure of these nanosheets that together suggests the quality of this base material with a negligible composition of the aluminum layers. However, it is notable that in the GIXRD spectrum of these flakes, when dispersed in pure water overnight, there are identifications of oxide peaks mainly assigned to titanium dioxide (TiO_2_) at 2θ ≈ 25°, which is at a higher level compared to the powder of these Ti_3_C_2_T_x_ MXene nanosheets.

This higher oxidation and material degradation that are likable over time by dispersing in water media or exposure to the air during sample preparation, storage, and measurement processes result in the appearance of a dominant peak at 2θ ≈ 44°. This innate tenancy of 2D MXenes for oxidation has been previously reported in different studies and many strategies, including surface modification, ion intercalation, and antioxidant‐agent composition, have been designed and developed accordingly to enhance the stability of MXene nanosheets, especially for colloidal applications.^[^ ^90^, ^91^ ^]^ The reference spectra of the original D‐ and L‐cysteine and citric acid are represented in panels D to F.

Further, as can be seen in Figure 2G–R, the GIXRD analysis of these carboxyl‐functionalized Ti_3_C_2_T_x_ MXene‐based colloids, once stored at 4° for ≈30 days and then spin‐coated as thin films on glass substrates, still showed the characteristic peaks of MXene, including the (002) at the similar positions of 2θ ≈ 8° as the dominant phase with almost a same peak intensity in the GIXRD spectra of these materials, suggesting their enhanced colloidal stability against oxidation in water, compared to the pristine Ti_3_C_2_T_x_ MXene nanosheets (see panels G‐J). Interestingly, the assessment of phase patterns of these samples after surface modification with two chiral amino acid ligands at different doses showed almost similar crystalline structures of Ti_3_C_2_T_x_ and surface oxides (panels K‐R). Notably, a slight shift to the lower 2θ values (left‐side shift) in the (002) peaks of these carboxyl‐functionalized Ti_3_C_2_T_x_ and chiral Ti_3_C_2_T_x_‐based MXenes is observed, which may be attributed to a slight increase in the crystal lattice and interplanar spacing (d‐spacing) of the surface‐modified materials compared to pristine Ti_3_C_2_T_x_ MXene.

Moreover, our data suggest that no obvious differences were observed in the GIXRD spectra of these samples based on the right‐/left‐handed nature of D‐cysteine and L‐cysteine amino acids. Rather, there was a minor difference in the intensity and broadness of the peak located at the 2θ of ≈10 to 30°, which likely refers to different levels of surface oxidation and relative presence of Ti_3_C_2_T_x_ quantum dots and Ti‐oxides in probe‐sonicated samples (P.015, P1, P0.15D, P0.15L, P1D, P1L) in comparison to those that have been bath‐treated (B.015, B.03, B0.15D, B0.15L, B0.3D, B0.3L). These data further highlight the stability, processability, and material resistance against oxidative degradation after surface binding/coating with these chiral biomolecules for higher durability and bioactivity upon interaction with plant and soil environments.

### 2.4 Morphology and Chemical Elemental Composition of Chiral MXenes

**Figure 3:**
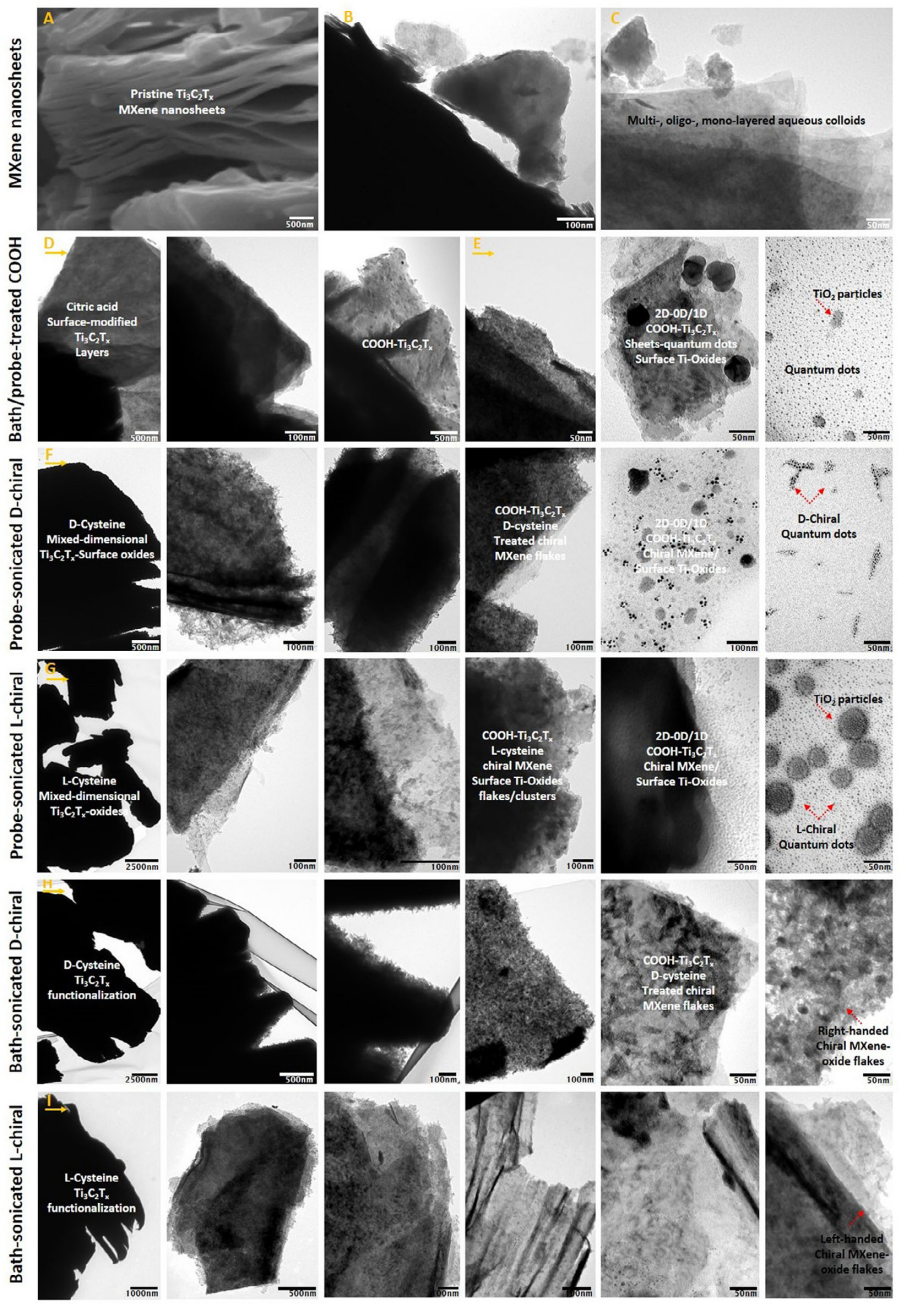
SEM/TEM morphological characterization of pristine Ti_3_C_2_T_x_ MXene nanosheets, carboxyl‐functionalized MXenes, and chiral COOH‐Ti_3_C_2_T_x_ MXene‐based aqueous colloids. A–C) The SEM and TEM images of planar Ti_3_C_2_T_x_ MXene flakes. The TEM images of D) Bath‐sonication, and E, probe‐sonication treated carboxyl functionalized MXene nanosheets and surface titanium oxides. F,G) Morphology of probe‐treated chiral Ti_3_C_2_T_x_ MXene‐based aqueous colloids after surface modification with D‐/L‐cysteine. They have been found to possess 2D‐0D/1D mixed‐low‐dimensional structures, including MXene sheets and derived quantum dots, and stable titanium oxide particle clusters. H,I) TEM images of the bath‐sonication treated chiral Ti_3_C_2_T_x_ colloids. Our assessments of these probe‐/bath‐sonication‐treated samples show an innovative assembly of chiral MXene sheets/quantum dot heterostructures with different surface oxidation ratios (e.g., TiO_2_).

Next, we evaluated the morphology and chemical elemental composition of samples. As shown, **Figure** **3**A represents the scanning electron microscopy (SEM) image of pristine Ti_3_C_2_T_x_ MXene‐based nanosheets with a well‐defined accordion‐like morphology of this material and typical layer separation. As shown in panels B and C, the transmission electron microscopy (TEM) micrographs of these nanosheets, when dispersed in ultrapure MilliQ water, exhibit the structure of pristine Ti_3_C_2_T_x_ sheets, and the applied bath‐sonication formed mono‐, oligo‐, and multi‐layered colloids of this MXene with different flake sizes and surface titanium oxides. The different magnifications TEM images accordingly characterize these MXene‐based colloids after surface functionalization with the carboxyl‐based terminations (e.g., −COOH) and after chirality induction with the two distinct sources of cysteine‐based amino acids. Figure 3D,E demonstrates the TEM images of citric acid monohydrate‐treated samples with bath‐ or probe‐sonication, respectively. As can be seen, the nanosheet structure of Ti_3_C_2_T_x_ flakes has been retained, along with the decoration of different levels of self‐derived MXene quantum dots and in situ formation of titanium oxides.

**Figure 4:**
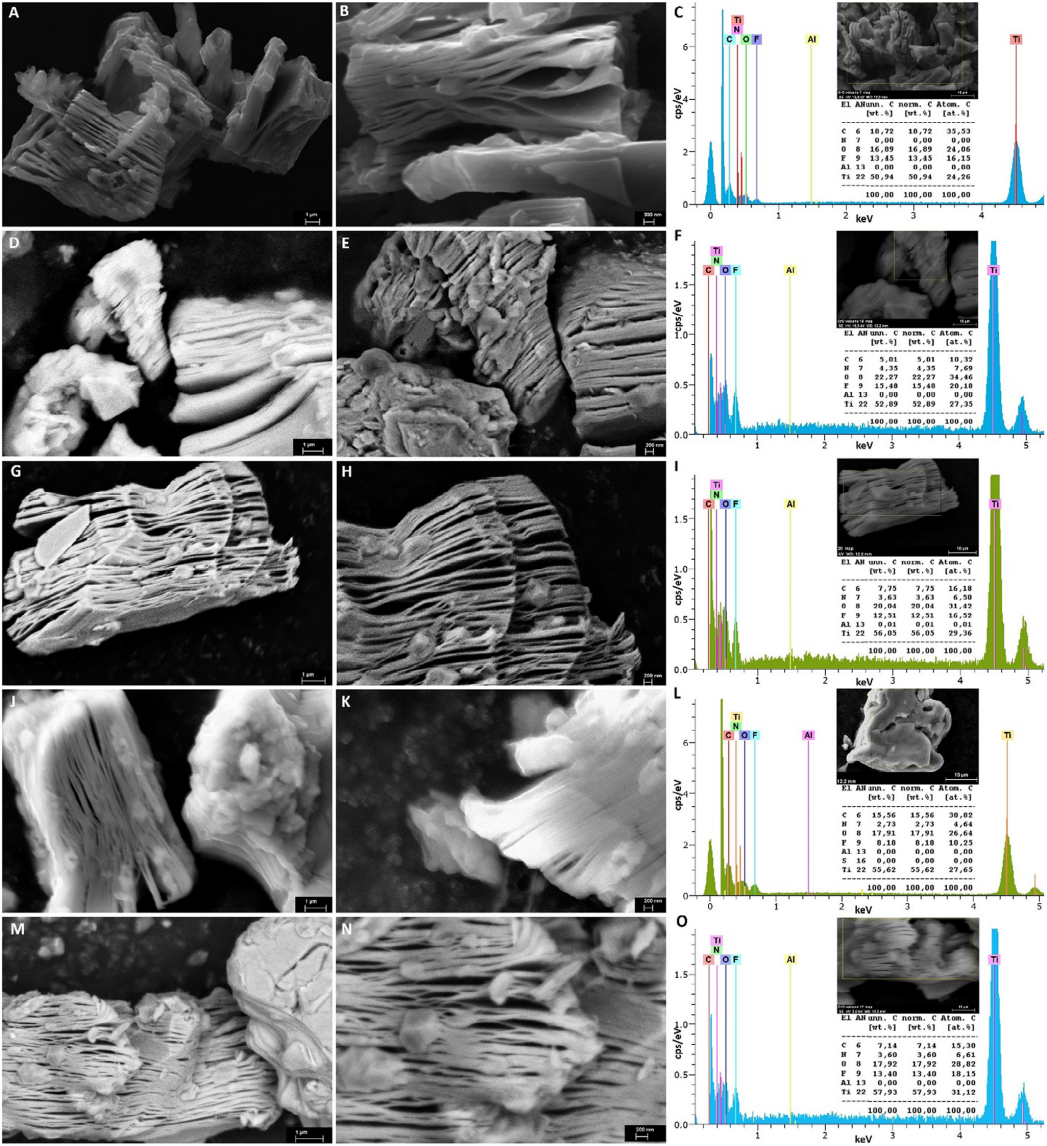
SEM/EDS microstructural characterization of Ti_3_C_2_T_x_ MXene and derived right‐ and left‐handed chiral MXene heterostructures. SEM images at different magnifications and the corresponding EDS area scan analysis of A–C) pristine MXene nanosheets, D–F) B0.15D, G–I) B0.15L, J–L) P0.15D, and M–O) B0.15L aqueous colloids after spin‐coated on glass coverslips. These data suggested the successful formation and morphology of mixed‐low‐dimensional 2D‐0D/1D structures, including carboxyl‐functionalized Ti_3_C_2_T_x_ MXene sheets, self‐derived MXene quantum dots, and stable titanium oxide particles in cluster forms on and between the surface of these MXene layers. Furthermore, the elemental chemical characterization of these samples before and after surface modification and D‐/L‐chirality induction. The chemical composition of pristine Ti_3_C_2_T_x_ MXene nanosheets showed a well‐defined typical microstructure of this material with a partial formation of non‐stable titanium oxides on the surface of these nanosheets after dispersion in water and stored overnight. The analysis of these materials (weight and atomic ratio percentage) revealed the detection of carbon, oxygen, titanium, and fluorine as dominant elements in their structures and a considerable presence of nitrogen in chiral samples due to amino acid attachment.

The morphology of these carboxyl‐functionalized colloids treated with citric acid at different molarities was also characterized after inducing the right‐ or left‐handed chirality using D‐ or L‐cysteine amino acid. As shown in Figure 3F–I, the molecular attachment of these chiral ligands on the surface of Ti_3_C_2_T_x_ colloids did not impose any significant changes in the layered architecture of these materials. Instead, the induced chirality on carboxyl‐modified MXenes resulted in further surface modification compared to non‐functionalized nanosheets. This morphological observation alongside the presented TEM micrograms in Figures S2–S4 (Supporting Information), and SEM images and associated energy dispersive X‐ray spectroscopy (EDS) data in **Figure** **4**, suggest the formation of higher Ti_3_C_2_T_x_ quantum dot clusters and further decoration of surface titanium oxide particles on and in‐between these MXene nanosheets in the group of flakes treated with probe sonication compared to the MXene samples in the bath‐sonicated groups. This difference might be due to the hydro‐mechanical vibration and homogenizer‐like effects of the applied probe‐sonication process. Rather, it contributes to enhancing the aqueous stabilization and colloidal dispersibility properties of these chiral MXene heterostructures (Figure S5, Supporting Information). It is notable that despite the ordinary dispersion of the powder of 2D MXene nanosheets in pure water and shelf‐storage, which can itself impose unwanted surface oxidation and gradual material decomposition, modifying their surfaces with the employed right‐ or left‐handed chirality induction not only effectively decreases the over/reaction of titanium traces with the water‐dissolved oxygen atoms but also contributes to enhancing their material's stability and intrinsic bio‐compatibility/activity properties due to surface coating with these bioactive chiral molecules, which is potentially beneficial for improving their interactions with plants and bio‐stimulatory activities to stresses toward agricultural applications.

Moreover, it is worth noting that this versatile methodology can also be considered to obtain quality chiral MXene‐based quantum dots for colloidal applications with more size sensitivity. In particular, as shown in Figure S6 (Supporting Information), by centrifuging these probe‐sonication‐treated aqueous colloidal suspensions (P0.15D, P0.15L, P1D, or P1L) at a moderate spinning rate of ≈1500 rpm for ≈5 min, and separating the supernatants form bigger flakes of 2D Ti_3_C_2_T_x_ and 1D Ti‐oxides in the precipitates, highly pure dimensionless aqueous chiral MXene quantum dots with an average particle diameter of less than 10 nm can be obtained. These quantum dots have several advantages over typical MXene quantum dots obtained by the conventional hydrothermal processes, as an alternative more eco‐economically‐friendly protocol, omitting the use of Teflon‐lined and other autoclave reactor equipment. The resultant chiral quantum dots by this method have potentially a lower susceptibility to degradation or gradual decomposition due to the omission of high‐temperature steam treatment or hydrothermal autoclave techniques on surface defect effects; thereby, facilitates the dimension‐reduction of 2D MXenes to their highly stable 0D quantum dots. This enhancement in the stability of representative right‐ or left‐handed chiral quantum dot colloids was tested by ultraviolet‐visible (UV–vis) optical absorption spectroscopy and camera imaging of these aqueous colloids after ≈60 days of their initial preparation, where the samples were stored in dark at four degrees (see Figure S7, Supporting Information).

Next, we performed EDS area scanning analysis to characterize the elemental compositions of the samples before and after chirality induction. As shown in Figure 4 panel C, the EDS line‐ and area‐scan analysis of pristine Ti_3_C_2_T_x_ MXene detect the presence of carbon, titanium, oxygen, and fluorine, as the primary elements in the structure of these nanosheets with a negligible atomic concentration percentage of aluminum in their compositional structure. In addition, as shown in the EDS spectra and elemental analysis of the probe‐ and bath‐sonication‐treated chiral MXenes, the presence of nitrogen with an atomic % of 4.64 and 6.61 was identified in the analysis of P1D and P1L, respectively. These ratios were found to be 7.69 and 6.50 in the EDS analysis of B0.15D and B0.15L samples, respectively. These data suggest that the induced chirality functionalization had no significant effect in the elemental compositions of Ti_3_C_2_T_x_ MXene sheets when surface‐treated with D‐/L‐cysteine amino acid. The comparison of atomic % of these samples also suggests that the probe‐sonication‐treated MXenes showed a considerably higher average oxygen ratio (P1D: 26.64% and P1L: 28.82%) compared to those bath‐sonication‐treated nanosheets (B0.15D: 34.46% and B0.15D: 31.42%). This increased oxygen concentration may be attributed to the relatively higher formation of Ti_3_C_2_T_x_ quantum dots, surface titanium oxides, and carboxyl terminations in P1D and P1L heterostructures. These data suggest that all of these chiral MXene aqueous colloids, when spin‐coated and dried on a coverslip glass substrate, have relatively higher atomic and weight percentages of oxygen compared to the pristine Ti_3_C_2_T_x_ nanosheets, when dispersed in pure water, suggesting the higher presence of stable surface functional groups in the structure of these mixed‐ dimensional MXenes. These SEM/EDS data of these samples agree with their TEM observations, suggesting surface modification of 2D Ti_3_C_2_T_x_ in both right‐ and left‐handed chiral MXene groups.

### 2.5 Colloidal Dispersibility, Left‐/Right‐Handed Chirality, and Surface Chemistry Characterization

**Figure 5:**
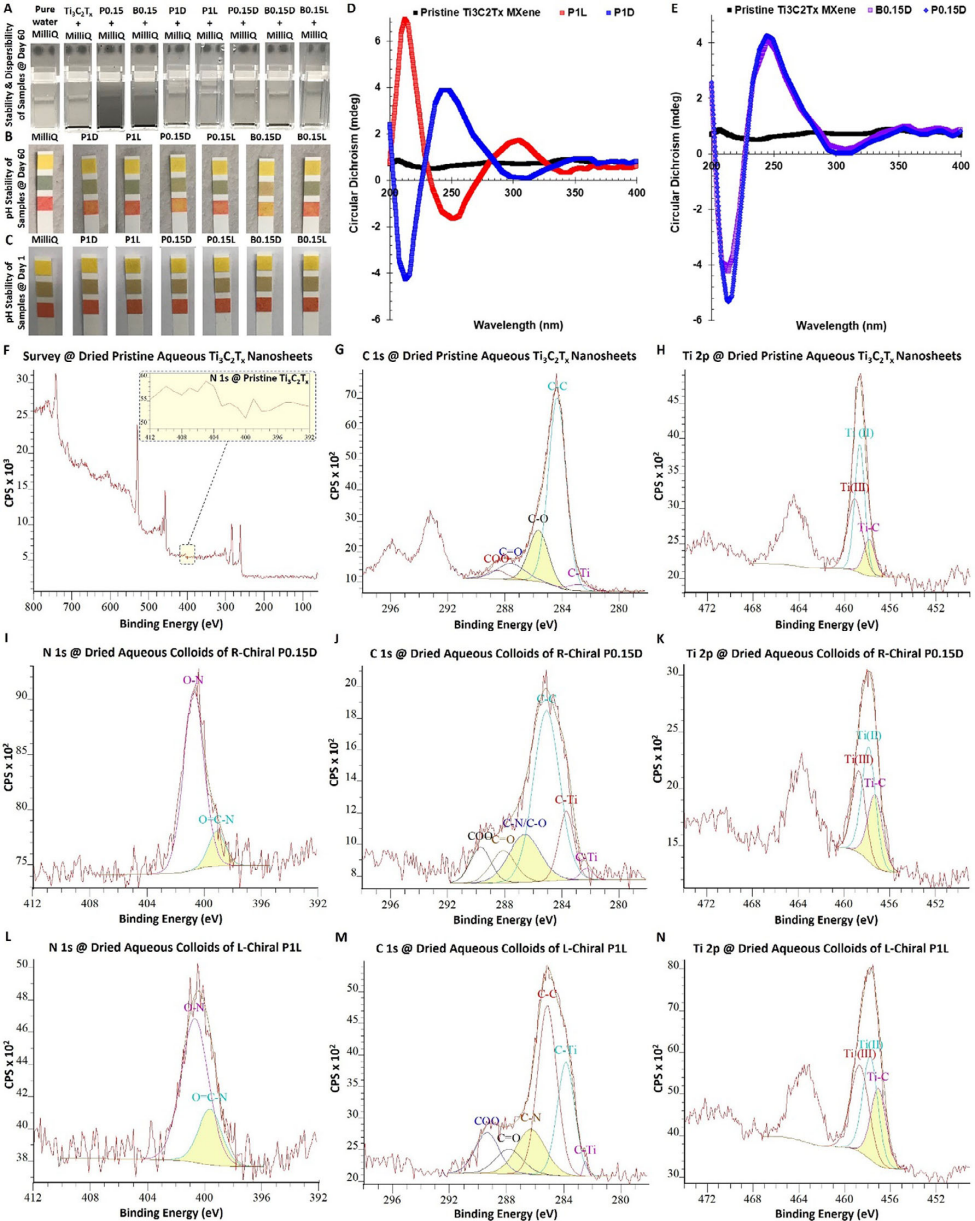
Colloidal dispersibility, left‐/right‐handed chirality, and XPS surface chemistry characterization of the dried aqueous dispersions of pristine Ti_3_C_2_T_x_ MXene nanosheets and D‐/L‐chiral carboxyl‐functionalized Ti_3_C_2_T_x_ MXene heterostructures. A–C) Camera images of long‐term colloidal dispersibility and pH‐compatibility characterization of the samples before and after carboxyl functionalization and chirality induction. These data suggest the relatively enhanced colloidal dispersibility of these modified MXenes in aqueous media. These observations on day ≈60 of materials dispersion/preparation suggest the remarkable improvement in the colloidal properties of these chiral MXene colloids with a water‐similar pH profiles. D,E) The CD spectra of P1D, P1L, B.015D, and P0.15D compared to pristine MXenes. The long‐term stability and durability of applied chirality induction into MXene were characterized after around six months, where the prepared stocks of the materials were stored at 4 degrees. F–H) The XPS survey spectra and narrow‐scan fitting of N 1s, C 1s, and Ti 2p, dispersed Ti_3_C_2_T_x_ MXene flakes in water and dried on the sample holder. The narrow‐scan fitting of N 1s, C 1s, and Ti 2p of the representative right‐handed I–K) P0.15D chiral MXene colloids, and left‐handed L–N) P1L MXene collides. These data suggest the surface functionalization and modification of Ti_3_C_2_T_x_ nanosheets to form highly dispersible 2D‐0D/1D chiral MXene heterostructures containing MXene nanosheets, self‐derived quantum dot, and stable titanium oxide particles with different ratios of surface oxidation.

In the next experiment, we evaluated the colloidal dispersibility and pH neutrality profile of these MXene heterostructures compared to MilliQ water. Our assessments of long‐term colloidal characterization of these samples are presented in **Figure** **5**A. As can be seen, all the tested chiral MXenes exhibit a relatively enhanced aqueous colloidal dispersibility, when compared to pristine Ti_3_C_2_T_x_ flakes powder at an almost similar concentration in water (≈650 µg mL^−1^). It is notable that after surface functionalizing with −COOH, this property is found to be slightly improved with some precipitation remaining across the samples in both bath‐ and probe‐sonicated MXene sheets. However, our observation suggested a remarkable improvement in the dispersibility of these Ti_3_C_2_T_x_ MXenes after inducing the right‐ or left‐handed chirality (P1D, P1L, B0.15D, and B0.15L). Furthermore, our pH characterization of these aqueous colloids depicts no significant differences compared to MilliQ water after around two months of initial stock preparation (see panels B, C).

To confirm the induced right‐ or left‐handed chirality in MXene nanosheets, we performed the circular dichroism (CD) analysis of the prepared materials. In particular, we measured the difference in absorption of right‐ or right‐handed circularly polarized light by the effect of chiral molecules. To do so, we tested representative MXenes in both D‐ and L‐chiral colloids at the same concentration and after storing for over six months at 4 degrees. As shown in Figure 5D, no circular optical activity (non‐chiral symmetric behavior) was observed in the CD spectra of pristine Ti_3_C_2_T_x_ dispersions. However, the analysis of P1D and P1L MXenes showed an obvious right‐ or left‐handed chirality (asymmetric profile) in both tested samples, suggesting a stable chirality induction in the structure of these samples. In particular, these CD spectra revealed bands at ≈210–220, 250, and 305–310 nm that changed their peak signs for different right‐/left‐handed chirality of the edge ligands of D‐ or L‐cysteine amino acid‐treated MXene. The positive and negative CD bands correspond to the left‐/right‐helicity of chiral MXenes has found to be quite stable in these samples. To further confirm this chemistry manipulation, the CD measurements were also conducted for different representative B0.15D and P0.15D chiral MXenes (see Figure 5, panel E). Interestingly, our observation suggested the induction of highly stable chirality in all tested samples regardless of the applied bath or probe‐sonicated treatments.

Next, we performed X‐ray photoelectron spectroscopy (XPS) analysis to further characterize the surface chemistry of pristine Ti_3_C_2_T_x_ and prepared chiral MXene heterostructures. The surface of representative left‐ and right‐handed chiral MXenes was analyzed using XPS wide and narrow‐scan analysis. The XPS C 1s, Ti 2p, and N 1s fittings show the successful surface‐functionalization of MXene with carboxyl groups and the presence of nitrogen‐based surface terminations, such as amine groups, formed by the applied treatment with D‐/L‐cysteine molecule. The XPS assessment of these colloids, when spun down and dried the precipitants under Argon, suggest a considerable change in the atomic percentage of carbon and titanium compared to the Ti_3_C_2_T_x_ flakes alongside the detection of newly‐emerged nitrogen‐containing surface compounds in the atomic structures of chirality‐introduced colloids after amino acids modification. As shown in panels F‐N of Figure 5 and Figure S8 (Supporting Information), the wide‐scan survey of typical Ti_3_C_2_T_x_ MXene flakes represents a typical characteristic of their surface compositions, including C 1s, Ti 2p, O 1s, and F 1s with a negligible presence of Al 2p at the binding energies of ≈50 to 800 eV. As shown, this data clearly demonstrates the absence of nitrogen content in the composition of pristine Ti_3_C_2_T_x_ MXene. Figure 5G presents the C 1s spectrum of these flakes after being submerged in water. The results indicate that the C−Ti bond in this sample (≈283 eV) is relatively weak, suggesting the potential debonding of titanium from carbon, followed by unstable oxidation to form TiO_2_. This tendency and characteristic behavior is expected for MXene nanosheets after prolonged exposure to water. Additionally, as illustrated in panel H, the narrow‐scan Ti 2p analysis of this sample reveals peaks corresponding to Ti−C, Ti(II), and Ti(III) at the binding energies of ≈456, 457, and 459 eV, respectively, even though, the Ti−C peak has insignificant intensity and in narrow configuration.

Conversely, as can be seen in Figure 5I–N, the XPS narrow‐scan fitting of N 1s, C 1s, and Ti 2p analysis of these right‐/left‐handed chiral MXenes (P0.15D/P1L) identified dominant peaks, including O−N, O═C−N, C−Ti, C−N, C═O, −COO at the binding energies of ≈399, 401, 282/284, 286, 288, and 290 eV, respectively. In particular, the XPS N 1s comparisons of these chiral MXene colloids compared to pristine Ti_3_C_2_T_x_ nanosheets show a notable difference. The appearance of O═C−N peak in the narrow‐scan analysis of these chirality‐introduced colloidal heterostructures suggests a successful amidation reaction. This peak is absent in the XPS of the control sample, and is indicative of the formation of amide bonds between the amino groups of the used D‐/L‐cysteine amino acid and the oxygen‐containing functional groups on the MXene surface. This chemical modification enhances the material's functional diversity, potentially improving its stability and reactivity by introducing more robust nitrogen‐based functional surface terminations.

Furthermore, the C 1s fitting analysis of these representative chiral MXene colloids shows a peak broadening associated with the applied amino acid treatment. In particular, in the C 1s spectra of these amino acid‐treated samples, the peak at ≈286.5 eV becomes noticeably broader in both samples. This broadening signifies the addition of amine groups (−NH_2_), resulting from the reaction between the D‐/L‐cysteine amino acid and the MXene surface. The widening of the peak reflects the overlap of binding energies from newly introduced C−N bonds and existing C−O bonds. This modification indicates the effective attachment of amino groups, which potentially contribute to enhancing the surface chemistry of the pristine Ti_3_C_2_T_x_ MXene by making it more hydrophilic and facilitating further interactions with polar molecules. Adding amine functionalities can also potentially improve the material's chemical versatility, enhancing its aqueous durability and adsorption performance for colloidal applications that require reactive surface groups.

Additionally, our XPS Ti 2p narrow‐scan spectrum of these MXenes showed the identification of strengthened Ti−C bonds at almost similar binding energies that were detected for pristine Ti_3_C_2_T_x_ MXene prototype with higher peak intensity, suggesting their enhanced material stability against oxidation and over time oxidative degradation in water. In particular, the Ti 2p spectrum reveals that the Ti−C peak is significantly stronger in these amino acid‐treated samples, even after prolonged exposure to water (e.g., 60 days). This observation suggests that the amino acid forms stable bonds with the MXene surface (here Ti_3_C_2_T_x_), specifically through the amine group bonding with oxygen‐containing functional groups (e.g., Ti−O). This interaction likely passivates the surface, protecting the Ti−C bonds from hydrolysis and oxidation in aqueous media. The stabilization of the Ti−C bond through amino acid attachment indicates that the material's structure of these chiral MXene colloids becomes more chemically resilient, maintaining their structural integrity and preventing the typical degradation associated with long‐term water exposure. As pointed out, this improved surface stability is highly advantageous for the long‐term applications of MXene nanosheets, especially where they are subjected in aqueous environments.

Together, the presence of dominant carboxyl‐based termination alongside the emergence of amine‐containing peaks in the XPS data of these D‐/L‐chiral MXenes suggests the induced surface functionalization with carboxyl terminals and stable amine binding and chirality, highlighting the relatively higher stability of these MXenes colloids compared to the pristine Ti_3_C_2_T_x_ nanosheets (see Figure S8 and Table S3, Supporting Information). The presence of citric acid‐protonated the amine functional group of the D‐/L‐cysteine resulted in the presence of free radicals that can readily and effectively bond with the decorated terminal groups in the structure of functionalized Ti_3_C_2_T_x_ (e.g., −COOH: carboxyl, −COO⁻: carboxylate, C═O: carbonyl). The intensity of amine peaks in XPS analyses of the samples has been found to be comparable, suggesting effective surface enrichment of chiral MXenes with different levels of these functional groups based on the amino acid structure.

### 2.6 Surface Functional Groups Characterization of Chiral MXenes

Next, we performed Fourier‐transform infrared spectroscopy (FTIR) analysis to further study the surface functional groups and chemistry of Ti_3_C_2_T_x_ before and after carboxyl‐functionalization and chirality induction. In particular, the base characteristics of Ti_3_C_2_T_x_ MXene‐based materials, including pristine, carboxyl‐modified, and chiral carboxyl‐MXene colloids, were assessed by FTIR. As can be seen in Figure S9 (Supporting Information), our FTIR data of these MXene‐based samples show the identification of characteristic peaks related to typical Ti_3_C_2_T_x_’s surface functional groups, including hydroxyl (−OH) and carbonyl (C═O) terminals (that located at a depth of ≥10 nm XPS resolution) at the wavenumbers ranged from 400 to 4000 cm^−1^.

The FTIR spectra of carboxyl‐functionalized MXene nanosheets show the emergence of a minor peak at ≈1350 cm^−1^, which is assigned to the carboxyl group. In the FTIR analysis of these chirality‐induced samples, this peak was detected along with the emergence of new amine‐based terminals (i.e., N−H, C−H, N−H stretch, and NH_2_) at the wavenumbers at ≈1150, 1500, 3300–3500, and 3720 cm^−1^, suggesting the proper functionalization and modification of Ti_3_C_2_T_x_ MXene. Furthermore, a minor peak was identified at the wavenumber of ≈2540 to 2580 cm^−1^ in the FTIR spectra of these chirality‐introduced MXenes, which might be related to the S‐H stretching, thiol group of D‐ or L‐cysteine, and their attachments/interactions to the COOH‐functionalized MXene surfaces as a part of chiral surfaces or other organic compounds of these amino acids that contain sulfur atoms in their structures. No specific differences were observed that arose from the left‐ or right‐handed chirality of these materials.

Furthermore, Raman spectroscopy was used to study the structural characteristics, chemical bonding, intramolecular bonds, and surface functional groups of chiral MXenes. As shown in Figure S10 (Supporting Information), the modes of Ti_3_C_2_T_x_ MXene at shifts ranging from 200 to 700 cm^−1^ were identified in the Raman spectra of these samples, belonging to titanium and carbon vibrations. The Raman spectra of these representative left‐ or right‐handed chiral MXenes exhibit the low‐intensity modes at ≈148 cm^−1^ (*w₂*) and 720 cm^−1^ (*w₃*), which may correspond to A_1_ _g_ out‐of‐plane vibration of titanium and carbon atoms, respectively. The modes at ≈290 cm^−1^ (*w_5_*), 450 cm^−1^ (*w_5_*), and 550 cm^−1^ (*w_4_*) are likely associated with the E_g_ group vibrations, including the in‐plane shear modes titanium, carbon, and atoms of functional groups available on the surface of these MXenes. Taken all these together, our morphological and microstructural characterizations of these samples confirm the successful surface modification/chemistry tailoring of 2D Ti_3_C_2_T_x_ with bioactive chiral molecules to form stable mixed‐dimensional chiral MXene heterostructures.

### 2.7 Optical Surface Absorption Analysis and Long‐Term Colloidal Stability of Chiral MXenes

In next experiment, we studied the optical surface absorption properties and long‐term aqueous stability of these chiral MXene colloids after ≈60 days of the preparation process using UV/Vis spectroscopy. As shown in Figure S11 (Supporting Information), the optical absorption properties of these chiral MXene samples have been compared before and after subtracting from MilliQ water. Our UV–vis spectroscopy analysis of these materials suggests a competitive optical absorption arose from their transparent surfaces. In particular, the detection of characteristic carbon‐carbon (C−C) bonds and carbon‐titanium (C−Ti) bonds at the wavelengths ranging from ≈280 to 980 nm is observed, which can be assigned to the bonds related to carbon‐based compounds and surface functional groups available in the atomic structure of MXenes. These identifications highlight the longer‐term colloidal stability of these 2D‐0D/1D chiral Ti_3_C_2_T_x_ MXenes in aqueous media. In particular, these UV–vis analyses showed the presence of minor peaks, detected at the wavelengths of ≈280–400 nm (UV region), and also 400 and 700 nm and ≈900 nm, which likely corresponded to these MXene nanosheets and relative formation of Ti_3_C_2_T_x_ MXene quantum dots and surface titanium oxide nanoparticles during the applied treatment processes. In addition, the longer‐term enhancement in the aqueous stability of the prepared chiral MXene biomaterials was also evaluated by digital camera imaging of their dispersions for over two months of the preparation stage. As depicted in these UV–vis spectra, relatively good long‐term stability and dispersibility of these colloids were observed when the suspensions were stored at four degrees. Taking all these points into consideration, our UV–vis and other characterization data of these chiral materials suggest a typical characteristic of modified Ti_3_C_2_T_x_ MXene‐based flakes, which are enriched with abundances of stable bioactive surface functional groups for phyto‐applications.

### 2.8 Density Functional Theory (DFT) Calculations and Electrochemical Impedance Spectroscopy (EIS) Analysis of the Proposed Surface Interactions and Electronic Properties of Chiral MXenes

**Figure 6:**
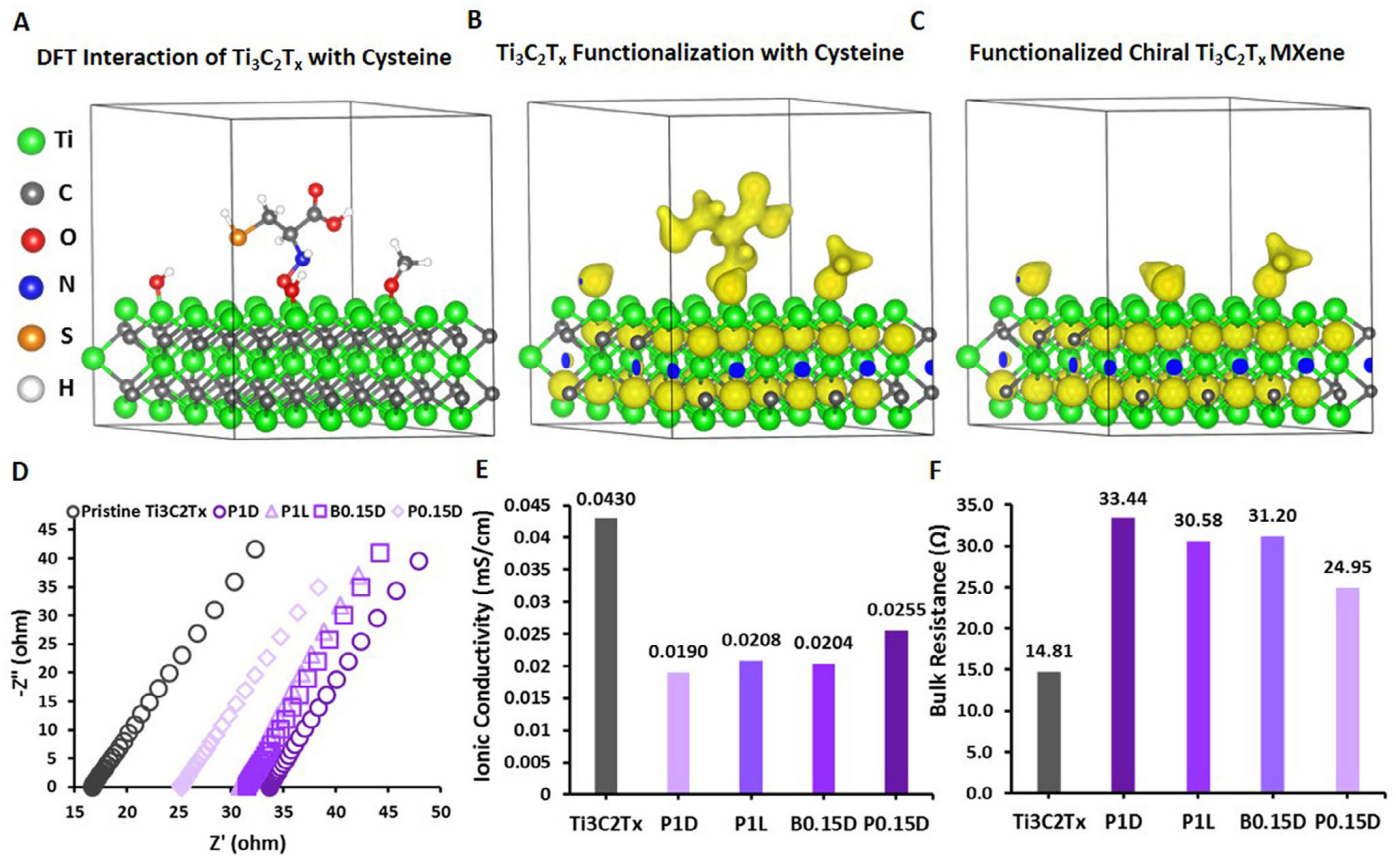
DFT calculations and EIS measurements of pristine Ti_3_C_2_T_x_ MXene nanosheets and chiral MXene heterostructures. A) Atomic structure of cysteine molecule adsorbed on the surface of functionalized MXene. Charge density distribution of B) cysteine adsorbed functionalized Ti_3_C_2_T_x_, and C, chiral functionalized Ti_3_C_2_T_x_. D–F) The electrochemical analysis of these samples, including Nyquist curves, ionic conductivity, and bulk resistance.

Next, to better understand the electronic properties of as‐functionalized Ti_3_C_2_T_x_ with cysteine ligands, we performed DFT calculations. Two different Ti_3_C_2_T_x_‐based structures were carried out, including one functionalized −OH and O═C groups and another one with cysteine molecules attached to the functionalized surface of this MXene. As shown in **Figure** **6**A–C, the total electronic charge density is distributed around the functional groups and carbon atoms. No charge density surrounded the titanium atoms in the MXene structure. Interestingly, when cysteine is bonded to the surface of this material, our calculations suggested that the majority of the charge is distributed around the cysteine molecule, providing insights into the nature of interaction at the interface of these chiral MXenes. Moreover, the charge density around cysteine suggests that it may be acting as an electron donor from the amine side and or acceptor from the carboxyl side, modifying the electronic structure and surface properties of pristine Ti_3_C_2_T_x_ MXene. Our surface interaction calculation analysis suggests the binding energy of the cysteine molecule on the surface of this amino acid‐functionalized MXene. The calculated binding energy relative to the total system energy is found to be −2652.4 eV. This negative binding energy suggests that a strong interaction between cysteine and MXene could occur, which contributes to enhancing the stability and oxidative degradation of the MXene‐cysteine interface. These DFT calculations are in good agreement with our characterizations and stability property measurements of these chiral MXene colloids, suggesting a successful chirality induction alongside the formation of highly stable heterostructures.

Moreover, we evaluated the electrochemical performance of these aqueous chiral MXene colloids and compared them with pristine Ti_3_C_2_T_x_ MXene dispersions through standard electrolyte‐containing battery cells. The panels D to E in Figure 6 depict the EIS, ionic conductivity, and bulk resistance of these samples. As can be seen, all the right‐ or left‐handed chiral MXene colloids show a higher bulk resistance compared to pristine Ti_3_C_2_T_x_. Additionally, our data from these samples show a remarkable reduction of ionic conductivity in all tested chiral MXenes when compared to the control MXene (downshifting from ≈0.043 to 0.019, 0.020, and 0.025 mS cm^−1^. The bulk resistance result of these materials suggests a considerable increase from 14.81 Ω in Ti_3_C_2_T_x_ to 33.44 Ω (maximum observed) in chiral MXene samples.

These experimental and computational analyses support the effective enhancement in the microstructure and properties of Ti_3_C_2_T_x_ MXene for the targeted bio‐applications. Noticeable decreases in ionic conductivity and simultaneously improved chemical stability in water can be attributed to several factors. First, amino acid attachment can neutralize surface charges, particularly deactivating negatively charged functional groups (−OH, −F, −O) that facilitate ion transport, thereby reducing the number of mobile charge carriers. Additionally, the bulkiness of amino acids introduces steric hindrance, obstructing ion transport pathways and limiting hydrated ion movement. *π*–*π* stacking interactions between these amino acids and these mixed dimensional MXene nanosheets/quantum dots colloids may further alter charge transfer efficiency.

Another contributing factor is the disruption of water adsorption and ion solvation. The introduction of amino acids can modify the hydrophilicity of these heterostructured colloids, potentially making them less effective at adsorbing water molecules and solvating ions, which further impedes conductivity. Moreover, amino acids can form an insulating layer on the colloid surface, altering electronic properties and reducing both electron and ion mobility. Changes in pH and electrostatic interactions may also play a role, as amino acids can alter the protonation state of surface groups, screen electrostatic interactions, and ultimately reduce ionic movement. Structural changes in these MXene colloid networks, such as aggregation or increased interlayer spacing, may further disrupt percolation pathways essential for efficient ion transport.

Despite the decrease in ionic conductivity, amino acid functionalization significantly enhances the chemical stability of these MXene colloids in water. This improvement is likely due to enhanced surface passivation, preventing oxidation or hydrolysis, and reduced dissolution or degradation, as amino acids stabilize surface groups against hydrolytic attack. Furthermore, functionalization improves colloidal stability, minimizing aggregation and maintaining dispersion in aqueous environments. Overall, while amino acid functionalization compromises ionic conductivity, it enhances the long‐term stability of these MXene colloids in aqueous media, making them more suitable for applications requiring prolonged aqueous stability.

### 2.9 Biocompatibility of Chiral MXenes with Arabidopsis Thaliana's Seeds and their Impacts on Seeds Sprouting, and Seedlings Germination/Development

As described earlier in the introduction, the biocompatibility of given nanomaterials, including novel chiral MXenes is among the key properties and is essential for their practical applications in agriculture. Indeed, an initial stage revolves around the biocompatibility of the treated materials with the seeds and their direct interactions/impacts on seed coating and seed‐to‐seedling transition, seedling growth, and maturation. Thus, in the first bioassay, we assessed the phyto‐compatibility and interaction of the chiral MXene aqueous colloids to ensure that treating with these biomaterials does not impose any significant adverse effects on the natural developmental processes of the seedlings at different concentrations and time points. To the best of our knowledge, this result is the first evidence on the biocompatibility and phyto‐interaction of chiral MXenes with seedlings.

**Figure 7:**
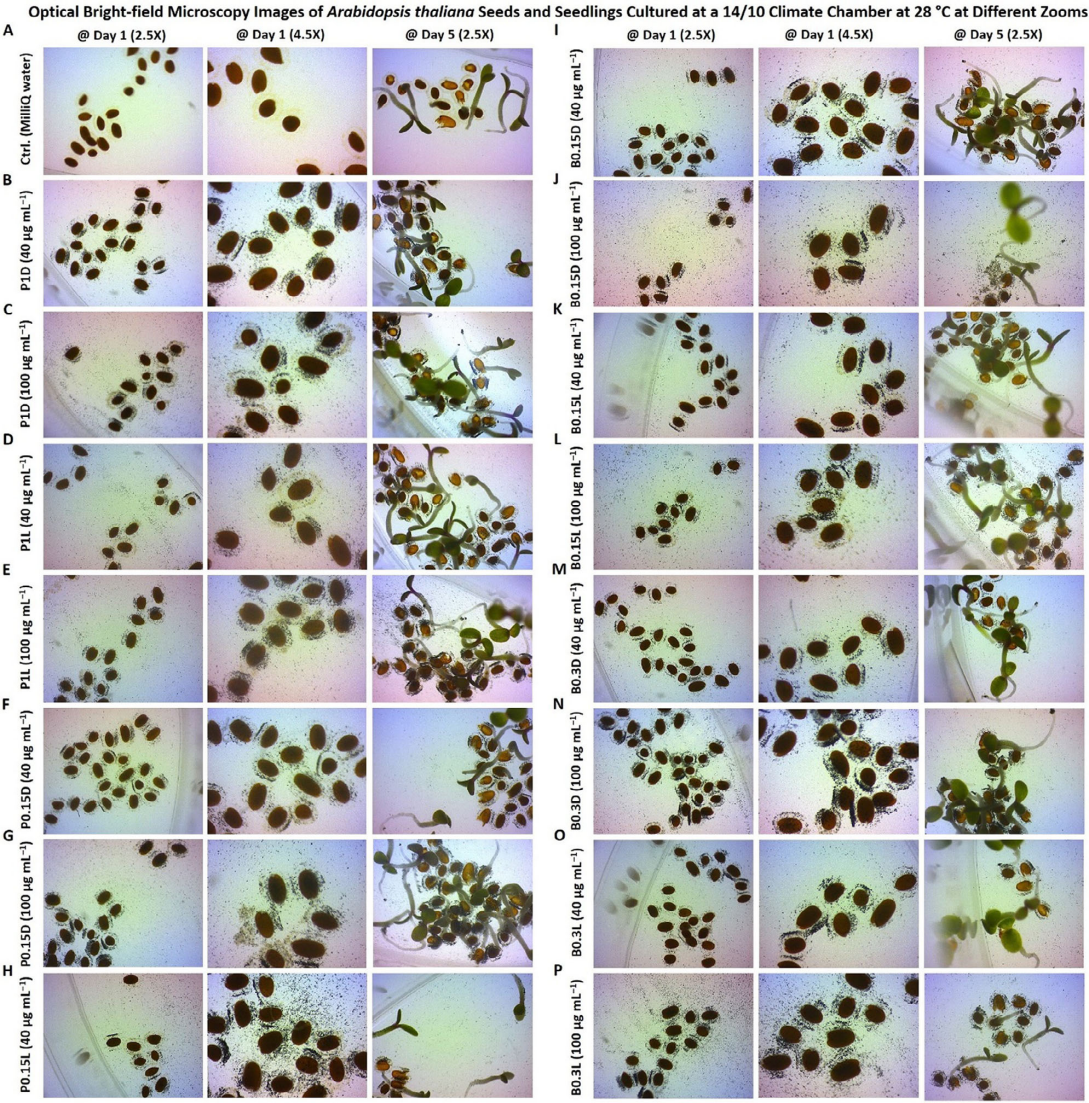
Assessment of phytocompatibility of these chiral MXene aqueous colloids with *Arabidopsis thaliana* Col‐0 seed coating, seed sprouting and germination, and seedling maturation. Optical microscopy depicted their seed‐to‐seedling transition process at different time points (up to day‐5 post‐treatment) of A) control (MilliQ water), B) P1D@40 µg mL^−1^, C) P1D@100 µg mL^−1^, D) P1L@40 µg mL^−1^, E) P1L@100 µg mL^−1^, F) P0.15D@40 µg mL^−1^, G) P0.15D@100 µg mL^−1^ H) P0.15L@40 µg mL^−1^, I, P0.15L@100 µg mL^−1^, J) B0.15D@40 µg mL^−1^, K) B0.15D@100 µg mL^−1^, L) B0.15L@40 µg mL^−1^, M) B0.3D@100 µg mL^−1^, N) B0.3D@100 µg mL^−1^, O) B0.3L@40 µg mL^−1^, and P) B0.3L@100 µg mL^−1^ in a sucrose‐supplemented MS media (*n* = 10–20 per group). These representative bright‐field images showed that the materials at both tested doses were readily and spontaneously localized/neighbored around the seeds of *Arabidopsis thaliana* in a dose‐dependent fashion. Our qualitative microscopic observation suggest no significant visible adverse effects in the transformation of *Arabidopsis thaliana* seeds; rather these chiral MXenes have contributed to enhancing their overall germination, sprouting, root/shoot development, and seedlings maturation.

In particular, the biocompatibility and direct interaction of the prepared chiral MXene colloids on the *Arabidopsis thaliana *Columbia‐0 (Col‐0) seeds were assessed. As shown in **Figure** **7**A–P, treating the seeds with these materials effectively resulted in a spontaneous seed coating upon the initial hours of incubating with sucrose‐supplemented Murashige and Skoog (MS) media in the climate chamber. As shown, treating with these colloids at tested concentrations (40 and 100 µg mL^−1^) has been found localized in close proximity to these seeds in a dose‐dependent manner. These biocompatibility assessments, as well as the relative bioactivity effect of these chiral MXene colloids with Col‐0 *Arabidopsis thaliana* seeds, showed high levels of phyto‐compatibility within the first five days of treatment. These qualitative data suggest the intrinsic biocompatibility of these chiral MXene aqueous colloids without imposing any significant adverse effects on seed sprouting or seedling germination and maturation of *Arabidopsis thaliana*. We also evaluated the longer‐term biocompatibility of these MXenes by monitoring the seedlings’ development at different time points of day 1, 5, and 10 post‐treatment compared to control groups. Our optical bright‐field microscopic assessments of these seedlings suggest good biocompatibility of these colloids at tested concentrations. As shown in Figure S12 (Supporting Information), the applied treatment with these chiral MXene colloids has effectively promoted the seed‐to‐seedling transition and overall growth within the first ten days of the culture. This spontaneous biostimulant activity of chiral MXenes in *Arabidopsis thaliana* seedlings is likely due to their intrinsic surface properties and biomaterial characteristics to be readily and effectively localized around these seeds and likely induce functional bioactivity responses.

### 2.10 Plant Stomatal Closure‐Inducing Bioactivity and Underlying Mechanisms of Chiral MXenes

It is well understood that upon exposure to an elicitor‐active agents, whether it is recognized initially as a phytotoxic substance, invasive organism, or bioactive compound/stimulant, the plants perceive them intrinsically as stressors at initial interactional stages. The plant immune system and corresponding defense responses subsequently activate case‐related bio‐immunological mechanisms to specifically interact with them and counter these stressors for survival, ensuring a balance between defense, growth, and development. In the particular case of exposure of the plants to harsh abiotic stress, either temporary or sustained conditions, it is crucial to respond immediately for effective resilience and adaptation. This natural plant’ adjustment can effectively survive them without causing significant adverse impacts on their yields and productivity if the abiotic stress conditions are recognized as mild or intermittent. However, in the case of farming regions with severe climate changes or extreme environmental conditions, boosting the plants’ tolerance and fertilization using phyto‐stimulants is essential.

One of the defense‐related mechanisms is triggering the production of reactive oxygen species (ROS). This systemic ROS generation is an initial hallmark of plant immune responses against various categories of stressors, including abiotic drought and salinity conditions, as well as upon continuous exposure to light stress. In this context, several studies have been reported on the impact of bioactive materials on inducing immediate or sustained ROS production in plants and regulating the associated genes for enhanced defense responses to stress. Among them, significant attention has been given to the strategy of using highly biocompatible nanomaterials to enhance the plant's systemic tolerance to abiotic stresses with the aim of reducing the consumption of agrochemicals and improving the environmental changes. The large surface area of bioactive materials supports the enhanced capacity to interact with plants at lower working doses, compared to the conventional materials or agrochemical product types, which is likely eco‐friendly and cost‐effective, if such materials prove sufficient safety for agricultural applications. Therefore, the remaining challenge revolves around designing and synthesizing highly efficient and biocompatible stimulant materials to reduce the safety risks associated with the health of plants, soil, and surrounding environments.

In the context of plant defense mechanisms, stomatal closure is one of the pivotal biological responses, which is activated in leaf guard cells as a prompt immune response upon identifying the stress of contact with foreign substances or invaders. This plant's innate mechanism is somehow correlated with inducing ROS production, especially hydrogen peroxide (H_2_O_2_), and underlying signaling pathways. The stomatal closure‐inducing mechanism can be activated within minutes of stress perception in plants for survival as a first line of defense against stressors, including abiotic drought and severe salinity conditions, to reduce excessive water loss, control homeostasis, and maintain osmotic balance.^[^ ^92^, ^93^, ^94^ ^]^ It is known that abscisic acid (ABA) is one of the key underlying plant hormone‐based mechanisms behind the stomatal closure response, which is cross‐linked with the induction of ROS generation in plant leaf guard cells through activating specific calcium ion channels, which subsequently cause the changes in ion flux and depolarization of the cell membrane, and ultimately triggering the stomatal closure as a layer of protection within defense responses.^[^ ^93^, ^95^ ^]^ In addition, the stomatal closure crossovers with the absorption of carbon dioxide (CO_2_) by plants can impact the photosynthesis process, prompting their growth, developmental stages, and productivity.^[^ ^96^, ^97^, ^98^, ^99^ ^]^

**Figure 8:**
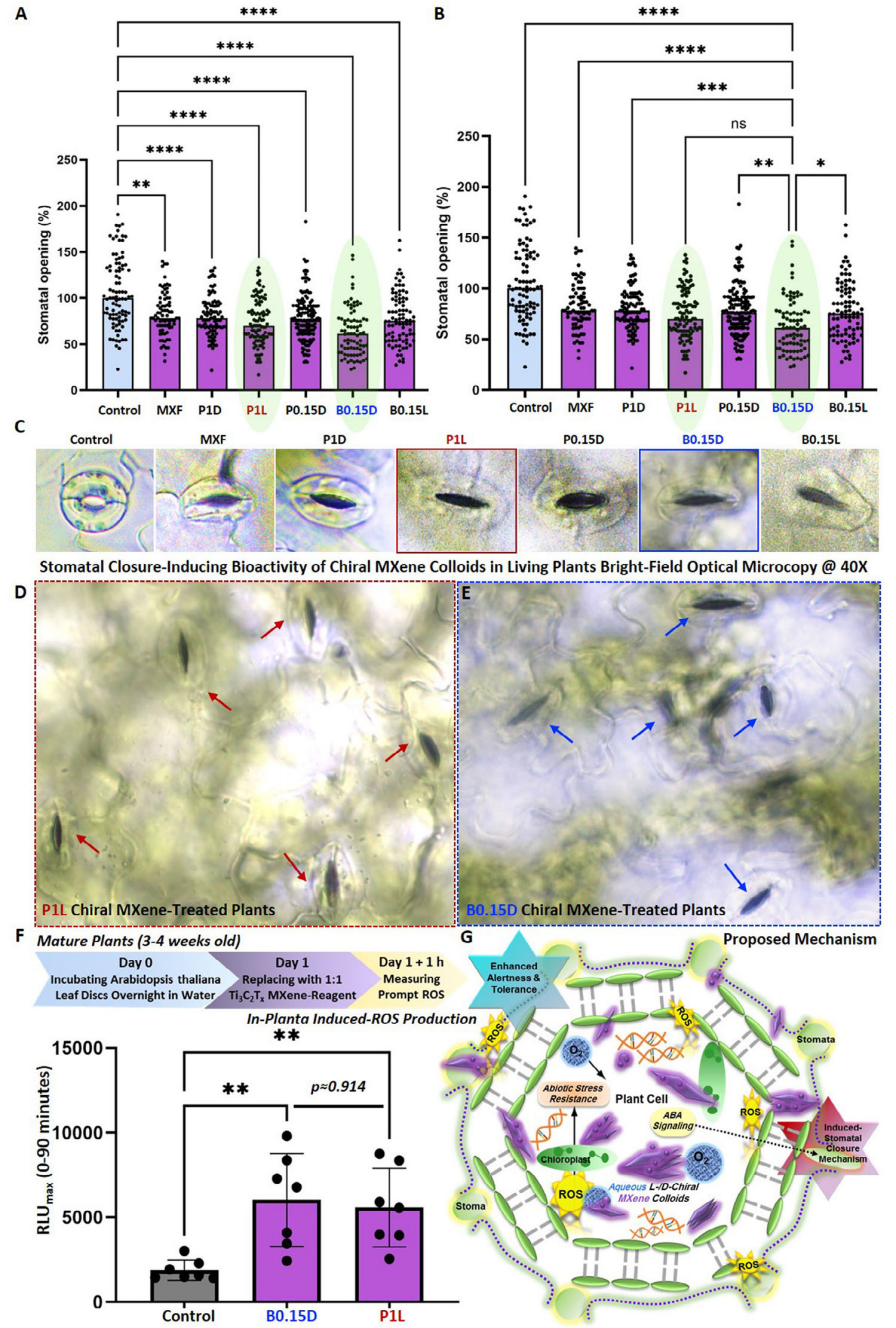
Stomatal closure‐inducing bioactivity and induced ROS‐production mechanism of chiral MXene colloids. Pristine Ti_3_C_2_T_x_ MXene and chiral MXene‐based aqueous colloids were foliar‐sprayed at the concentration of 10 µg mL^−1^ on *Arabidopsis thaliana* compared to water foliar‐sprayed plants in the control groups. A–E) The *in‐planta* stomatal closure responses induced by these samples and their representative optical microscopy images of these experimental and control samples depicting the intrinsic impact of Ti_3_C_2_T_x_ MXene before and after functionalization and chiral modification on significantly enhancing the stress‐related stomatal closure mechanisms in these optimal chiral MXene aqueous colloids‐treated plants compared to the control plants (*n* = 70 to 140 per group). The stomatal closure‐inducing bioactivity impact and representative bright‐field imaging of this sample at a low working dose depict its significant impact on inducing this defense‐related mechanism compared to the control group. Our statistical analysis of the plants treated with chiral MXene aqueous colloids showed no significant difference between the stomatal closure‐inducing bioactivity of the B0.15D and P1L, suggesting highly competitive bioactivity of these samples treated with the D‐ or L‐cysteine. F,G) Experimental timeline and ROS‐induced eliciting response of these colloids in comparison with control (*n* = 7 per group, B0.15D vs control: p≈0.003 and P1L vs control: p≈0.007) and the proposed schematic illustration of the underlying bioactivity mechanisms. Our data suggest that optimal chiral MXenes colloids could effectively induce ROS‐related bioactivity mechanisms in *Arabidopsis thaliana* upon early interaction (e.g., 1–2 h).

In the next bioassay, therefore, we wanted to examine the intrinsic bioactivity impact of chiral MXenes at a relatively low concentration of 40 µg mL^−1^ on inducing the stomatal closure response in model *Arabidopsis thaliana* plants. As shown in **Figure** **8**A–E, our assessment of the direct *in‐planta* interaction of these colloids with *Arabidopsis thaliana* suggests their significant and intrinsic bioactivity in reducing the stomata apertures in the tested model plants compared to the control group. This data revealed that the average stomata aperture sizes of the plants foliar‐sprayed with a small volume of these chiral MXene aqueous colloids (≈1 mL) were significantly reduced compared to the pure water‐treated *Arabidopsis thaliana* in the control group (n, is equal to 70–140 per group isolated from five individual plants, *p* < 0.05). Additionally, the comparison of the bioactivity of these MXene‐based biomaterials showed that all of the tested samples could effectively induce the stomatal course in these plants. However, our bio‐statistical analysis of these samples suggested higher bioactivity of the B0.15D and P1L samples, with a slight superiority found in the plants treated with B0.15D colloids. This data highlights the intrinsic bioactivity of these optimal right‐ or left‐handed chiral MXene colloids for inducing stomatal closure mechanisms in plants. Notably, as discussed in the previous section, this stomatal closure action is correlated with ROS generation by the impact of elicitor‐active biomaterials.

To further understand the related mechanism behind this bioactivity, we evaluated the eliciting activity of optimal chiral MXenes colloids with a focus on measuring ROS production as an early alertness and defense mechanism upon stress detection in *Arabidopsis thaliana* plants. As shown in Figure 8F,G, Our data suggest adding the chiral MXene aqueous colloids at 100 µg mL^−1^ to the *Arabidopsis* leaf disks could readily and effectively enhance the ROS production as an underlying mechanism of stomatal closure in as‐treated plants upon early stages of interaction with these elicitor‐active biomaterials (e.g., 1–2 h). This prompt ROS generation is found to occur at early responses, as the maximum relative light units (RLU_max_) were detected in these plant leaf disks within the first 30 min. In particular, this eliciting data show significantly increased oxidative burst in the presence of B0.15D or P1L colloids compared to the control plants. These assessments suggest that both left‐handed and right‐handed chiral MXenes have the intrinsic capability of inducing eliciting responses in plants, enhancing their alertness and resistance to stress conditions. As can be seen in this panel, the statistical analysis comparison of these chiral MXenes suggests a slightly higher eliciting activity of B0.15D, which is in agreement with the stomatal closure responses of these chiral MXene colloids. Hence, we continued the next *in‐planta* experiments with control and the optimal chiral MXene (B0.15D) to reduce the sample size in the rest of the performed bioassays.

Next, we wanted to assess whether the applied foliar treatment did not impose any significant adverse effects on the overall morphology or natural physiological condition of treated *Arabidopsis thaliana* plants with this optimal chiral MXene material. As can be seen in Figure S13 (Supporting Information), our assessment of optical microscopy of the isolated cells from the shoot parts of these plants suggests no particular associated changes at cellular and tissue levels, highlighting the phyto‐compatibility of this material after direct interaction with mature *Arabidopsis thaliana* plants at the tested concentration.

### 2.11 Short‐/Mid‐Term Biostimulant Effect of Chiral MXenes on Arabidopsis Thaliana Seedlings

**Figure 9:**
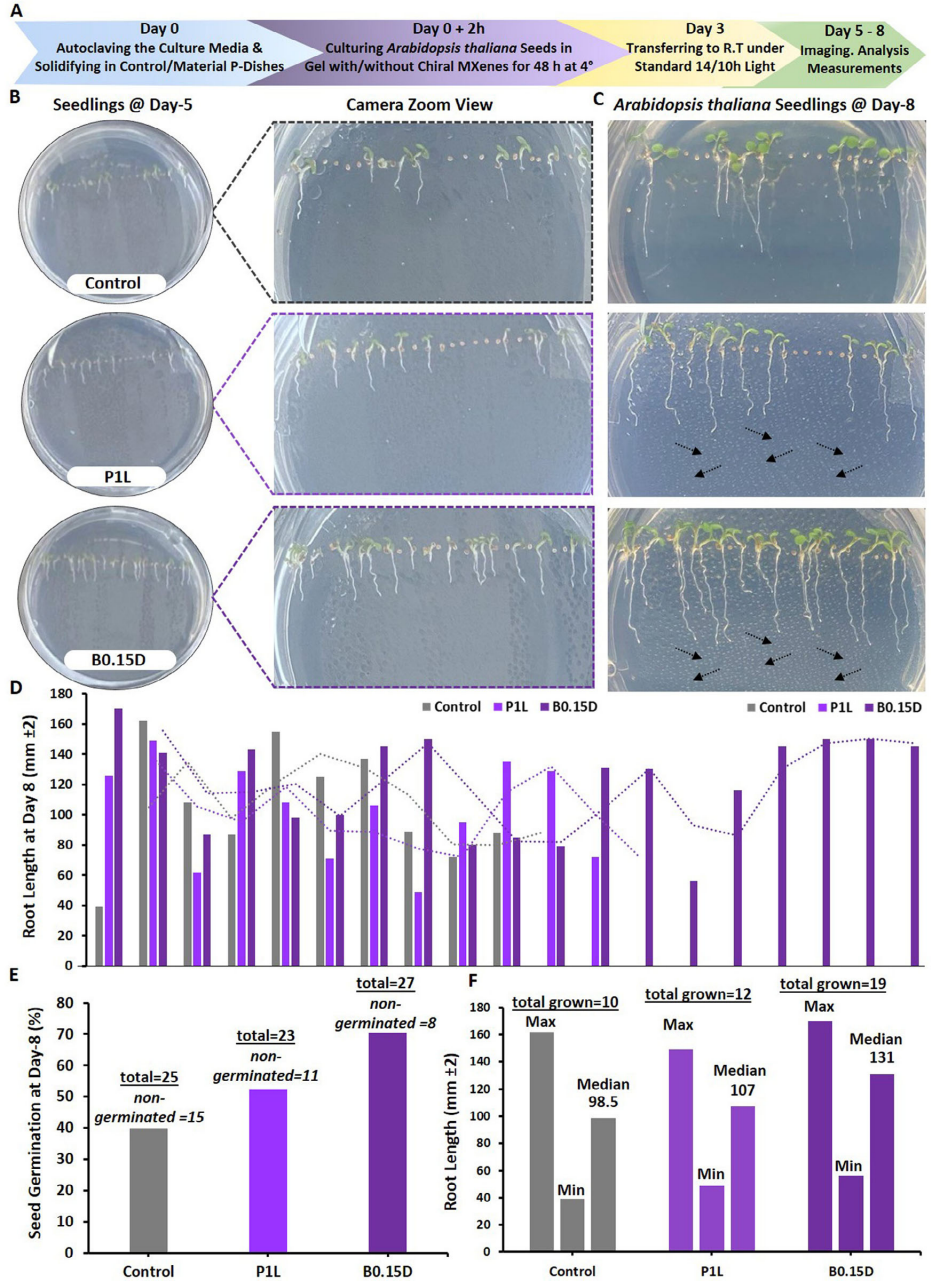
Illustration of the experimental timeline and biostimulant activity of chiral MXenes at different short‐ to mid‐term time points. A–F) The seeds of *Arabidopsis thaliana* Col‐0 were line‐cultured in solidified media with and without optimal chiral MXene colloids at a concentration of 88 µg mL^−1^. The camera images and their related seed germination and root length analysis showed a remarkable enhancement in the maturation of the chiral MXene‐treated seedlings at day 8 post‐treatment. Moving average trendlines were obtained by Excel software and used to compare forecast models for experimental and control groups. The ImageJ was used to measure the camera images of the roots using a similar trend. The measured max, min, and median of the roots also suggest the bioactivity of both tested chiral MXenes with a slight superiority of B0.15D colloids (n: is equal to at least twenty‐five per sample).

In the next experiment, we evaluated the bioactivity effect of optimal chiral MXene colloids on enhancing the seed germination and seedling sprouting and maturation of *Arabidopsis thaliana*. In particular, their bioactivity effects were assessed qualitatively and qualitatively for up to 8 days post‐treatment. As shown in **Figure** **9**, the seedlings’ germination, maturation, and root elongation were compared with the control group. Our data show a notable improvement in the transition of these seeds to mature seedlings in both P1L and B0.15D samples compared to the control group with a slight superiority of B0.15D collides. The analysis of these samples depicts that the treated seedlings with chiral MXene colloids could germinate better alongside promoted root development and overall maturation compared to control groups. The moving average trendlines of the samples revealed an enhanced root growth in B0.15D. These superior biostimulant properties were compared with this chitosan 661‐Cl substance at the same dose (see Figure S14, Supporting Information).

### 2.12 Long‐Term Biocompatibility of Optimal Chiral MXenes with Arabidopsis Thaliana Plants

**Figure 10:**
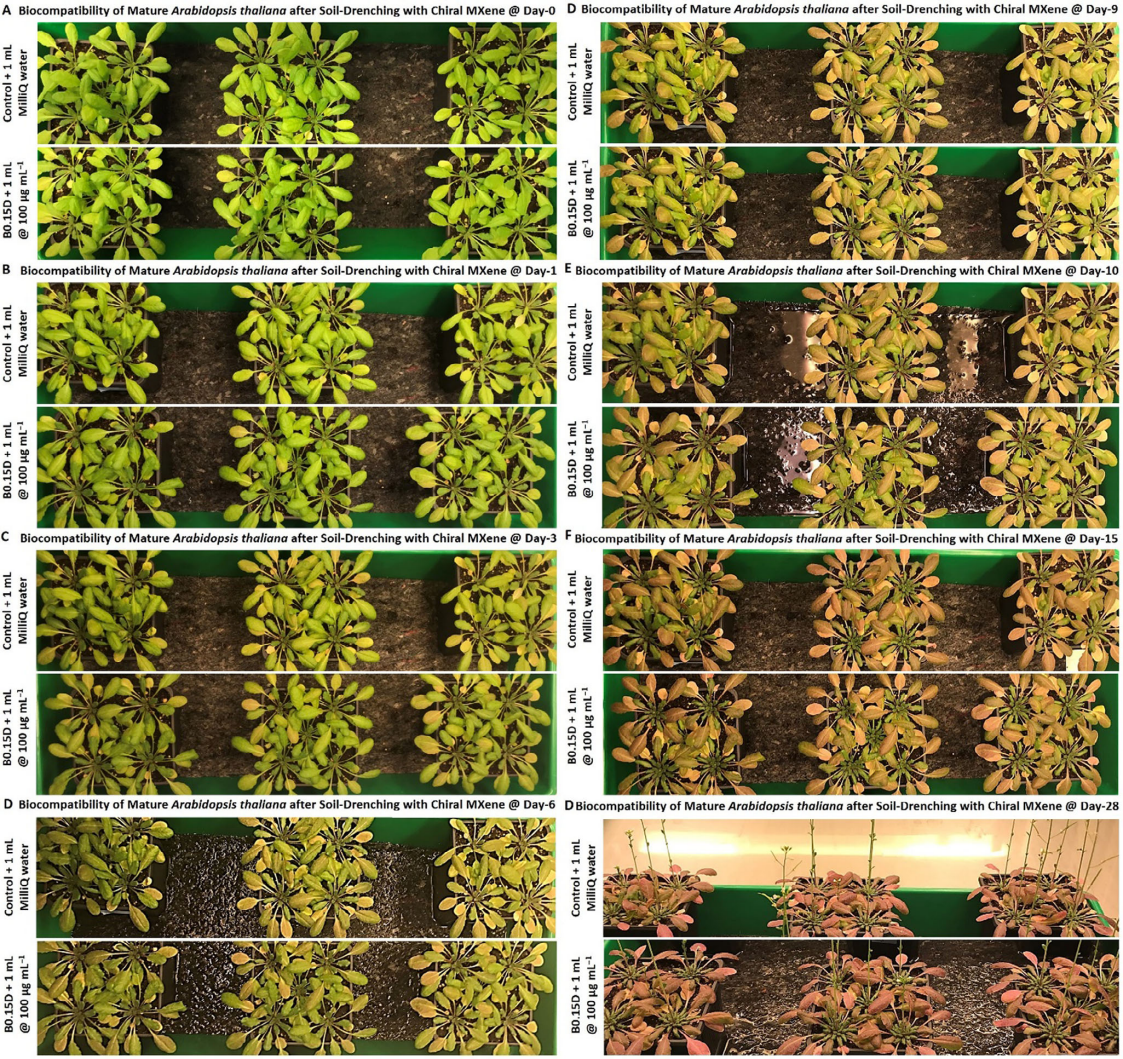
*In‐planta* qualitative short‐, mid‐, and long‐term biocompatibility of the B0.15D aqueous collides. Assessment of biocompatibility and direct interaction of these colloids at the concentration of 100 µg mL^−1^ with young and mature *Arabidopsis thaliana* (3–4 weeks old) and their qualitative impacts on these plants’ overall growth, and physiological conditions at different time points of day‐0, 1, 3, 6 9, 10, 15, and 28 post‐soil‐drenching treatment applied on the entire leaf/shoot parts (*n* = 3 and each pots represents five individual *Arabidopsis thaliana* plants). Our longer‐term phyto‐compatibility assessments of these colloids showed no significant adverse effects of the plant's natural growth, aging, flowering, and overall visible conditions compared to control plants.

Next, the long‐term phytocompatibility of the B0.15D sample has been assessed at different time points. In particular, we evaluated the direct and longer *in‐planta* effect of these chiral MXene aqueous colloids (≈10 mL) on the soil‐drenched *Arabidopsis thaliana*, where the plants were grown, developed, and maintained under the standard climate chamber conditions for up to around one month (28 days post‐treatment). Besides, the natural aging of the mature *Arabidopsis thaliana* (around three weeks old) was observed at two different standard conditions of the climate chamber and greenhouse to distinguish between the natural growth of the plant and natural aging from any probable adverse phyto‐effects caused by the material treatment or interaction. As can be seen in **Figure** **10** and Figures S15 and S16 (Supporting Information), our data qualitatively showed no significant changes or adverse effects in the overall plants’ growth or physiological functions compared to the control plant treated with pure water. Notably, the process of natural aging in these experimental and control groups has been observed to be normal in both groups, suggesting a high level of biocompatibility of the B0.15D colloids at the tested time points. Furthermore, Figure S17 (Supporting Information) illustrate the normal aging process of the *Arabidopsis thaliana* plants without any treatment from around week 3 to week 7, where the plants have been standardly watered with a similar trend were initially grown. These data suggest that at normal growing conditions in the climate chamber, no eye‐visible differences and significant adverse effects can be observed between the natural growing/aging process of these plants before and after soil‐drenching with/without these chiral MXene aqueous colloids in terms of growth, leaf's morphology and color, as well as the flowering cycles, which naturally occurs at the approximate age of two months.

Moreover, we wanted to examine the long‐term of the B0.15D biomaterial in seedlings and to ensure that culturing the same seeds of Arabidopsis thaliana plants with these chiral MXene colloids does not adversely impact the natural processes of seed‐to‐seedling transition, including seed coating, seed sprouting and germination, as well as the maturation of their seedlings inside the climate chamber. As shown in Figure S18 (Supporting Information), the digital camera imaging of these seeds and the monitoring of their maturation for up to day 14 post‐treatment depict that the designed seed‐coating and materials localization treatment that was occurred spontaneously by directly adding the colloids into the MS media and without any enhanced uptake techniques, could readily and effectively improve the seed sprouting and germination in the model tested. By comparing the seeding maturation in the control and B0.15D groups, a slight improvement can be observed in the germination quality/ratio of these seeds, which is most likely attributed to the bioactivity of this chiral MXene biomaterial. Taken together, these in‐vivo assessments support the high long‐term phyto‐compatibility of these colloids, which is essential for their practical applications in nano‐agriculture.

### 2.13 *In‐Planta* Drought Resistance‐Inducing Bioactivity of Optimal Chiral MXenes

In the next bioassay, we examined the intrinsic capability of these colloids to enhance the plant's tolerance against continuous drought stress to simulate the extreme environmental drought conditions. In particular, the regular watering of mature *Arabidopsis thaliana* plants (3–4 weeks old) in both control and experimental groups has been stopped after soil‐drenching with ≈10 mL of pure water and the B0.15D aqueous colloids at the concentration of 100 µg mL^−1^, respectively, to impose continues severe drought stress. To do so, we monitored the natural growth, aging, and physiological conditions of these plants at two different standard greenhouse and climate chamber models. These drought conditions have been continued until significant dryness symptoms appeared until the plants experienced visible damage, and their leaves started to destroy due to the imposed prolonged dryness condition. This cycle usually takes around three weeks in mature *Arabidopsis thaliana* plants following the cessation of watering. Thus, upon visualization of substantial shoot parts’ damage, the plants have been re‐watered with a sufficient amount of water one or two times, and their recovery and overall conditions, including the natural flowering process and systemic development, were carefully observed by digital imaging at different time points.

**Figure 11:**
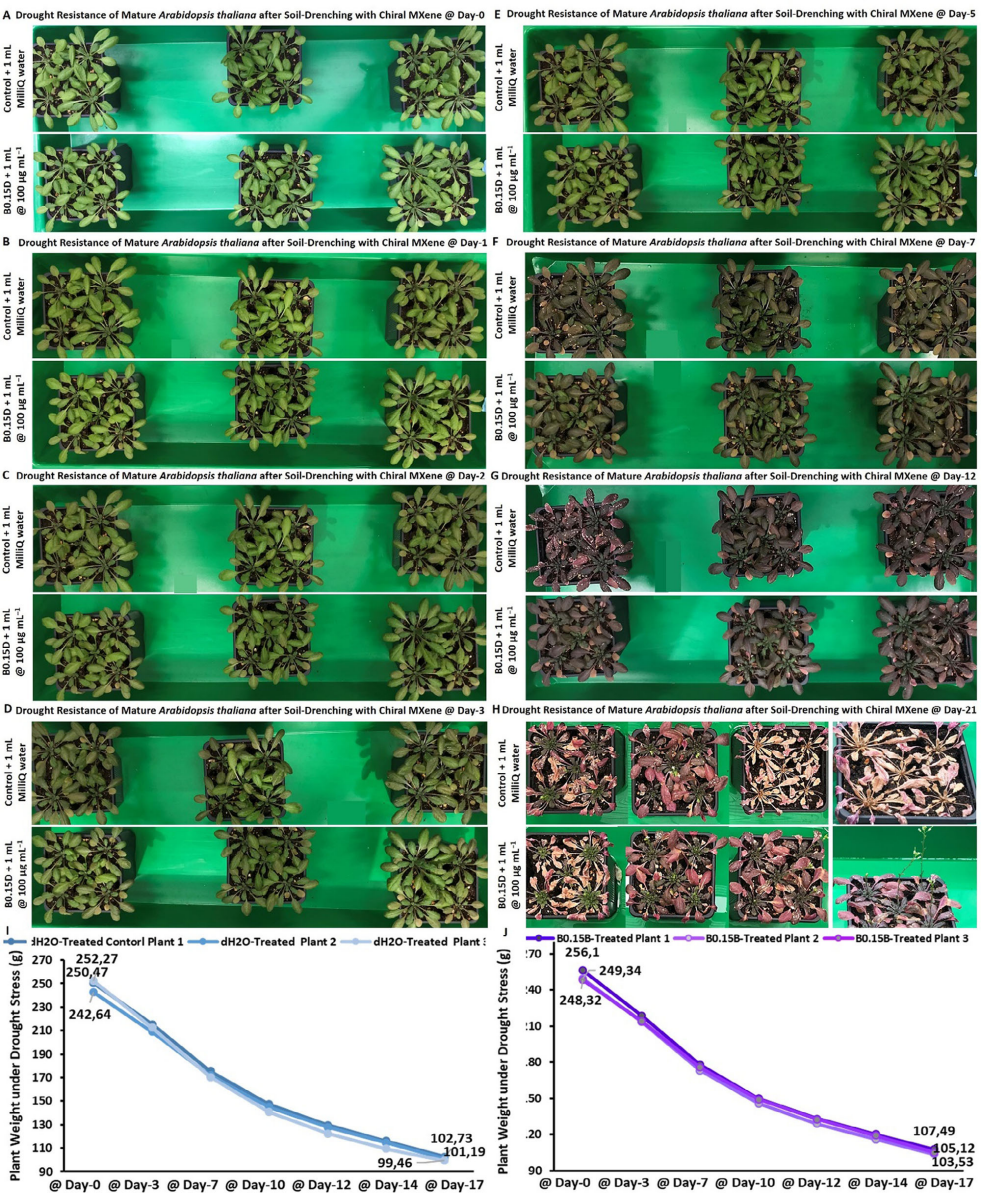
*In‐planta* short–, mid‐, long‐term drought‐resistance bioactivity properties and weight analysis before and after continuous exposure to drought stress condition inside greenhouse in *Arabidopsis thaliana* treated with B0.15D chiral MXene aqueous collides. A–H) Assessment of the plant's tolerance to continues extreme abiotic drought stress conditions for up to around three weeks post soil‐drenching treatment inside a standard greenhouse. Interaction of these chiral MXene colloids at the concentration of 100 µg mL^−1^ with young and mature *Arabidopsis thaliana* plants (3–4 weeks old) was qualitatively monitored compared to control plants, and their impacts on these plants’ overall growth, flowering, and aging were observed by digital imaging. I,J) Assessment of the plant's weight and dehydration tolerance under extreme abiotic drought stress conditions at different time points for up to 17 days post soil‐drenching treatment in control and chiral MXene‐treated plants.

As shown in **Figure** **11**A–H, under the applied greenhouse drought condition, the plants in both control and B0.15D‐treated groups stayed relatively healthy for up to five days post‐treatment. Accordingly, our assessment of the overall plants’ health under this abiotic stressor revealed that noticeable damage to the shoot sections (leaf and stem) started to become visible as early as day 7. These symptoms have been found to reasonably increase over time, and the images of plants on day 12 of treatment depicted a higher level of damage intensity. In this stage, the soil‐drenched plants with these chiral MXene colloids showed a slightly higher tolerance against drought compared to water‐treated samples. The trend is further continued, and our observation suggests that upon re‐watering the plants, those treated only once with the B0.15D material showed an overall enhanced survival supported by the natural flowering process at day 21 post‐treatment. Notably, the quantitative assessment of these drought assays suggested that the weight of plants (including their pots’ weight) in the B0.15D‐treated samples was considerably higher than the plants of control groups when comparing the initial and final weight of individual plants in each experimental and control groups at different time points tested (day‐0, 3, 7, 10, 12, 14, and 17).

As shown in the panel I and J of Figure 11, the summation of total weight values of the whole potted plants in the water‐treated control group (*n* = 3, and each pot contained five individual plants) on day 0 of soil‐drenching was 745.38 grams. This weight in these plants is decreased to 303.38 grams at the day‐ 17 post‐soil‐drenching because of the applied continuous drought conditions. This results in a total approximate weight loss of ≈442 grams across these plants, showing a significant reflection in plant biomass due to the imposed prolonged abiotic drought stress. In the case of the plants treated with the B0.15D chiral MXene colloids, our measurements suggested that the initial total weight of the similar population/age of plants at day 0 was 753.76 grams; by day 17 post‐treatment, the final weight of these experimental plants was reduced to 316.14 grams, indicating a weight loss of ≈437 grams. These changes in the weight of stressed plants, affected by water loss and plant slowdown in metabolic processes, suggest a relatively higher tolerance to drought in the treated samples with these chiral MXene colloids.

Besides, it is speculated that this bioactivity refers to the interaction of chiral MXenes with soil‐drenched plants, enhancing drought resistance mechanisms. In particular, nano‐stimulatory materials enriched with bioactive surface termination such as –COOH, −OH, and amine functional groups can feasibly form hydrogen bonds with the water's molecules, improving their holding capacity in the soil and improving the plant tolerance against fast de‐hydration. In addition, these functional groups, especially amine‐based terminations, are likely to enhance the production of proline or other osmolytes in plants, supporting them to keep hydrated for more elongated durations and maintain osmotic balance during harsh drought stress conditions. This mechanistic hypothesis is highly beneficial for the plant biostimulation of chiral MXenes.

**Figure 12:**
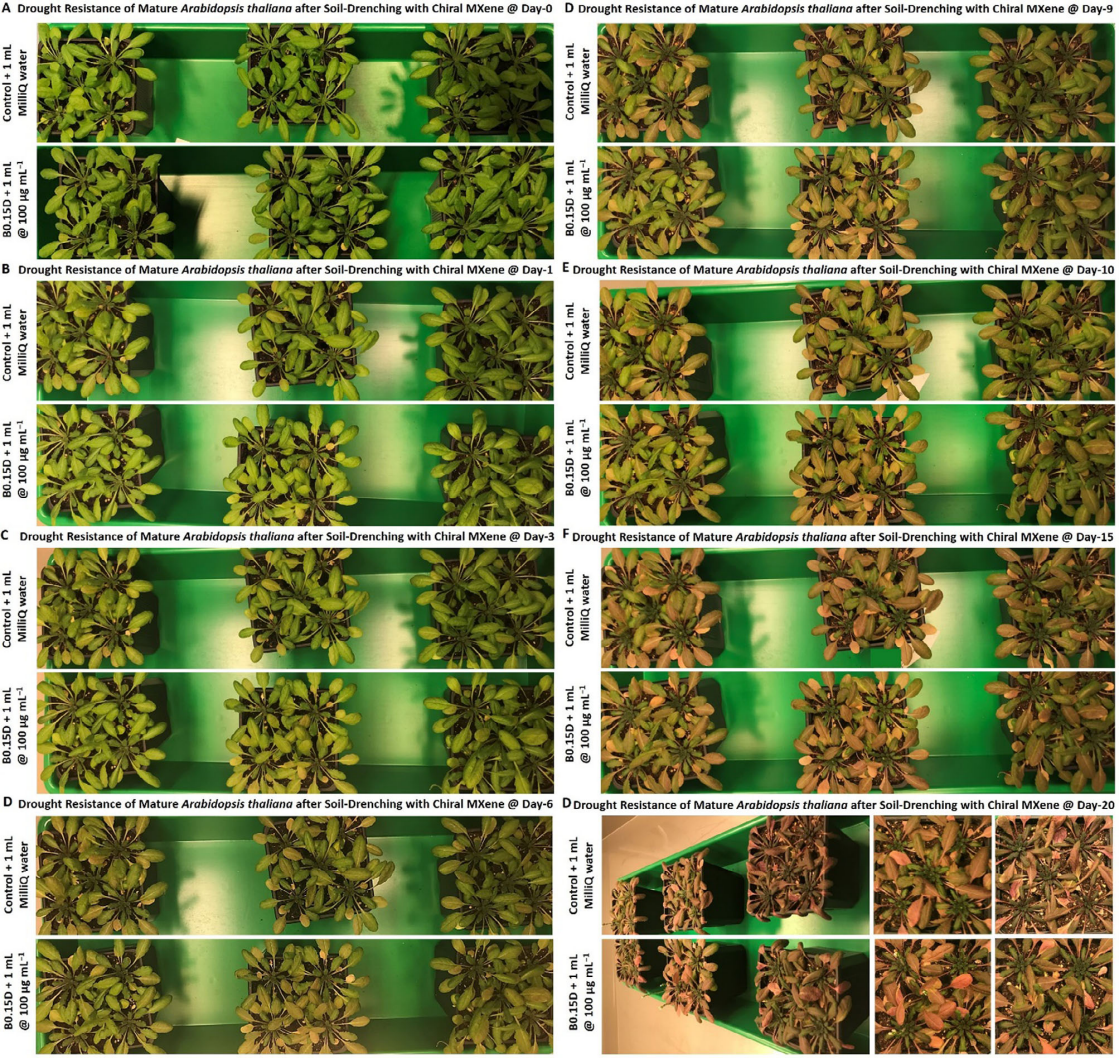
*In‐planta* short‐, mid‐, and long‐term drought‐resistance bioactivity impact of the B0.15D aqueous collides in *Arabidopsis thaliana* inside climate chamber. Assessment of the plant's tolerance to continues extreme abiotic drought stress conditions for up to around three weeks post soil‐drenching treatment and maintaining the plants under the standard climate chamber conditions. Interaction of these chiral MXene colloids at the concentration of 100 µg mL^−1^ with mature *Arabidopsis thaliana* (3–4 weeks old) was qualitatively monitored compared to control plants, and their impacts on these plants’ overall growth, flowering, and aging were observed by digital imaging.

We also assessed this enhanced plant resistance in the ideal condition of the climate chamber. As can be seen in **Figure** **12** and Figure S19 (Supporting Information), the plants showed resistance to drought. Within the first week post‐treatment likely due to the presence of higher humidity inside the climate chamber within the performed bioassay. In particular, the plants in both the control and experimental groups started to show drought symbols in the second week of soil‐drenching treatment. It is important to point out that these damages could be partially referred to as the natural effect of natural aging, as no significant difference was observed within the plants in both groups. In addition, our observations suggest a slight enhancement in B0.15D‐treated plants compared to the control groups at day 20. These images showed that the chiral MXene collides could effectively enhance the tolerance of *Arabidopsis thaliana* with more development of systemic leaves and their flowering cycles. These qualitative assessments after four weeks of soil‐drenching further confirm the appearance of fewer damage symptoms in the experimental plants after re‐watering. Together, these data on drought‐resistance‐inducing bioactivity of the B0.15D collides in greenhouse and climate chamber tested modes confirmed the enhanced plant's tolerance against drought conditions. Furthermore, these findings are aligned well with our stomatal closures‐inducing assessment and the superior bioactivity of these chiral MXene colloid in tested plant models, supporting their multifunctional phyto‐properties for boosting their alertness, response mechanisms, and tolerance against different abiotic stressors.

### 2.14 *In‐Planta* Salinity Resistance‐Inducing Bioactivity of Optimal Chiral MXenes

In the next bioassay, we evaluated the capability of these chiral MXene colloids to enhance the plant's tolerance against salinity stress after exposure to a relatively high dose of sodium chloride (NaCl, 200 mm) in water solution to simulate the agricultural regions with severe salinity conditions. In particular, the regular watering of mature *Arabidopsis thaliana* plants (3–4 weeks old) in both control and experimental groups has been stopped after soil‐drenching with ≈10 mL of pure water, and these chiral B0.15D aqueous colloids at the concentration of 100 µg mL^−1^, respectively. To do so, we monitored the natural growth, aging, and overall physiological condition of these plants at two different standard greenhouse and climate chamber models of salinity stress. These abiotic stress conditions have been continued for up to around one month after treatment (a total of ≈7–8 weeks) and until significant symptoms of salinity stress appeared, and the plants experienced eye‐visible damages due to the imposed prolonged condition and beyond the normal aging process. During these experiments, the plants received the same volume/concentration of NaCl‐water at different intervals like usual watering (every 2–3 days). The imposed cycle of osmotic and or ion‐based phytotoxicity was appearing significant salinity symptoms in the tested plants, including leaf chlorosis and excessive yellowing likely through the accumulation of sodium ions (Na⁺) and subsequent abnormal degradation of chlorophyll, as well as slight leaf necrosis (leaf tissue death) at the edges and turning into brown color due to the imposed ion toxicity of salinity stressor.

**Figure 13:**
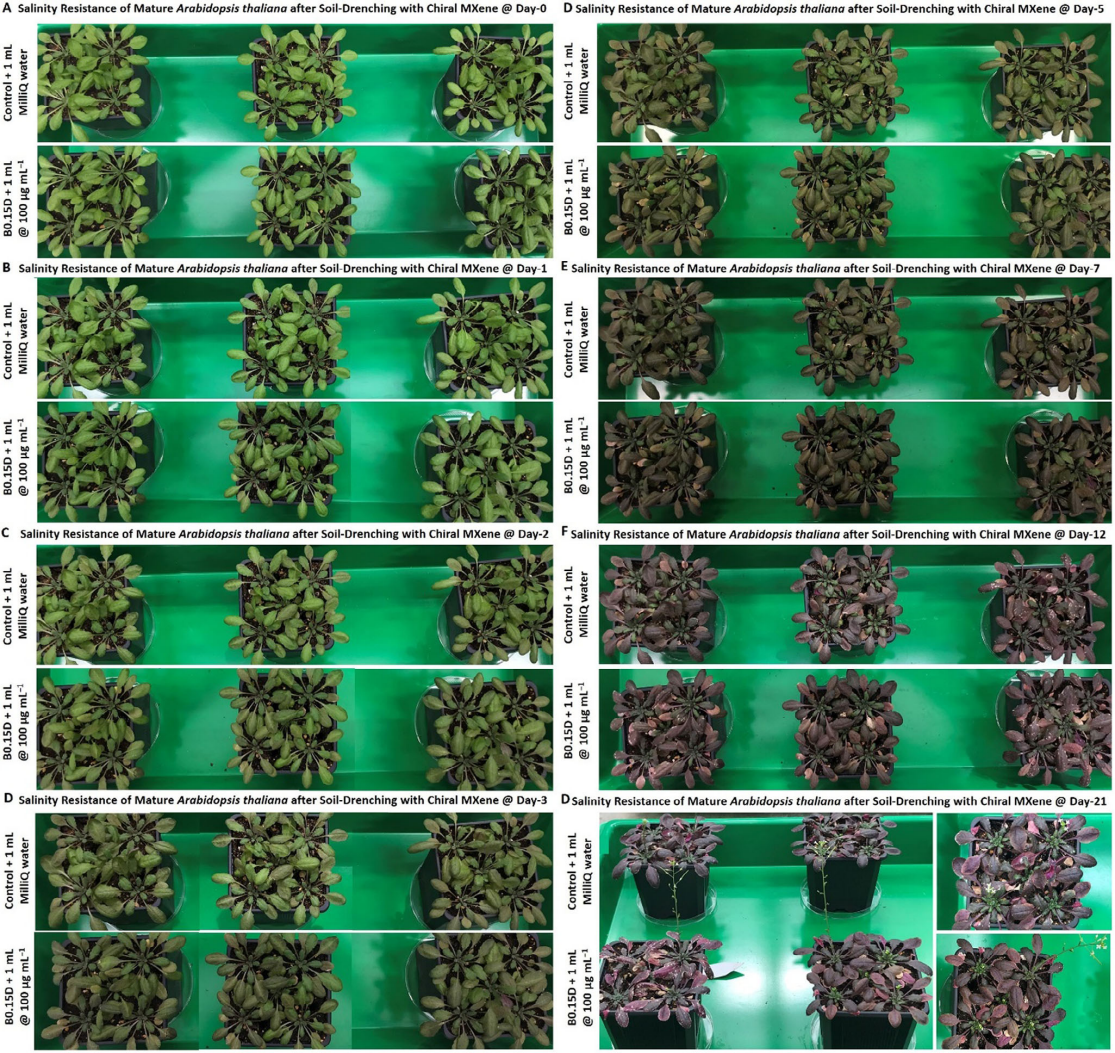
*In‐planta* short‐, mid‐, and long‐term salinity‐resistance bioactivity impact of the B0.15D aqueous collides in *Arabidopsis thaliana* under greenhouse conditions. A–H) Assessment of the plant's tolerance to continues extreme abiotic salinity stress conditions for up to around three weeks post soil‐drenching treatment inside a standard greenhouse. Interaction of these chiral MXene colloids at the concentration of 100 µg mL^−1^ with young and mature *Arabidopsis thaliana* (3–4 weeks old) was qualitatively monitored compared to control plants, and their impacts on these plants’ overall growth, flowering, and aging were observed by digital imaging.

**Figure 14:**
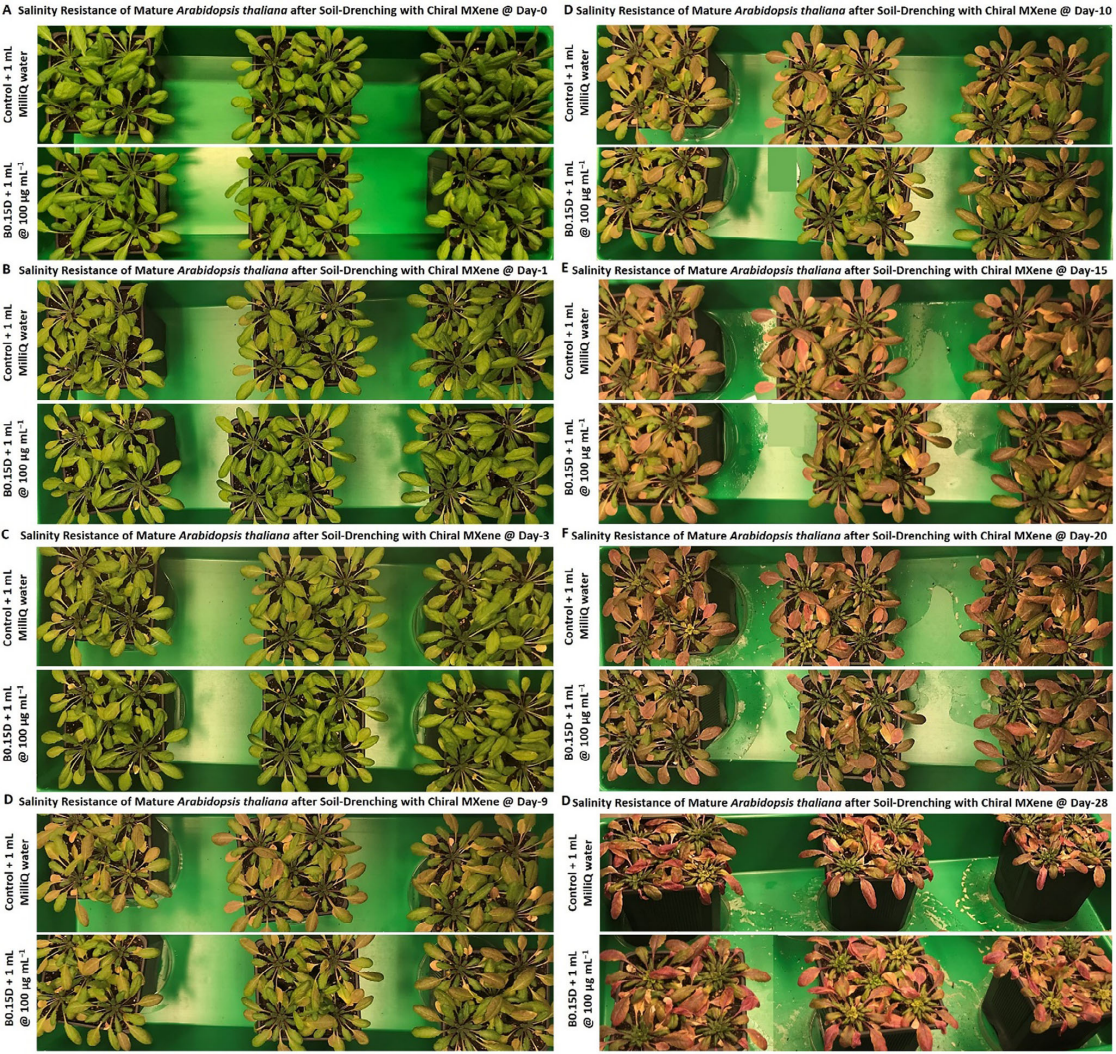
*In‐planta* short‐, mid‐, and long‐term salinity‐resistance bioactivity impact of the B0.15D aqueous collides in *Arabidopsis thaliana* under standard climate chamber conditions. A–H) Assessment of the plant's tolerance to continues extreme abiotic salinity stress conditions for up to around three weeks post soil‐drenching treatment inside the climate chamber. Interaction of these chiral MXene colloids at a concentration of 100 µg mL^−1^ with young and mature *Arabidopsis thaliana* (3–4 weeks old) was qualitatively monitored compared to control plants, and their impacts on these plants’ overall growth, flowering, and aging were observed by digital imaging.

As shown in **Figures** **13** and **14**, under the applied greenhouse and clime chamber salinity conditions, the plants in both control and B0.15D‐treated groups stayed relatively healthy and experienced natural aging for around one week of post‐interval treatments under the greenhouse conditions and after two weeks inside the climate chamber. As can be seen in these figures, after around day 12 of post‐treatment inside the greenhouse and day 21 at the climate chamber, the imagining of the *Arabidopsis thaliana* plants showed the relative pre‐mature leaf senescence by averaging occurred in older leaves, falling off much earlier than usual aging process as compared to the plants in our long‐term phyto‐compatibility assessments of non‐treated *Arabidopsis thaliana*.

Further, several soil‐drenching intervals for the plants with 200 mm of NaCl aqueous solutions, which is in the actual stress threshold of many agricultural regions with salinity conditions, likely result in reduced water uptake in plants as high concentrations of Na⁺in the soil can reduce the water potential to be root absorbed, causing the simultaneous experience of crossover drought‐like and salinity stress in plants. Consequently, despite the presence of adequate water in the soil through NaCl‐watering intervals, the plants started to show slight wilting symptoms because of the impaired water‐absorption capacities under salinity stress. Interestingly, as can be seen in Figure 13H, the plants treated with these chiral MXene colloids at day 21 post‐treatment showed a relatively enhanced tolerance against this abiotic stress, showing a more efficient flowering process along with less severe damage symptoms compared to the plants in the control group maintained under salinity‐imposed greenhouse conditions.

Moreover, our assessment of these experimental and control plants under salinity stress conditions inside the climate chamber aligns with *Arabidopsis thaliana*’s tolerance under greenhouse conditions. As shown in Figure 14, the stress symptoms and salinity‐induced damages in these chiral MXene‐treated plants are relatively lower than the control plants, especially regarding the plant's growth and development of their newer/younger leaves. Additionally, when the *Arabidopsis thaliana* plants were maintained under the climate chamber conditions, our observations exhibited a slightly improved turgor and water retention presumably due to the enhanced water absorption and hydration, referring to higher chiral MXene‐enhanced ability to hold more amount of absorbed water and remain firmer during the stress condition. Together, these qualitative data suggest an overall plant tolerance/resilience enhancement against continuous salt stress conditions.

### 2.15 In‐Seedlings Salt‐Stress Resistance‐Inducing Bioactivity of Optimal Chiral MXenes

**Figure 15:**
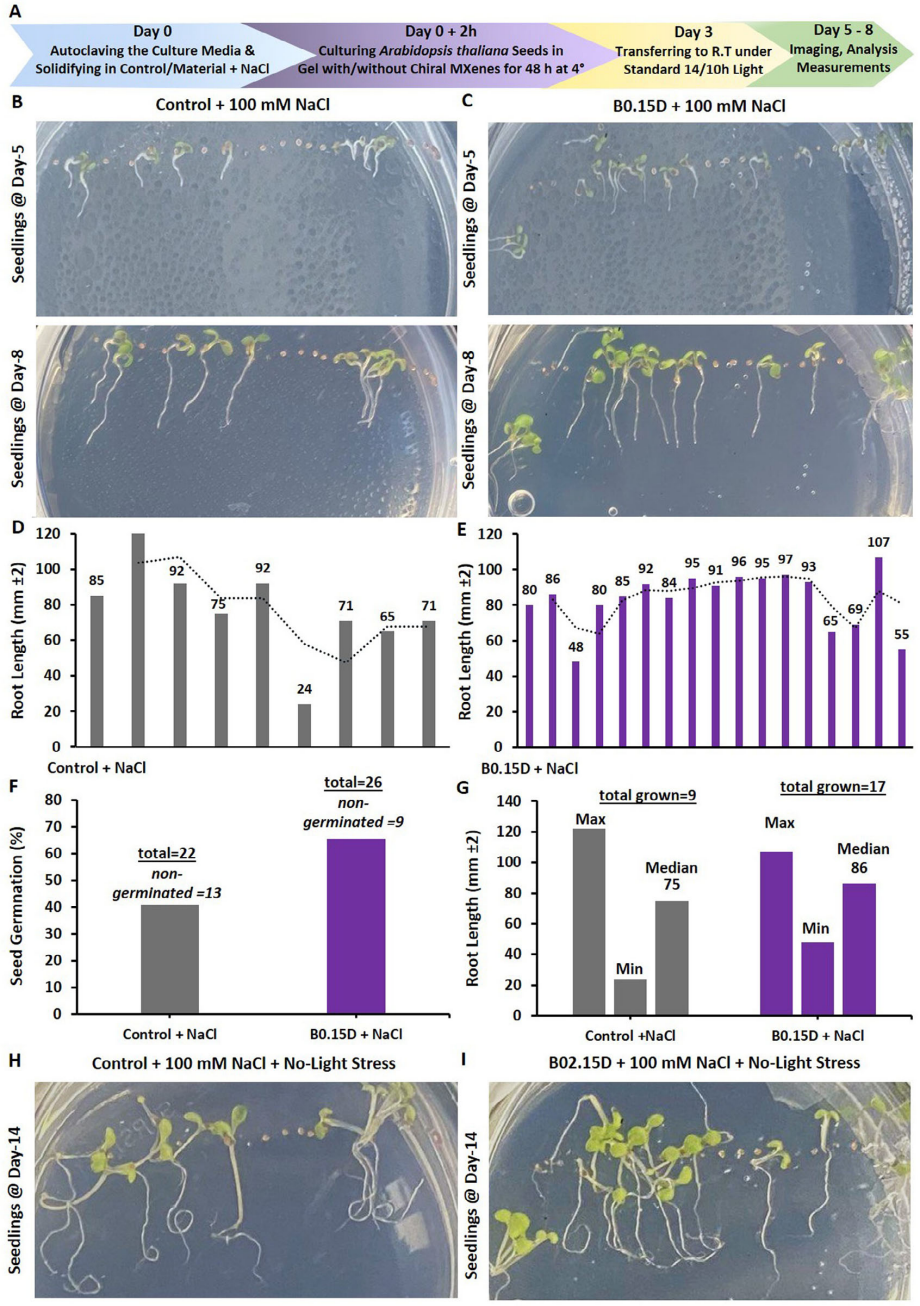
The experimental timeline and biostimulant impact of optimal chiral MXenes on enhancing *Arabidopsis thaliana* seedlings’ growth and maturation under salt stress conditions at different short‐ to long‐term time points. A–I) The col‐0 seeds were line‐cultured in solidified media with/without B0.15D chiral MXene colloids at a concentration of 88 µg mL^−1^. The camera images and related seed germination and root length analysis showed a remarkable enhancement in the maturation of the chiral MXene‐treated seedlings at day 8 post‐treatment. Moving average trendlines were obtained by Excel software and used to compare forecast models in both experimental and control groups. ImageJ software was used to measure the camera images of the roots using a similar trend. The max, min, and median of the roots also suggest the bioactivity of both tested chiral MXenes with a slight superiority of B0.15D colloids (n: is equal to at least twenty‐five per sample).

To further study the capability of optimal chiral MXene to enhance plant tolerance to salt stress and validate our results from the *in‐planta* salinity bioassay, we elucidated the impact of this biomaterial to enhance the tolerance of seedlings to salt stress where the seed‐to‐seedling transition was performed with the presence of 100 mM NaCl solutions. This bioassay was specifically aimed to assess the direct effect of this material from the very early to developmental stages. **Figure** **15**A displays the experimental timeline of this bioassay. As can be seen in Figure 15B–G, the imaging of these seedlings at days 5 and 8 post‐treatment and their corresponding seed germination and root length quantification analysis on day 8 show a remarkable enhancement in the overall growth and maturation of as‐treated seedlings with B0.15D compared to control samples, suggesting an improvement in the tolerance of *Arabidopsis thaliana* to the applied salt stress. Interestingly, the image of these seedlings on day 14 of treatment depicts that the germination in the B0.15D group is almost double that of those control seedlings (see panels H and I). Taken together, the results of these salt‐stress bioassays suggest that treatment with these optimal chiral MXene colloids (either at early seed stages or when foliar‐sprayed on mature plants) could effectively enhance their tolerance to abiotic salinity stress.

Moreover, we wanted to validate and compare the intrinsic capability of these chiral MXene colloids to enhance the plant tolerance to abiotic stresses with another potential bioactive substance. For this reason, we also included chitosan 661‐Cl in the performed salt‐stress bioassays to fairly compare with the bioactivity of our chiral MXenes. As shown in Figure S20 (Supporting Information), the seedlings’ overall germination, growth, and maturation, were qualitatively found to be higher in the group of seedlings treated with these chiral MXene colloids compared to control and chitosan 661‐Cl samples at day 5 and 8 post‐treatment. Our data show a remarkable enhancement in the tolerance of *Arabidopsis thaliana* seedlings to salinity stress after treatment with the B0.15D, suggesting promising biocompatibility, biostimulant properties, and bioactivity for enhancing plant tolerance/resilience to different abiotic stresses.

### 2.16 *In‐Planta* Light‐Stress Resistance‐Inducing Bioactivity of Optimal Chiral MXenes

Next, we assessed the intrinsic biostimulant activity of these chiral MXene colloids to enhance the plant's tolerance against continuous light‐stress conditions. In particular, the regular watering of mature *Arabidopsis thaliana* plants (3‐4 weeks old) in both control and experimental groups has been continued after soil‐drenching with ≈10 mL of pure water and the B0.15D aqueous colloids at two different concentrations of 10 and 100 µg mL^−1^. To simulate the agricultural regions with continuous light‐stress conditions, such as high latitude or tropical farming areas with extended daylight hours due to the exposure to prolonged periods of light and without sufficient natural darkness (lack of night cycles), which is essential for the growth processes of plants. This excessive and continuous light stress as a crucial abiotic condition can potentially disrupt the plant's physiological mechanisms/functions and adversely affect its yield, development, and quality. Thus, designing and developing plant biostimulants with more biocompatiblity and environmentally‐friendly profiles and their integration into farming practices of modern agriculture that experience significant levels of light‐stress conditions is advantageous to enhance plant tolerance against this abiotic condition and ultimately improve their crop productivity in more cost‐effective manners.

**Figure 16:**
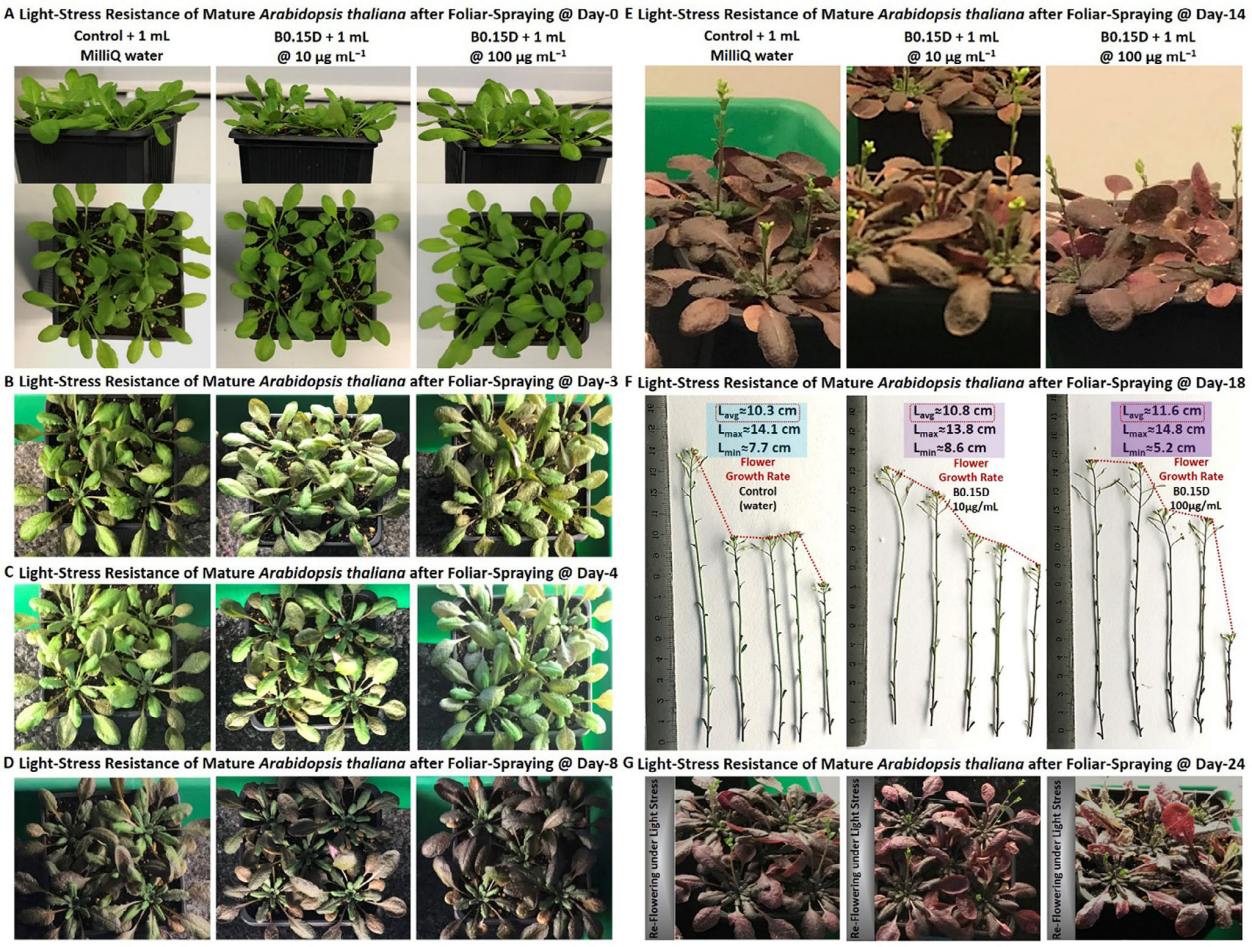
*In‐planta* short‐, mid‐, and long‐term excessive light stress‐resistance bioactivity of the B0.15D aqueous collides in *Arabidopsis thaliana* under continues 24 h day‐cycling light conditions. Assessment of the plant's tolerance to continues extreme abiotic light stress of night‐free cycling conditions for up to ≈25 days post soil‐drenching treatment. Interaction of these chiral MXene colloids at a concentration of 100 µg mL^−1^ with young and mature *Arabidopsis thaliana* (3‐4 weeks old) was qualitatively monitored compared to control plants, and their impacts on these plants’ overall growth, flowering, and aging were observed by digital imaging.

In this regard, we monitored the natural growth, aging, and overall physiological conditions of the mature *Arabidopsis thaliana* plants (3–4 weeks old) were artificially imposed to a standard excessive light‐stress condition (24 h nonstop day‐light exposure and without any dark cycles). The plants of both the experimental (once soil‐drenched with the B0.15D colloid) and water‐treated control groups were kept in a static mode, including standard watering with tap water every two to three days for around four weeks (at a total approximate age of two months) under the continues light‐stress simulations. The aging and subsequent flowering processes of these plants, as well as the adverse light‐stress‐inducing impacts and possible dark green‐to‐red leaf pigmentation and de‐coloring, were carefully monitored in the plants until severe eye‐visible shoot damage symptoms appeared (see **Figure** **16**). As shown in panels A to C, no significant effects were observed in as‐treated plants during the first few days of exposure to this abiotic light stress. The plants’ resilience against light stressors was no longer effective after around a week, and the symptoms gradually appeared in both experimental and control groups of *Arabidopsis thaliana* plants. As can be seen in Figure 16D, a significant and competitive leaf de‐coloring and pigmentation was observed at day 8 post‐treatment under dark‐cycle‐free light stress. No particular visual differences were observed between the B0.15D‐treated and control plants.

Furthermore, the first flowering cycle, which has been normally observed to develop at around week 2 post‐treatment was allowed to further growth under the applied light‐stress conditions (see panel E). Upon significant development of this chiral MXene or pure water‐treated *Arabidopsis thaliana*, the flowers in each group were cut from the same position, and their height was measured accordingly. As shown in Figure 16F, the average length of flowers cut from the five individual plants of each group are found to be 10.3, 10.8, and 11.6 cm in the control, B0.15D at 10 µg mL^−1^, and 100 µg mL^−1^, respectively. These observations suggest that the applied soil treatment of plants with these chiral at both tested concentrations could not only enhance the plant tolerance to severe light stress conditions but also effectively contribute to a relative increase in the flowering growth rate in the shoot parts.

While, our assessment of overall eye‐visible plants’ physiological conditions under this light stress revealed relatively severe damage to the shoot sections (leaf and stem) of all groups at logger exposures (e.g., at day 24 post‐treatment), the soil‐drenched plants with these chiral MXene colloids showed a slightly higher tolerance to continues light stress compared to control samples, especially in the newly‐developed and younger leaves (see Figure 16, panel G). This data suggests the enhanced plant tolerance to the imposed light stressor in plants soil‐drenched with the B0.15D colloids. Taking together, these multiple biostimulation activities highlight the potential capacity and impact of these chiral MXene colloids in enhancing plant tolerance and their defense/resistance responses to diverse abiotic stress conditions.

### 2.17 A Roadmap to the Rationale of these Chiral MXene Biomaterials and Perspectives to their Future Considerations in Agricultural Settings

In this section, we strived to further describe the rationale behind using the cysteine amino acid beyond other biocompatible amino acids and naturally occurring chiral compounds that have been commonly used as generally recognized as safe (GRAS) in producing many food and commercial cosmetic products as an antioxidant additive or flavor enhancer, and agricultural biostimulants as plant growth/defense and crop yield boosters. In this context, extensive practical applications and ethical consideration have been given to the amino acids of aspartic acid, glutamic acid, alanine, serine, tyrosine, phenylalanine, proline, and cysteine. Among them, significant attention has surged to cysteine amino acid due to its unique physicochemical and biological properties that make it particularly advantageous for these specific applications and to enhance the biocompatibility and bioactivity of functional nanomaterials. This superiority encompasses the unique presence of disulfide bonds in the thiol group (−SH)‐containing structure of cysteine to feasibly form disulfide‐based bonds (−S−S−) with other cysteine‐based molecules, contributing to enhanced protein formation and stability factors. In addition, the cysteine amino acid has a relatively small structure alongside high water solubility, making it highly beneficial for colloidal applications in agriculture.

Furthermore, in addition to its high levels of phyto‐compatibility, the cysteine residues have intrinsic antioxidant properties and support scavenging free radicals in biological environments, protecting the cells from excessive oxidative stress and enhancing the long‐term biocompatibility specifications of its composites. Additionally, cysteine is a known precursor for synthesizing glutathione, which is known as a pivotal antioxidant in plants, contributing to neutralizing the produced ROS under harsh abiotic stress at early stages or sustained drought and salinity conditions.^[^ ^100^, ^101^, ^102^, ^103^, ^104^, ^105^, ^106^, ^107^, ^108^, ^109^ ^]^ Thus, cysteine has been found to effectively contribute to plant stress resistance enhancement, promoting their resilience and regular growth and aging under severe environmental conditions, including continuous drought and salinity. All these properties have supported it as a desirable candidate in nanomaterials‐based agricultural biostimulation strategies to alleviate abiotic stress in actual crops in more eco‐ecofriendly manners. Taking all these accounts into consideration highlights the potential and promising capacity of chiral MXene‐based biomaterials with enhanced stability and multifunctional bioactivities for stimulating plants/crops. An almost similar trend of surface‐functionalization and modification is expected for the other chemical compositions of MXene nanosheets; however, the feasibility of this potential chirality induction strategy needs to be precisely studied for each MXene composition. The detailed plant biostimulatory, stress enhancement, and immunomodulatory properties of developed chiral MXene materials are required to be further investigated through mechanistic studies. Optimizing the processes, long‐term biological/immunological/plant defense responses, and comparison with other chiral‐active amino acids or substances are suggested as future outlooks.

## 3 Conclusion

To conclude, herein, we reported for the first time a versatile and facile strategy for induction of right‐ or left‐handed chirality into the surface of MXene nanosheets toward developing next‐generation carbon‐based agricultural biostimulants with stable mixed‐low‐dimensional structures and enhanced phyto‐compatibility in the aqueous colloidal forms. The multi‐, oligo‐, and mono‐layered surfaces of Ti_3_C_2_T_x_ MXene (as a typical representation of MXene prototypes’ chemical compositions) are functionalized with different concentrations of citric acid monohydrate to create significant amounts of carboxyl‐based terminations to subsequently bind with D‐/L‐cysteine amino acid molecules. These mixed‐low‐dimensional chiral MXene heterostructures and derived chiral quantum dots enriched with surface titanium oxide are rationally modified based on these bioactive amino acid ligands.

Using DFT calculations and EIS measurements, we proposed a possible interaction at Ti_3_C_2_T_x_‐cysteine interfaces and evaluated the surface and electronic properties of chiral MXene colloids. Our findings elucidated that the introduced chirality in these MXene possess high levels of biocompatibility with the seeds, seedlings, and plants of *Arabidopsis thaliana* at different concentrations ranging up to 100 µg mL^−1^ alongside enhanced colloidal resistance against debonding/oxidative, degradation, and structural decomposition, especially in aqueous media. Due to the applied chemistry manipulation, the optimal chiral MXene colloids could effectively interact with the seeds, seedlings, and plants of model samples for multifunctional phyto‐stimulations. In addition, these chiral MXene colloids could effectively trigger the stomatal‐closure bioactivity and related ROS‐induced mechanism in plants, which alongside their growth and development enhancement could make them more resistant to severe drought, salinity, or light stresses.

Our assessments of the long‐term phytocompatibility of these colloids suggested that their direct culturing, foliar‐spraying, or soil‐drenching treatments in the tested models not only impose any visible adverse effects on their overall physiological conditions, growth, or aging process but also considerably contribute to enhancing their seed‐to‐seedling transition and maturation, as well as stress resistance against severe environmental conditions. The results of this pilot study may open new avenues of studies toward improving these chiral MXene biomaterial designs and developing further compositions for diverse applications in agriculture, including next‐generation nano‐biostimulants and plant boosters, as well as in other sectors of biology and nanomedicine fields. Further investigations into the bioactivity of chiral MXenes in actual crops and underlying biological/immunological responses and their biocompatibility optimization, as well as long‐term safety for soil, beneficial organisms, and surrounding environments, are essentially considered future works.

## 4 Experimental Section

### Preparation of Aqueous Dispersions of Ti_3_C_2_T_x_ MXene‐Based Nanosheets

The powder of Ti_3_C_2_T_x_ MXene‐based nanosheets purchased from Laizhou Kai Kai Ceramic Material Co. Ltd, China, was dispersed in MilliQ water at a concentration of 1000 µg mL^−1^ through magnetic stirring of the mixture for 5–10 min, followed by a bath ultra‐sonication for ≈1 h at room temperature to obtain a multi‐, few‐, and mono‐layered Ti_3_C_2_T_x_ MXene suspensions using a EMAG ultrasonic machine (EMAG, Emmi‐D20Q, Germany) with a ultrasonic frequency of 37 kHz (total power consumption, ultrasonic effective‐power, and max ultrasonic peak impulse from the wave of 90, 30, and 240 W, respectively). For biological experiments, the powder was first surface sterilized by a short cycle of ultraviolet (UV) light at the wavelength of ≈250 nm using a Stratalinker cross‐linker machine for ≈5 min. The obtained water‐MXene mixture sample was adjusted to the desired concentrations, and the stocks were then transferred to different bottles and stored dark at four degrees to slow down and minimize the aqueous oxidation reactions for further sonication and surface modification treatments.

### Surface‐Modification of Ti_3_C_2_T_x_ MXene Aqueous Dispersions with Carboxyl Termination

To functionalize the surfaces of these MXene nanosheets with carboxyl‐based functional groups, two different bath‐ and probe‐sonication treatment processes were used. Briefly, the MXene nanosheet dispersions in both bath‐ and probe‐sonication groups were treated for ≈30 to 40 min (operated continues pulsing) and 120–150 min (operated continuously during the first hour, then switched to a 5 min continues/2 min On/Off pulsing mode every 2 to 5 s during the next hour of operation, and resumed continuous pulsing for the remainder of treatment period), respectively. The bottles were kept dark using aluminum foils during the sonication process. The probe‐sonication of MXene dispersions was performed in an ice box to keep the bottles cool and avoid unwanted aqueous oxidation/excessive degradation due to the heating effects caused by the probe‐ultra‐sonication (BANDELIN SONOPLUS HD 70 machine, SK70 probe, UW 70). Next, different molarities of citric acid monohydrate solution (C_6_H_8_O_7_·H_2_O, Sigma‐Aldrich, C1909, MW: 210,14 g mol^−1^) were prepared (0.15, 0.3, and 1 m) by magnetic dissolving the powder in pure water followed by stringing for 15 min and stocks filtration using the typical 0.2 µm filters. To prepare carboxyl‐functionalized Ti_3_C_2_T_x_ MXene nanosheets dispersions in the group of bath‐sonication (B.015 and B0.3) and probe‐sonication (P.015 and P1) treatments, 100 mL of these citric acid aqueous solutions were gently mixed with 200 mL of as‐prepared MXene dispersion stock using 25 mL pipettes and magnetically stirred at 750 rpm at room temperature for ≈24 h to prepared carboxyl‐functionalized MXene‐based colloids. After the reactions, the pH of samples was measured using a pH indicator Fix 0–14 (ROOT, Laborbedarf), and the resultant colloidal suspensions were centrifuged at 4500 rpm (Eppendorf 5804R, rotor A‐4‐44) for 20–30 min for 3 to 5 times to thoroughly wash the flakes and eliminate the unbonded and or remaining bigger citric acid traces (at around pH of pure water). The obtained precipitants of the carboxyl‐treated MXene flakes were then dispersed in the same amount of MilliQ water and final mixture stocks were stored in the dark at four degrees in a cold room for microstructural characterizations and the rest of the surface modification experiments.

### Preparation of Right‐ or Left‐Handed Chiral MXene‐Based Colloids

To further surface modify and introduce the chirality into the surfaces of these carboxyl‐based functionalized MXene aqueous colloids, different right‐ or left‐handed sources of D‐ or L‐cysteine amino acids were used. Briefly, these colloids in both groups of bath‐sonication (B.015 and B0.3) and probe‐sonication (P.015 and P1) were separately treated with D‐cysteine (C_3_H_7_NO_2_S, Sigma‐Aldrich, 30 095, MW: 121.16 g mol^−1^) or L‐cysteine (C_3_H_7_NO_2_S, Sigma‐Aldrich, 168 149, MW: 121.16 g mol^−1^). In particular, stable chirality was introduced and artificially inducted in these MXenes through the well‐established EDC/NHS cross‐linking method.^[^ ^110^ ^]^ This facile and universal strategy for surface modification of active low‐dimensional structures had been previously reported for graphene‐based materials.^[^ ^111^ ^]^ Herein, this EDC/NHS was used with slight modification and parameter adjustments. In particular, Different novel chiral Ti_3_C_2_T_x_ MXene‐based biomaterials were designed and prepared in the form of aqueous colloids, spin‐coated thin films, and dried flakes at around room temperatures and without using complex laboratory equipment.

To impart chirality to these carboxyl‐functionalized MXenes, their enriched surfaces with various functional and active terminations were connected or integrated with the amine group of these amino acid‐based ligands. The chirality induction was performed by adding scalable 4 mL of the prepared 26.64 mm EDC solution (C_8_H_1__7_N_3_·HCl, Sigma‐Aldrich, 8.00907.0001, CAS‐No: 2595‐53‐8, MW: 191.70 g mol^−1^) into 50 mL of as‐functionalized aqueous MXene colloids. The mixtures would be stirred for ≈15 to 30 min and then the same amount of 50 Mm NHS solution (C_4_H_5_NO_3_, ThermoFisher Scientific, +98%, 157270250, MW: 115.09 g mol^−1^) was added to each mixture of the prepared EDC‐carboxyl‐MXene solutions and mixed ≈30–45 min. Lastly, 4 mL of the prepared L‐cysteine or apparently D‐cysteine aqueous solution at the concentration of 26.64 mm were added into the obtained EDC‐carboxyl‐MXene‐NHS solutions, and the mixtures were stirred ≈30 to 45 min. The resultant colloidal suspensions were centrifuged at 4500 rpm for 30 min for three to five times to thoroughly wash the chiral MXene‐based colloids and remove the unboned and or remaining bigger traces and surplus chiral ligands and EDC/NHS traces for purification. The precipitants were then dispersed in fresh MilliQ water at the final concentration to obtain high‐quality aqueous chiral MXene colloidal biomaterials. The stocks were then stored dark (covered with aluminum foil) at four degrees for microstructural characterizations and biological experiments.

### Physicochemical Characterization of Pristine Nanosheets, Carboxyl‐Functionalized MXenes, and Mixed‐Low‐Dimensional Chiral MXene Colloidal Heterostructures

The microstructure and morphology of pristine Ti_3_C_2_T_x_ MXene‐based nanosheets (powder, aqueous dispersions, and spin‐coated thin films) and the prepared colloids were characterized by a TEM machines (Zeiss 900 running at 80 kV with a Troendle 2 K camera) and a SEM machine attached with EDS detector system (Zeiss Leo Crossbeam1540 with a Bruker EDX equipped with an XFlash10). The phase structure and characterization pattern of the colloidal samples were characterized by a GIXRD machine (Bruker D8 Discover with copper anode at 40 kV and 30 mA). To do so, 100 to 200 µL of these aqueous colloidal suspensions at the concentration of ≈4 mg mL^−1^ was dropped on new glass coverslips and then standing for 4 to 5 min, followed by spin‐coating for ≈60–120 s at a moderate spinning rate of 3000 rpm, and subsequent 10 to 15 s of spinning at a higher rate of 6000 rpm. The coated glass was then dried under the vacuum of spin‐coater machine at around room temperature. To measure the GIXRD of these thin films, a Goebel mirror was attached on the primary side and yielding a parallel beam geometry of width of a maximum of 0.6 mm due to slits. On the secondary side, there was a 1.2 mm detector slit with a beam size of ≈0.6 × 20 mm. The measured GIXRD spectra at the 2‐theta of 5 to 80°, were plotted using the RAWGraphs open source data visualization framework software.^[^ ^112^ ^]^

Furthermore, the surface chemistry and functional groups of the samples were characterized using an X‐ray photoelectron spectroscopy (XPS, Omicron System) and a FTIR machine (Nicolet iS50 Spectrometer). To do this the aqueous colloidal samples were first centrifuged at 10000 rpm for 60 min, where then deposited onto carbon tape and dried in glove box (<0.5 ppm of water and oxygen) under argon gas at 80 °C to prevent unwanted oxidation, and loaded on machines’ sample holders.

The structure of materials was further characterized using Raman spectroscopy (StellarNet 532 nm device, available in the Multifunctional Energy Storage Lab at the University of Tulsa). To do so, the samples in the form of aqueous colloidal dispersions were poured into special vials and then placed in the device's sample holder for measurements. The spectrometer was powered on, using the StellarPro‐V2.2.4 software to select the 532 nm excitation source, allowing the system to warm up. The integration time of all Raman experiments was set to 500 ms, and the number of scans to average was set to fifteen for optimal signal acquisition. Before the measurements, the background spectrum was acquired by a blank (empty vial) and accordingly subtracted from the sample data. To ensure a clear signal during acquisition, the laser power and focus were adjusted as needed. The peaks in the collected spectra were identified and compared with reference spectra. Furthermore, the optical absorption analysis of the samples was measured at the wavelength ranging from 280–980 nm using a UV–vis spectroscopy (Jasco Spectrophotometer Machine, V‐630) and by transferring ≈1 mL of the samples in both control and experimental groups of materials into the standard square cuvettes. The long‐term dispersibility and colloidal stability of samples were also qualitatively observed by digital imaging.

### Left‐/Right‐Handed Chirality Characterization by CD Spectroscopy

The induced left‐/right‐handed chirality characterization of the samples was measured using CD spectroscopy. The CD spectra were recorded by a Jasco J‐1500 CD spectropolarimeter (Hachioji machine, Tokyo, Japan), ensuring consistency by conducting the measurements on the same day with identical sample preparation to avoid any significant variability from sample degradation and or instrument fluctuations. The instrument parameters settings included a scan rate of 10 nm per minute, with each spectrum averaged over five accumulations to improve the signal‐to‐noise ratio. A band‐width of 1 nm was used for sufficient resolution, and a time constant of 4 s was applied to reduce noise and produce smoother data. Measurements covered the wavelength range of 200–400 nm, with baseline correction performed using the solvent alone to account for any background interference. These standardized condition ensured accurate data collection and comparable results throughout the analysis.

### DFT Calculation on the Proposed Interaction of Functionalized Ti_3_C_2_T_x_ MXene with Cysteine Molecules

To understand the electronic properties and propose surface interaction of functionalized Ti_3_C_2_T_x_ MXene‐based nanosheets with cysteine molecules, DFT calculations were performed using the commercially available software VASP, available in the Multifunctional Energy Storage Lab at the University of Tulsa. Two different Ti_3_C_2_T_x_ structures were considered in the current study, one functionalized −OH and O═C groups and another one with cysteine attached to the functionalized surface of this MXene type. The distribution of total electronic charge density properties around the dominant atoms and possible bindings on surface functional groups were evaluated to assess a possible interaction of these chiral ligands with MXene interfaces. Further, for a better understanding of this surface interaction, the binding energy of the cysteine molecule was also calculated on the surface of functionalized MXene using the following equation:

(4) $$E_{\mathrm{binding}}=E_{\mathrm{MXene}+\mathrm{cysteine}}-\left(E_{\mathrm{MXene}}+E_{\mathrm{cysteine}}\right)\;\;\;\;\;\;\;\;\;$$

### EIS and Ionic Conductivity Measurements at Chiral MXene Interfaces

A series of battery cells were assembled using stainless steel electrodes to evaluate the performance of these electrolytes. These cells were first subjected to an electrochemical evaluation under OCP conditions for 5 min. This step allowed the system to stabilize and determine the equilibrium potential between the electrodes when immersed in the electrolyte. After the OCP stabilization period, the cells underwent EIS analysis in CHI 660E potentiostat/galvanostat available in the Multifunctional Energy Storage Lab at the University of Tulsa. In this test, the applied direct current (DC) potential was set to the measured OCP, representing the equilibrium potential of the electrodes in the given electrolyte. Additionally, a small alternating current (AC) signal with an amplitude of 5 mV was superimposed onto the DC potential across a frequency range, from 10 kHz to 100 mHz, ensuring continuous perturbation within this range. This frequency sweep enables the characterization of various electrochemical processes within the battery system, including charge transfer resistance, electrolyte resistance, and electrode‐electrolyte interfacial behavior. The high‐frequency region (near 10 kHz) primarily reflects the bulk electrolyte resistance, while the low‐frequency region (down to 100 mHz) provides insights into diffusion‐controlled processes and interfacial charge transfer kinetics. By defining an equivalent circuit and fitting the impedance spectra obtained from the EIS measurements, a key electrochemical parameter (bulk resistance) was measured for the chiral MXenes samples in a comparable manner with pristine Ti_3_C_2_T_x_ MXene in aqueous dispersions.

Furthermore, in the Nyquist diagram (‐Z" vs Z'), the starting point of the Nyquist curve (where Z" is zero) represents the bulk resistance, which corresponds to the resistance of the electrolyte. To measure this, an equivalent circuit needs to be defined based on the possible reactions occurring in the system. After establishing the circuit, the bulk resistance data were extracted and converted to the resistance into ionic conductivity using the following equation:

(5) $$\begin{array}{c} \sigma_{\left(\mathrm{Ionic}\;\mathrm{Conductivity},\;S/\mathrm{cm}\right)}=L_{\left(\mathrm{Distance}\;\mathrm{between}\;\mathrm{the}\;\mathrm{electrodes},\mathrm{cm}\right)}\\ /A_{\left(\mathrm{Cross}-\mathrm{sectional}\;\mathrm{area}\;\mathrm{of}\;\mathrm{the}\;\mathrm{electrolyte}\;\mathrm{between}\;\mathrm{the}\;\mathrm{electrodes},cm^{2}\right)}\\ \times \;R_{\left(\mathrm{Measured}\;\mathrm{bulk}\;\mathrm{resistance}\;\mathrm{from}\;\mathrm{the}\;\mathrm{Nyquist}\;\mathrm{plot},\mathrm{ohm}\right)} \end{array}$$

### In Situ Seed Coating, Seedling‐Sprouting, and Germination in Murashige‐Skoog (MS) Media and Assessment of Short‐ and Mid‐Term Biocompatibility Properties of theses Chiral MXenes

To assess the capability of these chiral MXene‐based aqueous colloids for seed coating and their short‐, mid, and long‐term biocompatibility with Columbia‐0 *Arabidopsis thaliana*, the seeds were surface‐sterilized using a typical protocol using a mixture of ethanol 70% and sodium hypochlorite 3%, followed by thorough washing with distilled water (dH_2_O). The seeds were then transferred to 6‐well plates (n: is equal to 20 to 30 per well) containing half‐strength MS media (Duchefa Biochemie, Gamborg B5 vitamins Haarlem, Netherlands), supplemented with 2‐(N‐morpholino) ethanesulfonic acid (MES) and 1% sucrose. Different concentrations of these aqueous colloids (40 and 100 µg mL^−1^) were also added to the mixtures with the same amounts of water added instead in control groups. Accordingly, the plates were sealed with Parafilm and then incubated in a climate chamber under constant static condition (except for the microscopy or imaging at the interval time points) in a climate chamber (14 hours light at 21 °C with 120 µE/m^2^/s, and 10 hours dark at 18 °C) for up to two weeks. The seed coating, seedling sprouting, seedling germination, and their maturation processes were monitored and qualitatively visualized at different interval time points of day 0, 1, 3, 5, 7, 10, and 14 post‐treatment using bright‐field optical microscopy (Olympus SZ61 and CKX41, KERN Optics OBL 156) and camera imaging.

### *In‐Planta* Stomatal Closure‐Inducing Bioactivity of these Optima*l* Chiral MXene Colloids

To assess the bioactivity of these chiral Ti_3_C_2_T_x_ MXene‐based aqueous colloids at very early stages of interaction with *Arabidopsis thaliana* plants (e.g., 1 h post‐foliar‐spraying), the mature plants (3 weeks old, grown at the climate chamer 16/8 h photoperiod at 21 °C) in both control and experimental groups were equally watered with a sufficient amount of tab‐water to naturally induce stomatal opening through the plant intrinsic biological/immunological mechanisms. After 1 h, the control plants were foliar sprayed with ≈1 mL of pure water, and the chiral‐introduced colloids treated the plants of the same age via foliar spraying at a concentration of 10 µg mL^−1^, followed by ≈60 min of function times on bench incubation at the room temperature. Random leaves from each plant (four from different individuals in the pots of each group) were cut and blended in ≈200 mL of distilled water for ≈10 s. The plant cells and bio‐compound species were then transferred on clean glass cover slides, and their stomata were carefully observed; their apertures were measured via bright‐field optical microscopy imaging at the 40X zoom (KERN Optics model OBL 156) and the ImageJ software (n: is equal to at least 70 and maximum 140 per group). The data and comparison between the control and experimental groups were statically analyzed by GraphPad Prism USA, based on the standard method of Kruskal‐Wallis and Dunn's multiple comparisons tests. In addition, the microscopic interaction of mature *Arabidopsis thaliana* plants foliar‐sprayed with optimal MXene chiral colloids was visualized by microscopic imaging at different magnifications. Glass slides of isolated cells from leaves in control and experimental groups were prepared using the nail polish impression method.

### *In‐Planta* ROS Measurement and Eliciting Bioactivity of Chiral MXene Colloids

To measure ROS generation in plants by the effect of chiral MXenes and analyze their associated eliciting bioactivity, the average of seven leaf disks per group (each from individual leaves) of *Arabidopsis thaliana* plants (grown at the climate chamer 16/8 h photoperiod at 21 °C) in control and experimental samples were prepared by gentle cutting and pressing with a cork borer (Ø5 mm) on the lamina part of the fully grown detached leaves. One leaf disk per well was then transferred to a 96‐well microtiter plate containing 100 µL of pure water. The plate was incubated overnight at 21 °C. The eliciting assay was carried out by replacing the water with 200 µL of a 1:1 mixture of a putative elicitor solution at different concentrations and 0.5 mm of luminol derivate L‐012 (8‐amino‐5‐chloro‐7‐phenylpyrido[3,4‐d] pyridazine‐1,4(2H,3H) dione) and 20 µL of horseradish peroxidase (HRP) at the concentration of 10 µg mL^−1^ in 10 mm 3‐(N‐morpholino)propanesulfonic acid (MOPS)/potassium hydroxide (KOH) buffer at the of pH 7.4, as described previously.^[^ ^85^ ^]^ The production of ROS was quantified by a TECAN Spark Microplate reader by measuring each well for 1000 milliseconds every two minute over a total duration of ≈90 min at room temperature. The maximum ROS production value (relative light units, RLU_max_) in response to an elicitor‐active agent was read to compare the ROS‐induced eliciting intensity of these chiral MXene colloids.

### Assessment of Short‐ and Mid‐Term Bioactivity of Chiral MXenes on Seedlings Maturation

To further validate the biocompatibility results and assess the biostimulant impacts of optimal chiral MXene colloids on enhancing seed germination and seedling maturation, a line‐cultured seedling assay was performed. This experiment was designed to assess the direct biostimulant activity of optimal chiral MXene colloids on the early seed seedling transition and maturation stages grown at a standard laboratory condition. Briefly, *Arabidopsis thaliana* Col‐0 seeds (≈30 per group) were treated with/without these colloids at 88 µg mL^−1^ in a solidified MS‐agar media supplemented with 1% sucrose. The Petri‐dishes in both experimental and control groups were sealed with Parafilm to avoid contamination and the seeds were grown under a 14/10 h photoperiod at around 21 °C. The overall seedlings’ health, germination rate, maturation, and root length in chiral MXene‐treated seedlings were monitored and compared with the control samples at days 5 and 8 post‐treatment using camera imaging. On day 8 of treatment, the seedlings’ germination rate and root lengths were visualized by camera images and quantified using the online ImageJ software. Additionally, to compare the obtained results and validate the biostimulant activity of optimal chiral MXenes, their bioactivity was compared with the seedlings cultured in the presence of a commercially available chitosan‐based substance at the similar concentration and growth conditions (chitosan 661‐Cl, DA≈20%). This chitosan was kindly provided by Dr. Dominique Gillet (Gillet Chitosan, PIumaudan, France).

### Assessment of Short‐, Mid‐, and Long‐Term Biocompatibility of Optimal Chiral MXenes with Potted Plants

In the next experiment, the *in‐planta* biocompatibility of optimal chiral MXene colloids at the concentration of 100 µg mL^−1^ was assessed for up to ≈28 days when plants were once soil‐drenched with 10 mL of the material and the same volume of pure water in the control plants. To have a robust estimation of the growth, flowering, and overall aging/physiological processes of the treated *Arabidopsis thaliana* plants, these processes were monitored when the plants were maintained under standard greenhouse or climate chamber conditions. These greenhouse experiments were performed at plant research facilities at the Biological Science Faculty of Goethe University under a 14/10 h photoperiod. The climate chamber bioassays were conducted at standard conditions of a 14/10 h photoperiod and a light intensity of 100 µE m^−2^ s^−1^ (microsieverts per square meter per second, as photosynthetically active radiation). At different time points, the plant growth and conditions were monitored and visualized by camera imaging.

### Assessment of *In‐Planta* Drought Resistance Bioactivity of Chiral MXene Colloids

The bioactivity impact of optimal chiral MXene on enhancing plant tolerance to continuous abiotic drought stress at different greenhouse and climate chamber conditions was assessed. The experimental plants were once soil‐drenched with 10 mL of B0.15D aqueous colloids at the concentration of 100 µg mL^−1^. The *Arabidopsis thaliana* Col‐0 plants of the same age in control groups were soil‐treated once with the same amount of water. Briefly, the plants were first grown to maturity and then transferred into the laboratory to apply the direct soil treatment. The control and chiral MXene‐treated experimental plants were then transferred back to the greenhouse or climate chamber facilities, and the usual process of irrigation (watering every 2–3 days) was halted in these models. The experimental and control plants in the greenhouse group were regularly weighted through a digital scale at different interval time points, and the plant's overall conditions and flowering under the applied drought stress were eye‐monitored and visualized by camera imaging. After significant damage symptoms and leaf de‐coloring, the plants in all groups were re‐watered once or twice with a similar sufficient amount of water, and the observation process was continued until the flower development cycle and emergence/growth of new or younger leaves.

### Assessment of *In‐Planta* Salinity Resistance Bioactivity of Chiral MXene Colloids

The intrinsic bioactivity of these optimal chiral MXene aqueous colloids to enhance the plant tolerance to abiotic salinity stress was also assessed in soil‐drenched mature *Arabidopsis thaliana*. Likewise, for robust evolution, the salt‐stress resistance bioactivity of these colloids was tested in two different plant‐based models under the greenhouse and climate chamber conditions and compared with water‐treated control samples. The plants were first grown to maturity and then transferred into the laboratory to be applied with one cycle of soil‐drenching treatment with ≈10 mL of water (control) and the chiral MXene colloids (100 µg mL^−1^). The experimental control and plants were transferred back to the greenhouse or climate chamber facilities, and the usual irrigation process was halted. Instead, the plants in both groups received a 10 mL amount of water‐dissolved sodium chloride (NaCl) at 200 mm, which is a common salt level threshold for many agricultural and farming regions with a high level of environmental salinity conditions. The greenhouse plants were weighted through a digital scale at different interval time points, and the process of salinity‐induced damages and abnormal leaf folding beyond the usual aging process and developing newer/younger leaves were eye‐monitored, visualized by camera imaging, and quantified with the plants’ weight measurements.

### Assessment of In‐Seedling Salinity Resistance Bioactivity of Chiral MXene Colloids

To validate the results obtained from the *in‐planta* salt stress bioassay, in another experiment, the impact of these optimal chiral MXene colloids was evaluated on enhancing the tolerance of seedlings to salt‐stress conditions. This bioassay was designed to simultaneously assess the direct effect of the chiral MXene biostimulant material on the seed‐to‐seedling transition and seedling growth under salt stress. Briefly, the sterilized seeds (≈30 per group) were line‐cultured in a solidified sucrose‐supplemented MS media, including 100 mm of water‐dissolved NaCl solutions with and without chiral MXene colloids at the concentration of 88 µg mL^−1^. The Petri dishes were sealed with Parafilm to avoid contamination and medium dryness and kept under standard light with a 14/10 h photoperiod. The seedlings’ germination and growth and their root sizes were visualized on days 5 and 8 post‐treatment by camera images and the measurements compared with the control groups. In addition, the bioactivity of chiral MXene colloids was qualitatively compared with chitosan 661‐Cl at a similar concentration.

### Assessment of Excessive Light‐Stress Resistance of Plants Treated with Chiral MXene Colloids

The intrinsic bioactivity of these optimal chiral MXene aqueous colloids to enhance the plant tolerance to continuous light‐stress conditions was also assessed after soil‐drenching treatment of mature *Arabidopsis thaliana*. The light‐stress resistance bioactivity of these colloids was tested in a plant model of continuous 24 h daylight (night cycles‐free) conditions to simulate the farming regions with longer day‐time and or excessive light‐stress environmental situations under standard lab‐set up lighting of a 14/10 h photoperiod and a light intensity of 100 µE m^−2^ s^−1^ (24 h day‐light mode), where the plants of both groups were exposed to non‐stop light positioned at the height of ≈60 to 70 cm. Briefly, the plants were first grown to maturity of 3–4 weeks old and then transferred into the lab for applying one cycle of soil‐drenching treatment with ≈10 mL of water (control) and the colloids (100 µg mL^−1^). The water‐treated control and chiral MXene‐treated experimental plants were transferred back to the custom‐designed artificial daylighting setup, followed by the usual process of watering (every 2 to 3 days). This process was continued for around four weeks until the significant damage symptoms and leaf de‐coloring appeared, and the overall physiological condition, flowering, and aging processes were observed and compared under this light‐stress condition.

### Statistical Analysis

The statistical analysis of the stomatal‐closure bioassay was performed using GraphPad PRISM (GraphPad Software, Inc., USA) version 10.2.3. All data were presented as mean ± standard deviation (SD). No particular out‐of‐range data point or data set has been omitted from these statistical analyses. Asterisk signs indicate statistically significant differences between the groups and between the experimental samples **p < 0.01, ***p < 0.001, and ****p < 0.0001, and ns signify insignificant.

## Conflict of Interest

The authors declare no conflict of interest.

## Author Contributions

This study was conceptualized by A.R. and S.K. A.R designed and prepared the chiral MXenes. A.R. and A.A. characterized the materials and interpreted the data. Ak.R. and A.A. contributed to DFT calculations; H.S. and A.A. contributed to the Raman characterization and electrochemical measurements. A.R. and S.K. carried out the biological experiments, acquired the data, and conducted the formal analysis and biostatistics. A.R. and S.K. designed the figures and drafted the manuscript. A.R. and M.B. contributed to provide the experimental resources, laboratory facilities, and characterization data. A.R., S.K., Ak.R., H.S., M.B., and A.A. reviewed the final draft and approved its submission for publication. A.R and S.K are co‐first author to this work.

## Supporting information



Supporting Information

## Data Availability

Data presented in this study are available from the corresponding author upon reasonable request.
