# Supplementary material for: Induction of Chirality in MXene Nanosheets and Derived Quantum Dots: Chiral Mixed‐Low‐Dimensional Ti3C2Tx Biomaterials as Potential Agricultural Biostimulants for Enhancing Plant Tolerance to Different Abiotic Stresses
Source: Small. 2025 Apr 3;21(21):2500654. doi: 10.1002/smll.202500654 (PMC12105420; doi:10.1002/smll.202500654)
Supplement: Supplementary file 1 — Supporting Information [file SMLL-21-2500654-s001.docx]

**Electronic Supporting Information**

**Induction of Chirality in MXene Nanosheets and Derived Quantum Dots: Chiral Mixed-Low-Dimensional Ti_3_C_2_T_x_ Biomaterials as Potential Agricultural Biostimulants for Enhancing Plant Tolerance to Different Abiotic Stresses**

Alireza Rafieerad^a,b,c,1,*^, Soofia Khanahmadi^a,b,1^, Akif Rahman^d^, Hossein Shahali^e^, Maik Böhmer^a^, Ahmad Amiri^e,d^

*^a^ Institute for Molecular Biosciences, Johann Wolfgang Goethe Universität, 60438 Frankfurt am Main, Germany*

*^b^ Institute for Biology and Biotechnology of Plants, University of Münster, Schlossplatz 8, 48143 Münster, Germany*

*^c^ Regenerative Medicine Program, Institute of Cardiovascular Sciences, St. Boniface Hospital Research Centre, Department of Physiology and Pathophysiology, Rady Faculty of Health Sciences, University of Manitoba, Winnipeg, Canada*

*^d^ Department of Mechanical Engineering, The University of Tulsa, OK 74104, United States*

*^e^ Russell School of Chemical Engineering, The University of Tulsa, Tulsa, OK 74104, United States*

^1^ *Co-first author*

Contents:

***Supplementary File***: Supplementary Figures S1 to S20 and Table S1 to S3 (twenty-eight pages)

***Correspondence:**

Alireza Rafieerad, MSc, PhD

^1^ Institute of Molecular Biosciences, Faculty of Biological Sciences, Goethe University, 60438, Frankfurt am Main, Germany

^2^ Institute for Plants Biology and Biotechnology, University of Münster, Schlossplatz 8, Germany

^3^ Advanced Biomaterials, Nano-Immune Engineering, and Regenerative Nano-Medicine Program

Canada-Italy Tissue Engineering Laboratory (CITEL), ICS, Saint Boniface Albrechtsen Research, Rady Faculty of Health Sciences, University of Manitoba, Winnipeg, R2H 2A6, Manitoba, Canada

Contact E-mail: alireza.rafieerad.formal@gmail.com

**List of Supplementary Materials:**

1. **Supplementary Table S1:** A summary of the most available literature on the preparation and/or application of conventional chirality-induced nano/materials.
2. **Supplementary Table S2:** Sample labeling of the Ti_3_C_2_T_x_ MXene-based nanosheets before and after the applied surface functionalization and chirality modification.
3. **Supplementary Figure S1:** Schematic illustration of surface oxidation, hydrolysis, carboxyl-group functionalization, and induced chirality in pristine Ti_3_C_2_T_x_ MXene in water through the EDC/NHS crosslinking method.
4. **Supplementary Figure S2:** TEM morphology characterization of P1D chiral MXene aqueous colloids.
5. **Supplementary Figure S3:** TEM morphology characterization of P1L chiral MXene aqueous colloids.
6. **Supplementary Figure S4:** TEM morphology characterization of B0.15D and B0.15L chiral MXene aqueous colloids.
7. **Supplementary Figure S5:** Digital camera imaging of long-term colloidal dispersibility and stability of these carboxyl-functionalized MXene and surface modified chiral MXene-based heterostructured compared to pristine Ti_3_C_2_T_x_ MXene-based nanosheets (after around 30 days).
8. **Supplementary Figure S6:** TEM morphology of the centrifuged P0.15D and P0.15L aqueous colloids to feasibly separate the dimensionless chiral Ti_3_C_2_T_x_ MXene quantum dots and surface titanium oxides from their parent MXene nanosheets.
9. **Supplementary Figure S7:** UV-Vis optical surface absorption and digital camera imaging of long-term colloidal dispersibility of D-/L-handed chiral MXene derived quantum dots.
10. **Supplementary Table S3:** XPS atomic percentage of the pristine Ti_3_C_2_T_x_ MXene nanosheets before and after carboxyl-functionalization/chirality induction with citric acid monohydrate and D-/ or L-cysteine.
11. **Supplementary Figure S8: XPS Al 2p narrow-scan spectra of the pristine** Ti_3_C_2_T_x_ MXene **and the chiral MXene colloids P0.15D and P1L aqueous colloid.**
12. **Supplementary Figure S9:** FTIR analyses and characterization of surface functional group in the structure of pristine Ti_3_C_2_T_x_ nanosheets, carboxyl-functionalized MXene and the P1L, PlD, B0.15D, and P0.15D chiral MXene queous colloids, centrifuged and dried on sample holders for these measurements.
13. **Supplementary Figure S10:** Raman spectroscopy of the pristine Ti3C2Tx MXene nanosheets and left-/right-handed chiral-modified MXene aqueous colloids (P1D, P1L, B0.15D, P0.15D).
14. **Supplementary Figure S11:** The UV-Vis optical surface absorption analyses of as-prepared D-/L-chiral MXene hetero-structured colloids and assessment of their long-term stability in aqueous media.
15. **Supplementary Figure S12:** Assessment of phyto-compatibility of these chiral MXene aqueous colloids with *Arabidopsis thaliana* Col-0 seedlings germination and maturation.
16. **Supplementary Figure S13:** *In-planta* qualitative short-term biocompatibility and the direct nano-micro interaction of the B0.15D aqueous collides after around 2hours of foliar spraying on mature *Arabidopsis thaliana* plants.
17. **Supplementary Figure S14:** The biostimulant activity of a commercially available chitosan substance (661-Cl, DA≈20%) at different short- to mid-term time points.
18. **Supplementary Figure S15:** Qualitative monitoring of the natural growth and aging process of mature *Arabidopsis thaliana* plants inside a standard climate chamber.
19. **Supplementary Figure S16:** In-planta qualitative mid- and long-term biocompatibility of the B0.15D aqueous collides.
20. **Supplementary Figure S17:** Qualitative monitoring of the natural growth and aging process of mature *Arabidopsis thaliana* plants under standard greenhouse conditions.
21. **Supplementary Figure SI8:** Digital imaging Assessment of the seed-to-seedling transition and bioactivity of the *Arabidopsis thaliana* Col-0 with the B0.15D aqueous colloids with their biostimulant impact on enhancing seed sprouting, seedling germination, and growth.
22. **Supplementary Figure S19:** In-planta mid- and long-term drought-resistance bioactivity impact of the B0.15D aqueous collides in *Arabidopsis thaliana* inside climate chamber.
23. **Supplementary Figure S20:** The bioactivity impact of a commercially available chitosan substance (661-Cl: D_A_~20%) on enhancing seedlings tolerance to salt stress at different short- to mid-term time points.

**Supplementary Table S1:** A summary of the most available literature on the preparation and/or application of conventional chirality-induced nano/materials.

| **Synthetic Approaches** | **Method** | **Nano/Quantum Type(s)/Source(s)** | **Chiral Stimulus** | **Application(s)** | **Suppl. Ref(s)** |
| --- | --- | --- | --- | --- | --- |
| Post-functionalization with chiral ligand | EDC/NHS | Graphene nanosheets/  quantum dot | Cysteine | Optoelectronic devices | 1 |
| Post-functionalization with chiral ligand | Surface attachment | Graphene nanosheets | Amino acids / metal organic framework | Optical enantiomer separation | 2 |
| Post-functionalization with chiral ligand | EDC/NHS | Carbon nanofiber | L/D-Cysteine | Drug loading enhancement | 3 |
| Wet chemistry assembly/post- functionalization | Integration/ surface binding | Chiral metal-organic frameworks MOFs | Chiral ligands | Photonic and optoelectronics | 4 |
| Post-functionalization with chiral ligand | Amide bonds | Citric acid | Tyrosine | Electrochemical glucose sensor | 5,6 |
| Post-functionalization with chiral ligand | EDC/NHS | Sucrose | Cysteine | Cell viability | 7 |
| Post-functionalization with chiral ligand | EDC/NHS | Citric acid | L-Cysteine | Enantiomeric discrimination | 8 |
| Post-functionalization with chiral ligand | Esteriﬁcation | Graphite | 2-phenyl-1-propanol | N.A | 9 |
| One-pot strategies | Microwave | Benzoquinone |  | Moisture sensing | 10 |
| One-pot strategies | Hydrothermal | Cysteine |  | Cellular metabolism | 11 |
| One-pot strategies | Hydrothermal | Cysteine |  | Drug delivery | 12 |
| One-pot strategies | Electrolysis | Glutamine |  | Inhibited enzyme activity | 13 |
| One-pot strategies | Electrolysis | Cysteine |  | Enzyme catalysis | 14 |
| One-pot strategies |  | Glucose | Glucose | Electrocatalysis | 15 |
| One-pot strategies |  | Citric acid | Cysteine | Bio-imaging | 16 |
| One-pot strategies |  | Citric acid | Cysteine | Plant growth | 17 |
| One-pot strategies |  | Citric acid | L-aspartic acid | Sn^2+^ & L-lys sensing | 18 |
| One-pot strategies |  | Citric acid | Glutamine | Antibacterial & Cancer therapy | 19,20 |
| One-pot strategies |  | Lysine | Lysine | Drug loading/delivery | 21,22 |
| One-pot strategies |  | O-phenylenediamine |  | Sensing & water detection | 23 |
| One-pot strategies |  | N-methyl-1,2-benzenediamine dihydrochloride | L-tryptotophan | Enantiomeric discrimination | 24 |
| One-pot strategies |  |  | Arginine/ Histidine | N.A | 6 |
| Self-assembly approaches |  | Carbon dots | Cellulose nanocrystal | Bio-imaging | 25 |
| Self-assembly approaches |  | Carbon dots | Chiral helical/ solvent | Imaging | 26,27 |
| Self-assembly approaches |  | Carbon dots | Polyacetylene | N.A | 6 |
| Self-assembly approaches |  | Carbon dots | Liquid cholesteric crystal | Circle polarized phosphorescent imaging | 28 |
| Self-assembly/post-functionalization |  | Chiral Materials | Amino acid / chiral active compounds | Pharmacology, pathology & bio-interactions | 29 |
| Assembly / post-functionalization / wet chemistry |  | Inorganic chiral nanomaterials | chiral active Substances / ligands | Bio-sensing, imaging, disease diagnosis | 30 |
| Post-functionalization with chiral ligand | EDC/NHS | MXene nanosheets/  quantum dots/ heterostructures | L-/D-Cysteine | Improving the stability of MXene biomaterials & agricultural biostimulation | This work |

**Supplementary Table S2:** Sample labeling of the Ti_3_C_2_T_x_ MXene-based nanosheets before and after the applied surface functionalization and chirality modification.

| **No.** | **ID** | **Samples Description** | **Dimension** |
| --- | --- | --- | --- |
| 1 | MXF | Stirred and Bath-Sonicated Ti_3_C_2_T_x_ MXene Nanosheets in MilliQ Water | 2D-1D |
| 2 | B0.15 | Citric Acid-Treated (Carboxyl COOH) 0.15Mol MXene/Surface Oxides | 2D/1D |
| 3 | P0.15 | COOH-Functionalized 0.15M. MXene Sheets-Quantum Dots/Ti-Oxides | 2D-0D/1D |
| 4 | P1 | COOH-Treated 1M. MXene Nanosheets-Quantum Dots/Surface Oxides | 2D-0D/1D |
| 5 | B0.3L | Bath-Sonic Treated L-Cysteine Chiral 0.3Molar COOH-MXene/Oxides | 2D/1D |
| 6 | B0.3D | Bath-Sonic Treated D-Cysteine Chiral 0.3Molar COOH-MXene/Oxides | 2D/1D |
| 7 | B0.15L | Bath-Sonic Treated L-Cysteine Chiral 0.15Mol. COOH-MXene/Oxides | 2D/1D |
| 8 | B0.15D | Bath-Sonic Treated D-Cysteine Chiral 0.15Mol. COOH-MXene/Oxides | 2D/1D |
| 9 | P0.15L | Probe-Sonic Treated L-Cysteine Chiral 0.15Mol COOH-MXene/Oxides | 2D-0D/1D |
| 10 | P0.15D | Probe-Sonic Treated D-Cysteine Chiral 0.15Mo. COOH-MXene/Oxides | 2D-0D/1D |
| 11 | P1L | Probe-Sonic Treated L-Cysteine Chiral 1Molar COOH-MXene/Oxides | 2D-0D/1D |
| 12 | P1D | Probe-Sonic Treated D-Cysteine Chiral 1Molar COOH-MXene/Oxides | 2D-0D/1D |

**
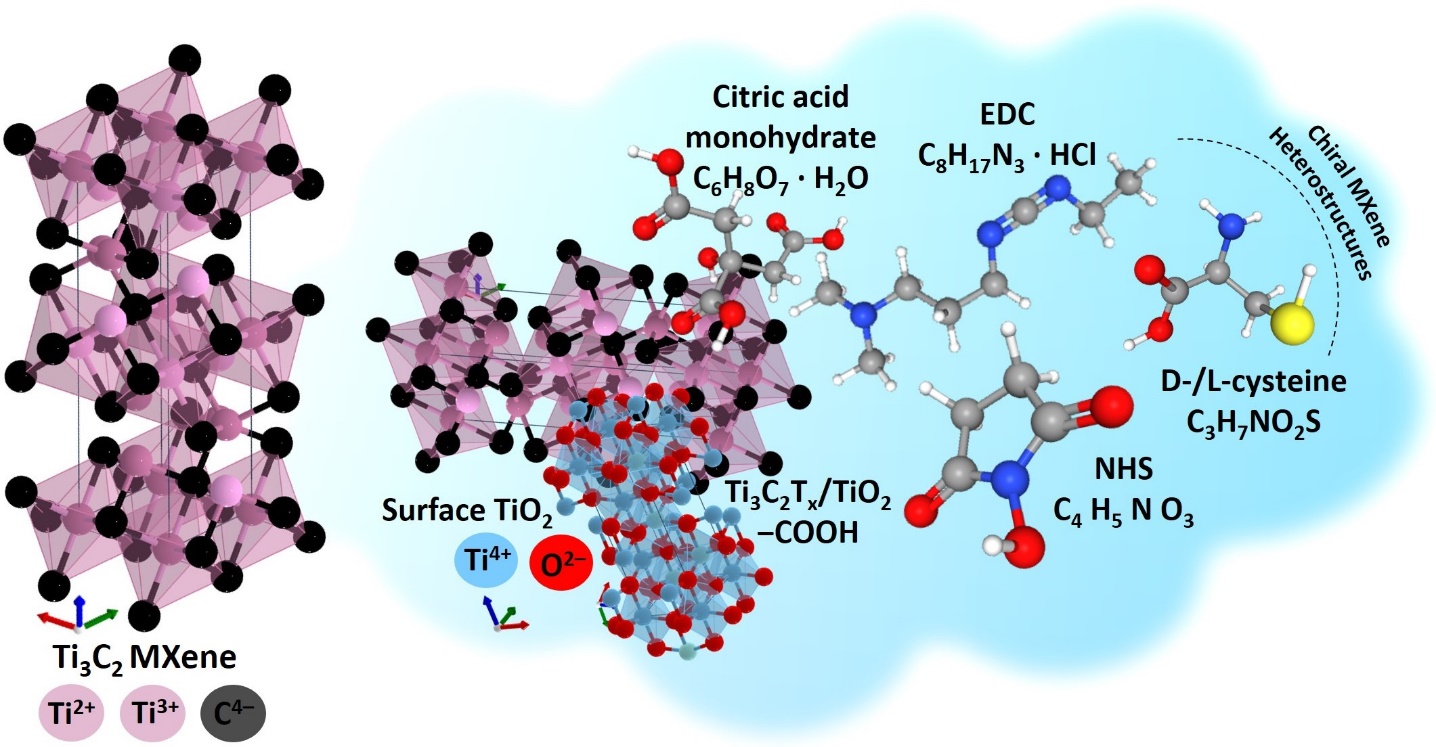
**

**Supplementary Figure S1: Schematic illustration of surface oxidation, hydrolysis, carboxyl-group functionalization, and induced chirality in pristine Ti_3_C_2_T_x_ MXene flakes dispersed in water through the EDC/NHS crosslinking method.** The carton displays the presence of MXene nanosheets and related chemicals, that reacted in the designed aqueous system via the EDC/NHS crosslinking method and in an ordered-dependent reaction procedure, as described in the manuscript. The 3D models of crystal structures of Ti_3_C_2_ MXene (code: mp-1094034, Hexagonal, *P6_3/mmc*, 194) and surface titanium oxide (code: TiO_2_, mp-390, Tetragonal, *I4_1/amd*, 141) depicted in this supplementary figure were obtained and sourced from the open-access “Materials Project” online database (<https://materialsproject.org>) with reference to the related publication associated with these models under user agreement of accepting the Creative Commons Attribution 4.0-license (A. Jain, S. P. Ong, G. Hautier, W. Chen, W. D. Richards, S. Dacek, S. Cholia, D. Gunter, D. Skinner, G. Ceder, K. A. Persson, *APL Mater.* 2013, 1, 011002 and A. Merchant, S. Batzner, S. S. Schoenholz, M. Aykol, G. Cheon, E. D. Cubuk, *Nature* 2023, 624, 80). Furthermore, the 3D crystalline models of the citric acid monohydrate, EDC, NHS, and D-/L-cysteine with their specific PubChem identifier structures were obtained/sources from “PubChem Online Database” with reference to their citing policy and related publications (Kim S, Chen J, Cheng T, Gindulyte A, He J, He S, Li Q, Shoemaker BA, Thiessen PA, Yu B, Zaslavsky L, Zhang J, Bolton EE. PubChem 2025 update. Nucleic Acids Res. 2025 Jan 6;53(D1):D1516–25. [doi:10.1093/nar/gkae1059](https://doi.org/10.1093/nar/gkae1059). PMID: [39558165](https://www.ncbi.nlm.nih.gov/pubmed/39558165) PMCID: [PMC11701573](https://www.ncbi.nlm.nih.gov/pmc/articles/PMC11701573/). PubChem [Internet]. Bethesda (MD): National Library of Medicine (US), National Center for Biotechnology Information; 2004-. PubChem Compound Summary for CID 92851, D-cysteine; [cited 2025 Jan. 12]. Available from: <https://pubchem.ncbi.nlm.nih.gov/compound/D-cysteine>. PubChem [Internet]. Bethesda (MD): National Library of Medicine (US), National Center for Biotechnology Information; 2004-. PubChem Compound Summary for CID 5862, Cysteine; [cited 2025 Jan. 12]: <https://pubchem.ncbi.nlm.nih.gov/compound/Cysteine>). The other 3D models were also obtained from the “PubChem Online Database” websites.

**
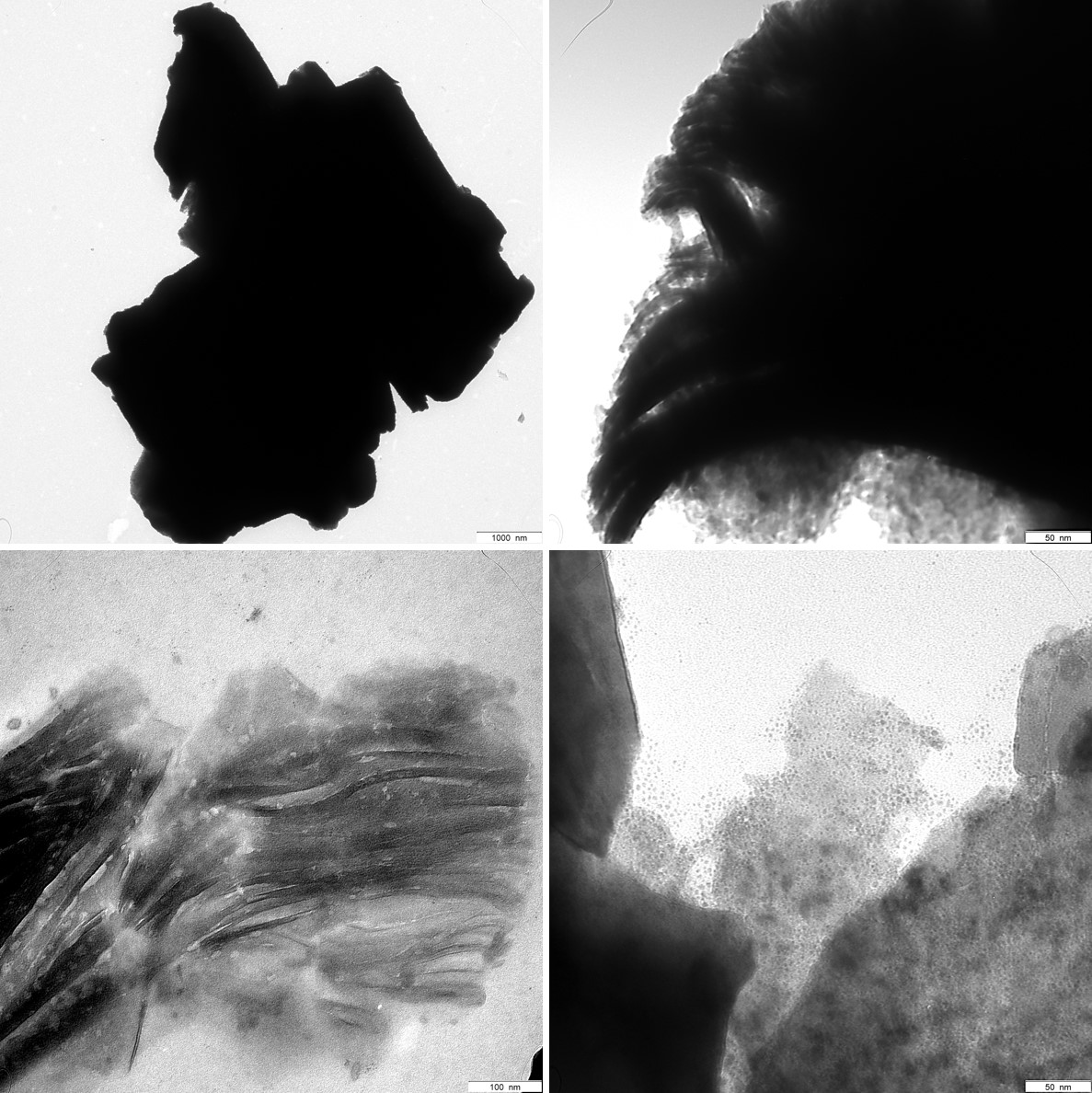
**

**Supplementary Figure S2: TEM morphology characterization of the P1D aqueous colloids.** The TEM micrographs of probe-sonication treated carboxyl functionalized Ti_3_C_2_T_x_ MXene-based nanosheets treated with D-cysteine show morphological structure of these mixed-low-dimensional 2D-0D/1D collides, including Ti_3_C_2_T_x_ MXene nanosheets, self-derived MXene quantum dots, and stable titanium oxide particles in cluster forms.

**
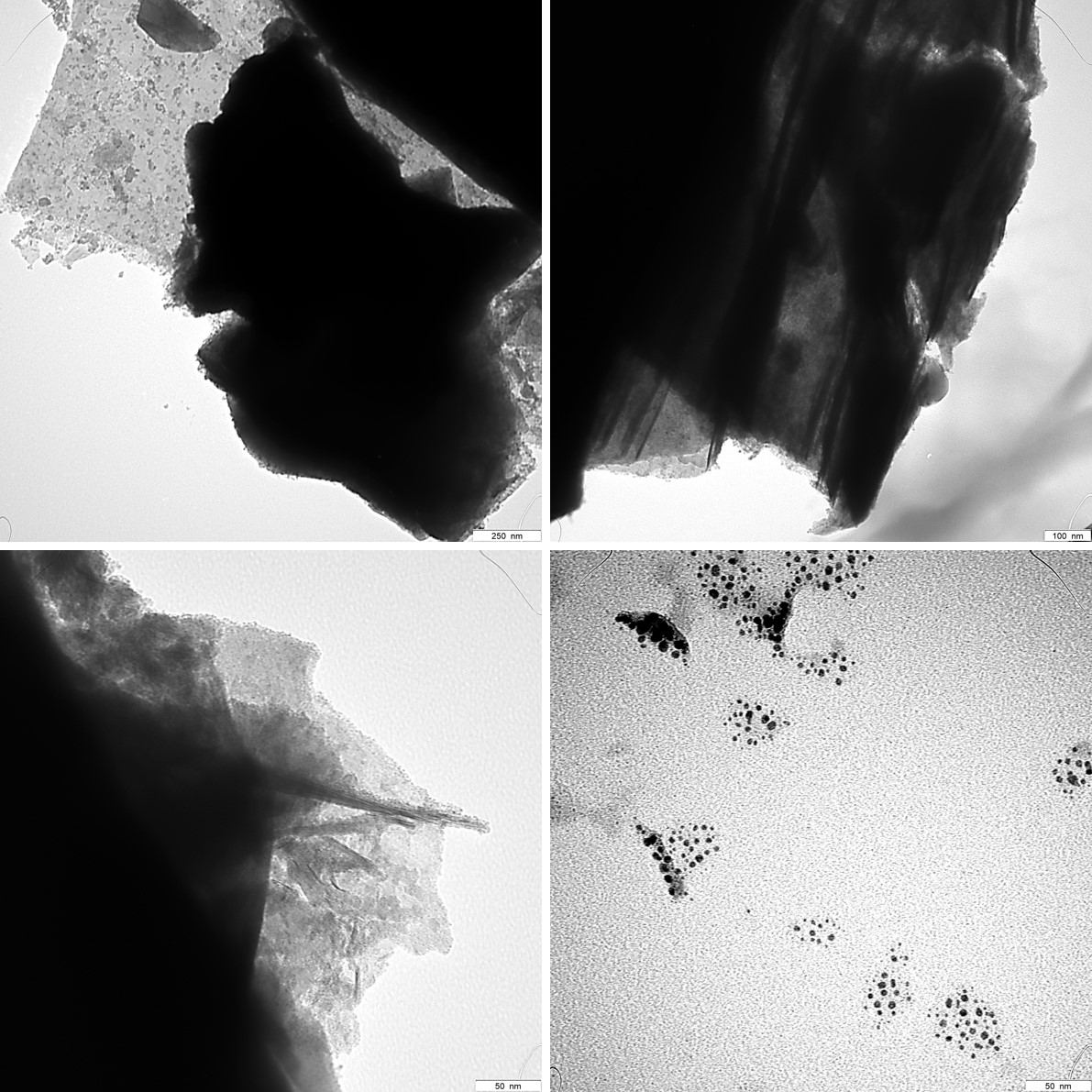
**

**Supplementary Figure S3: TEM morphology characterization of the P1L aqueous colloids.** The TEM micrographs of probe-sonication treated carboxyl functionalized Ti_3_C_2_T_x_ MXene-based nanosheets treated with L-cysteine show morphological structures of these mixed-low-dimensional 2D-0D/1D collides, including Ti_3_C_2_T_x_ MXene nanosheets, self-derived MXene quantum dots, and stable titanium oxide particles in cluster forms.

**
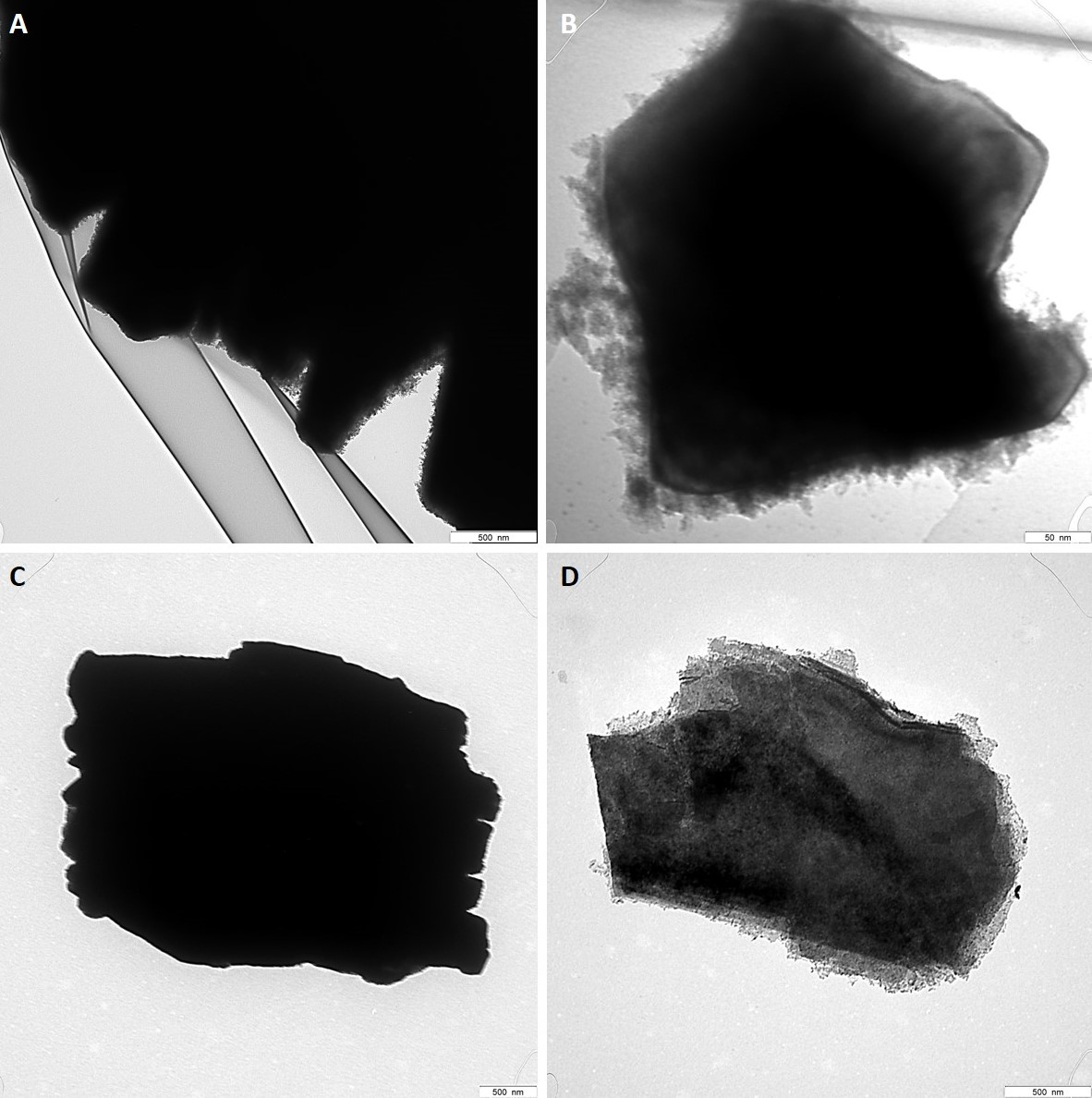
**

**Supplementary Figure S4: TEM morphology characterization of the B0.15D and B0.15L aqueous colloids.** The TEM images of bath-sonication treated carboxyl functionalized Ti_3_C_2_T_x_ MXene-based nanosheets treated with **A**,**B**, D-cysteine and **C**,**D**, L-cysteine show morphological structures of these collides, including the Ti_3_C_2_T_x_ MXene nanosheets and relative titanium oxide surface particles with some self-derived cluster of MXene-based quantum dots on the surface and between the layers of these nanosheets.

**
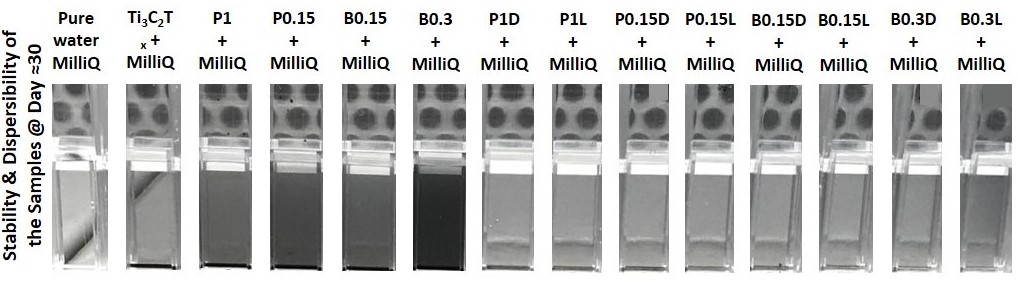
**

**Supplementary Figure S5: Digital Imaging of MilliQ water, pristine Ti_3_C_2_T_x_ MXene-based nanosheets, probe/bath treated carboxyl-functionalized MXene nanosheets, and chiral surface-modified heterostructured aqueous colloids.** Camera images show the relatively long-term colloidal dispersibility and stability of these samples compared to pristine MXene nanosheets and water. These qualitative observations on day around 30 of materials dispersion/preparation suggest the enhanced aqueous colloidal stability of these MXene nanosheets after functionalization with citric acid (carboxyl-based terminal addition) and surface modification with D- or L-cysteine in the form of chiral MXene colloids.

**
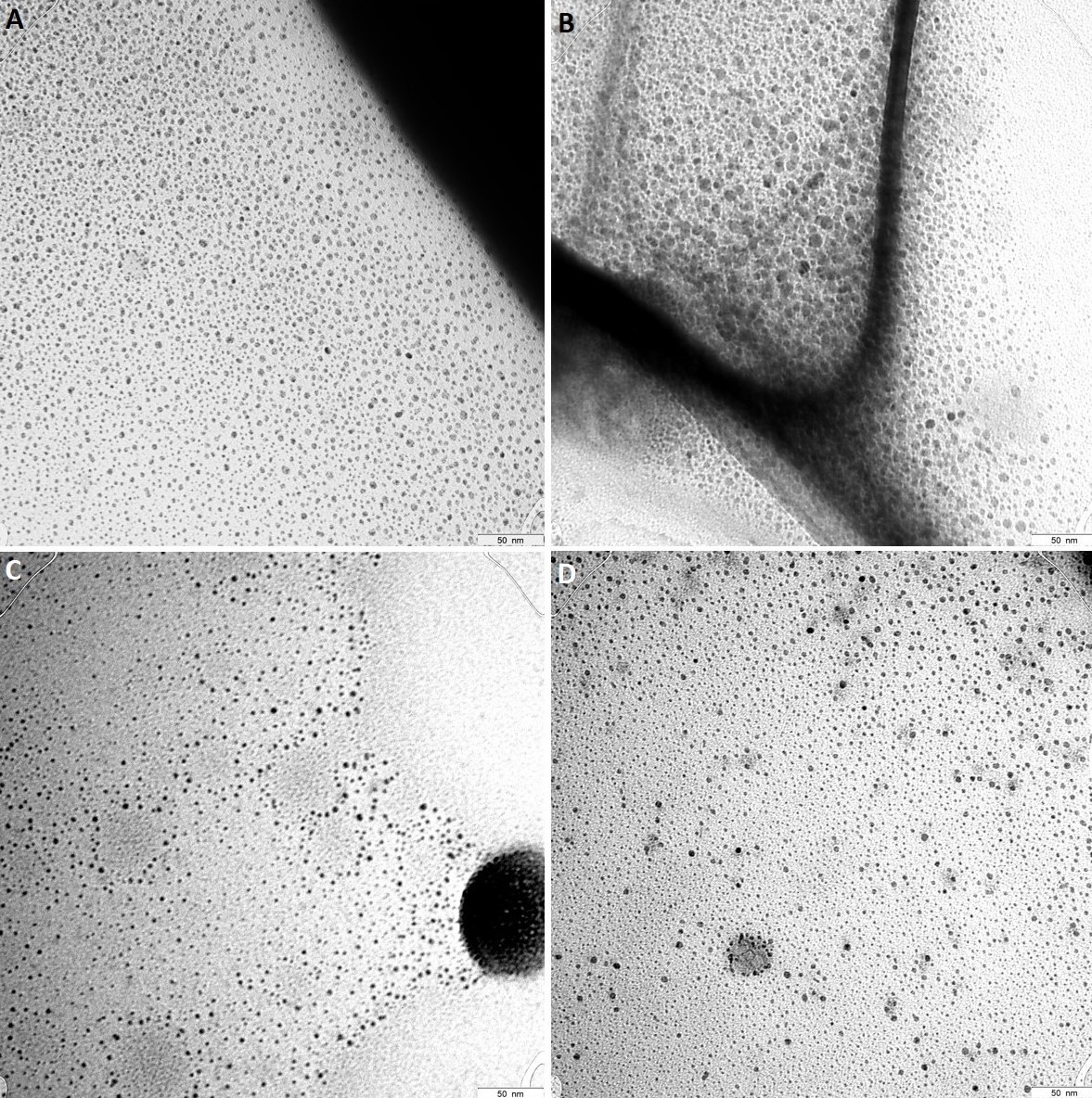
**

**Supplementary Figure S6: TEM morphology of the centrifuged P0.15D and P0.15L aqueous colloids to feasibly separate the dimensionless chiral Ti_3_C_2_T_x_ MXene quantum dots and surface titanium oxides from their parent MXene nanosheets.** The TEM micrographs of probe-sonication treated carboxyl-functionalized Ti_3_C_2_T_x_ MXene-based quantum dots chirality after chirality induction with the D- or L-cysteine. These data suggest a high capacity of the applied versatile methodology to synthesize high-quality MXene quantum dots with an average diameter of less than 10 nm without using the typical hydrothermal equipment or treatment. These quantum dots are dispersed in the aqueous mixture of the prepared 2D-0D/1D chiral MXene heterostructures beyond those that are stably attached/decorated on and between the treated MXene nanosheets; thereby, they could be separated from suspension mixtures through one cycle of spinning at a moderate rate of around 1500 rpm for five minutes.

**
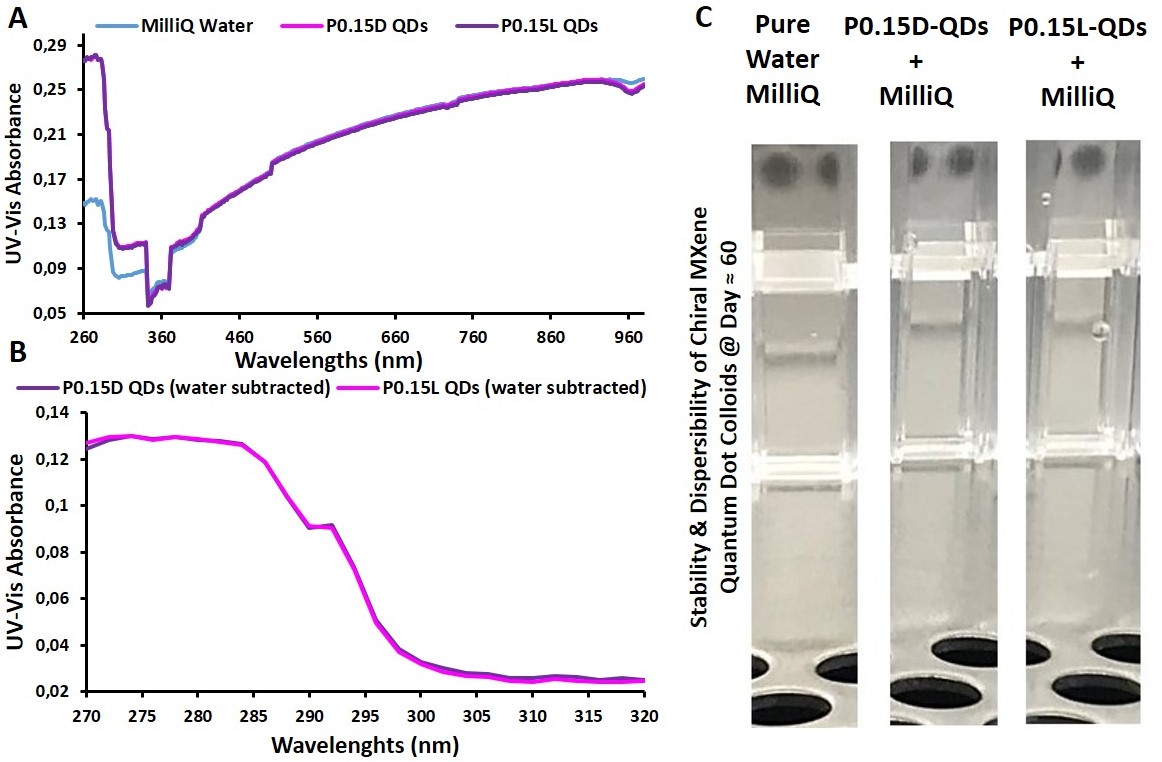
**

**Supplementary Figure S7: UV-Vis optical absorption and digital camera imaging of long-term aqueous colloidal dispersibility of optimal right-/left-handed chiral MXene derived quantum dots. A**,**B**, UV-Vis spectra of the P0.15D-QDs and P0.15L-QDs samples before and after subtracting from MilliQ water. **C**, The digital camera images of these samples compared to pure water show their relatively long-term colloidal dispersibility (around 60 days at four degrees).

**Supplementary Table S3:** The XPS atomic percentage of the pristine Ti_3_C_2_T_x_ MXene nanosheets before and after carboxyl-functionalization/chirality induction with citric acid and D- or L-cysteine.

| ***Sample IDs***  ***XPS Scans*** | **Pristine 2D Ti_3_C_2_T_x_ Nanosheets (MXF)** | **2D-0D/1D D-Chiral**  **COOH-Ti_3_C_2_T_x_ (P0.15D)** | **2D-0D/1D L-Chiral COOH-Ti_3_C_2_T_x_ (P1L)** |
| --- | --- | --- | --- |
| **C 1s %** | 60.610 | 58.8375 | 50.170 |
| **O 1s %** | 35.740 | 26.3625 | 32.550 |
| **Ti 2p %** | 3.650 | 5.525 | 8.390 |
| **N 1s %** | N.A. (~0.00) | 9.275 | 8.890 |
| **Al 2p %** | ~0.00 | ~0.00 | ~0.00 |
| *Total:* | ~100.00 | ~100.00 | ~100.00 |

**
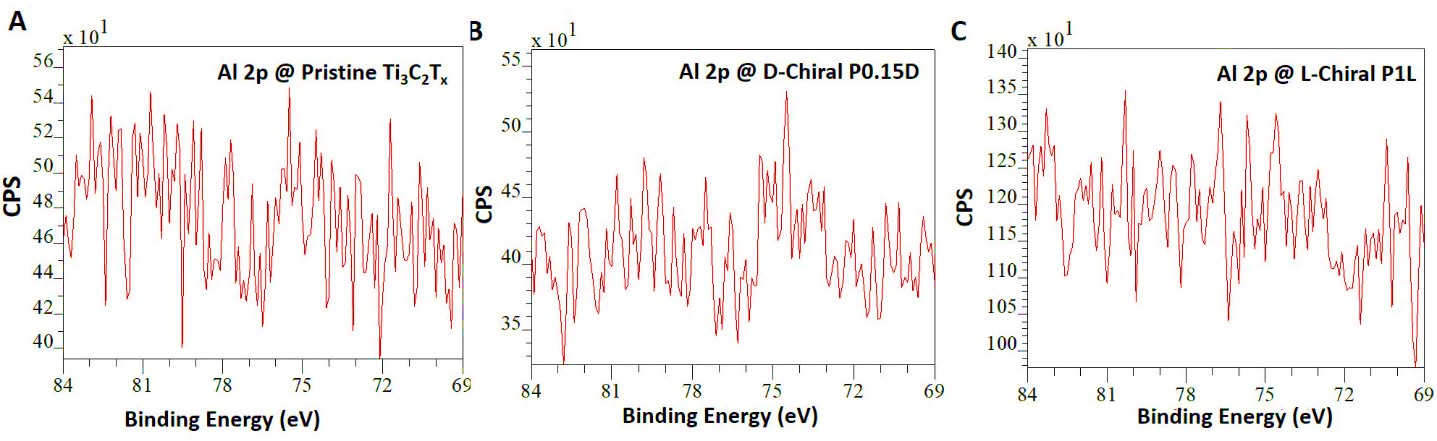
**

**Supplementary Figure S8: XPS Al 2p narrow-scan spectra of the pristine Ti3C2Tx MXene-based nanosheets and surface-modified chiral P0.15D and P1L MXene aqueous colloid. A**-**C**, The XPS narrow-scan of Al 2p of these MXenes in aqueous dispersions loaded on the XPS sample holder. This data showed a negligible amount (atomic ratio percentage) of aluminum in the surface chemistry of these MXene nanosheets. These spectra also suggest no significant differences in the aluminum compositions after the applied surface functionalization and chirality induction.

**
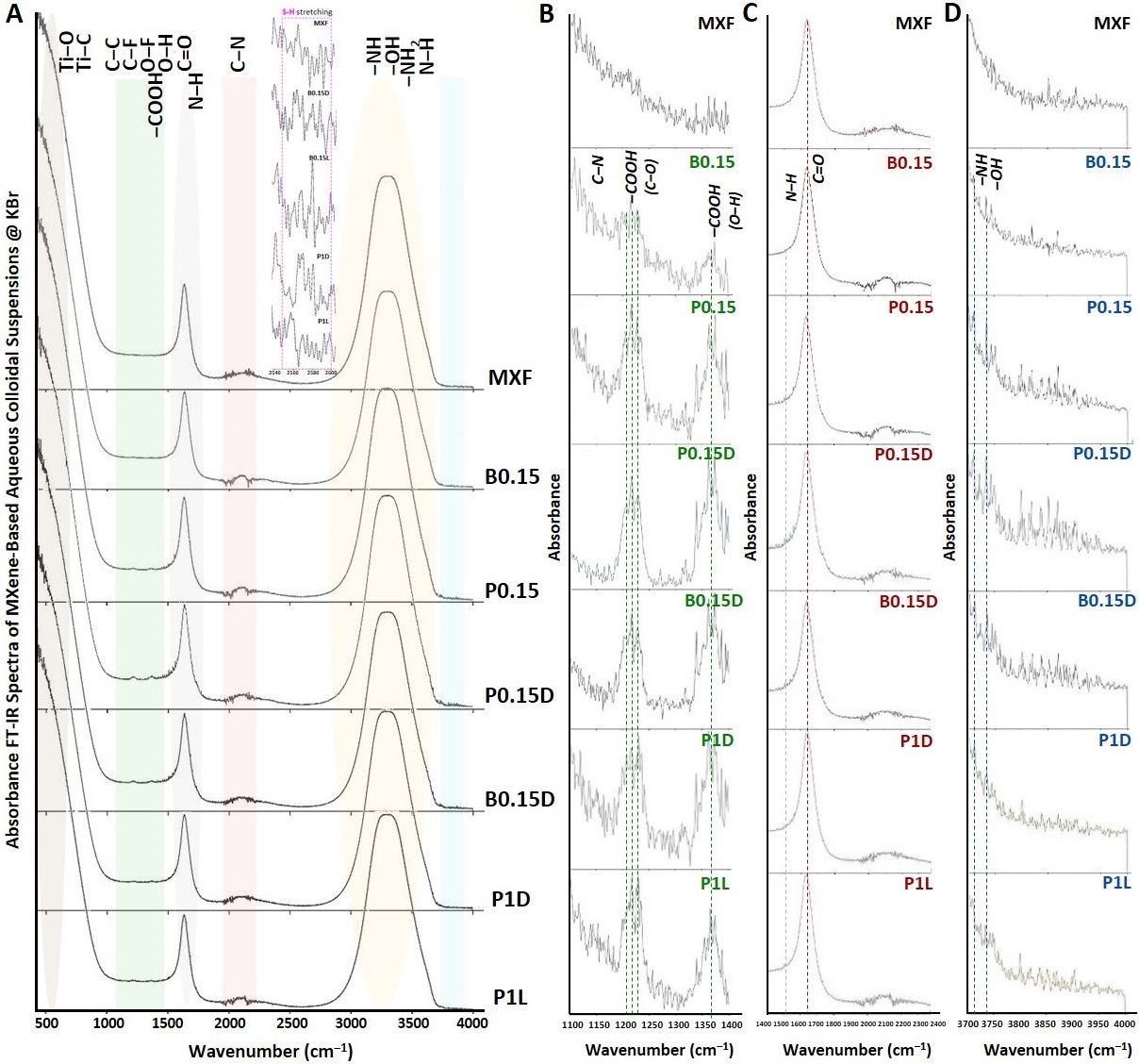
**

**Supplementary Figure S9: FTIR analyses and characterization of surface functional group in the structure of pristine Ti_3_C_2_T_x_ nanosheets, carboxyl-functionalized MXene and the P1L, PlD, B0.15D, and P0.15D aqueous colloids, centrifuged and dried on sample holders.** **A**-**D**, The FTIR absorbance spectra of these samples identified an overview of the general surface chemistry and organic/inorganic-based binding available in the structure of these samples before and after surface functionalization and chirality induction at the wavenumber in the range of 400 to 400 cm^˗1^.


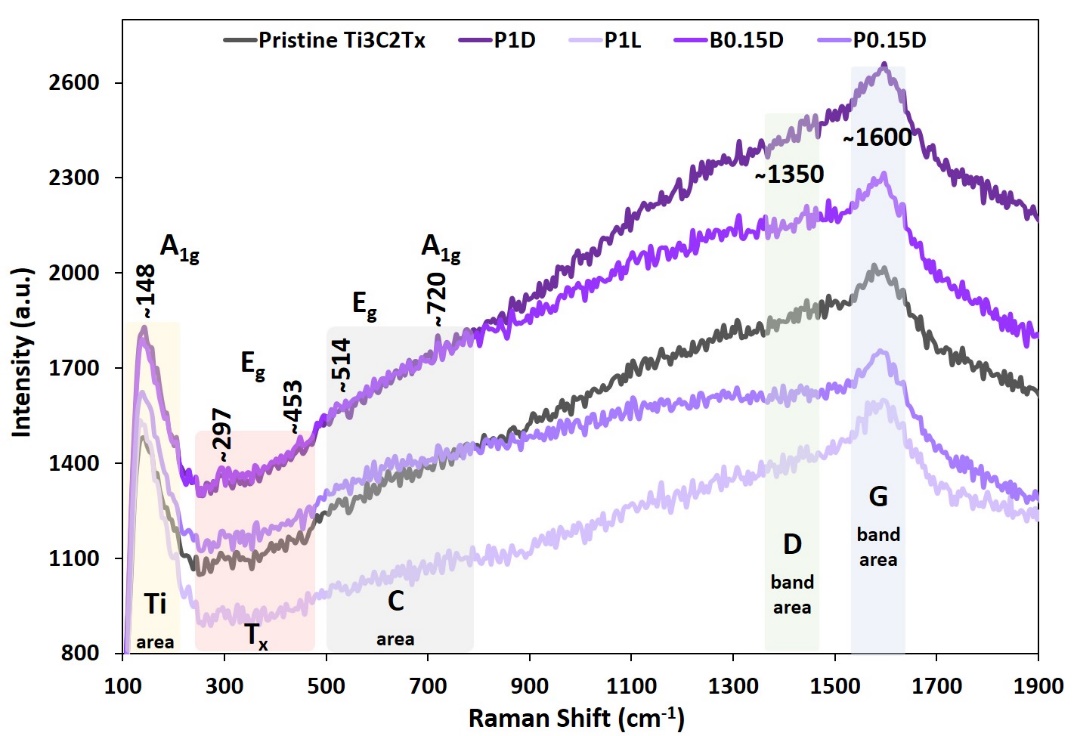


**Supplementary Figure S10: Raman spectroscopy of the pristine Ti_3_C_2_T_x_ MXene nanosheets and left-/right-handed chiral-modified MXene aqueous colloids (P1D, P1L, B0.15D, P0.15D).** The Raman spectra of these MXenes in aqueous dispersions showed the characteristic structures, chemical bindings, and functional groups available on the surface of these MXene-based materials.


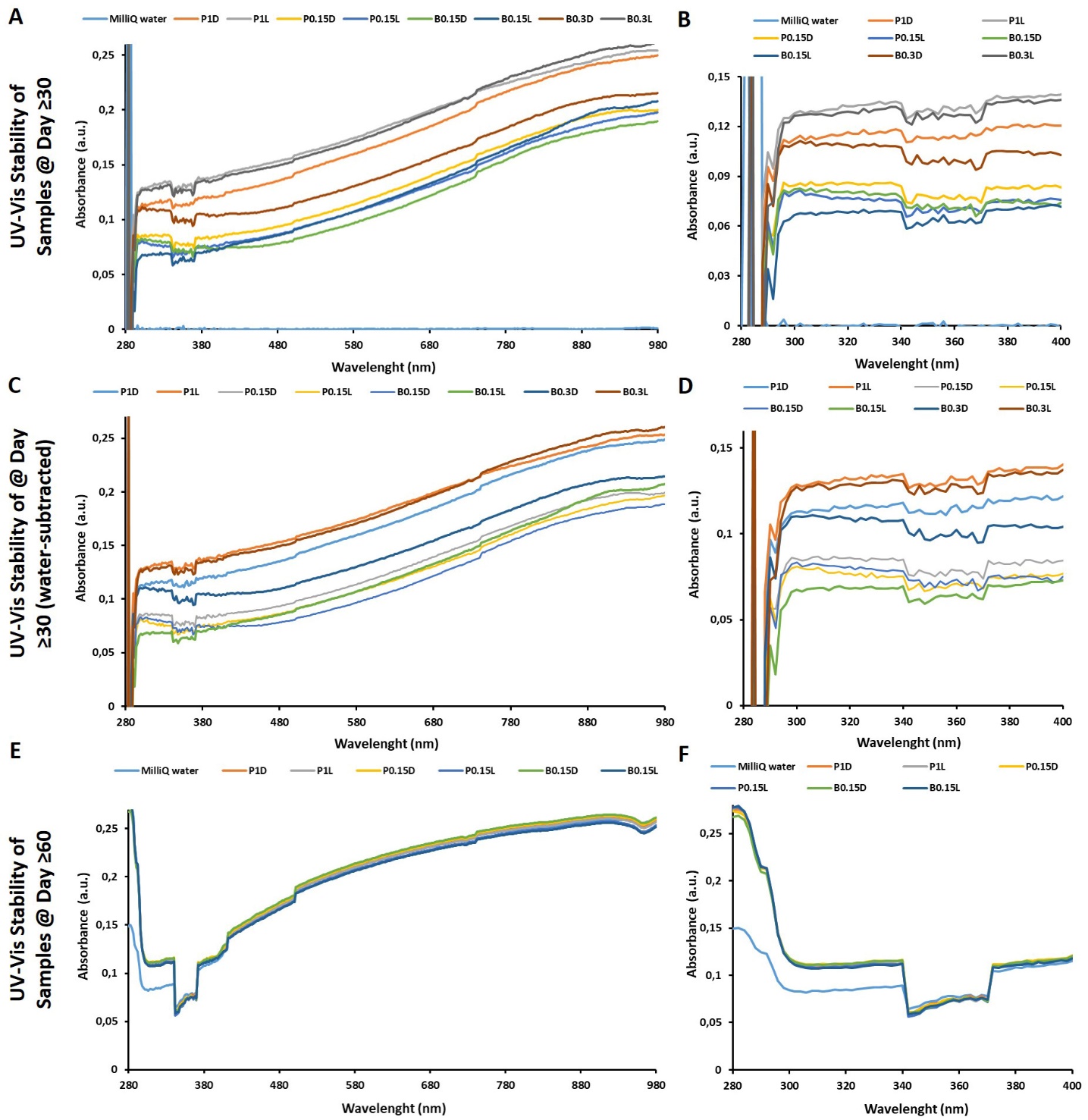


**Supplementary Figure S11: The UV-Vis optical surface absorption analyses of as-prepared D-/L-chiral MXene hetero-structured colloids and assessment of their long-term stability in aqueous media.** **A**,**B**, The UV-Vis spectra of the samples (≈1 mL) at day 30 post-preparation, before and after subtracting their absorption from the MilliQ water. **C**,**D**, Longer-term colloidal stability and resistance to excessive surface oxidation, degradation, and material decomposition in water at day around 60 at the different wavelengths ranging from 280 to 980 nm.

**
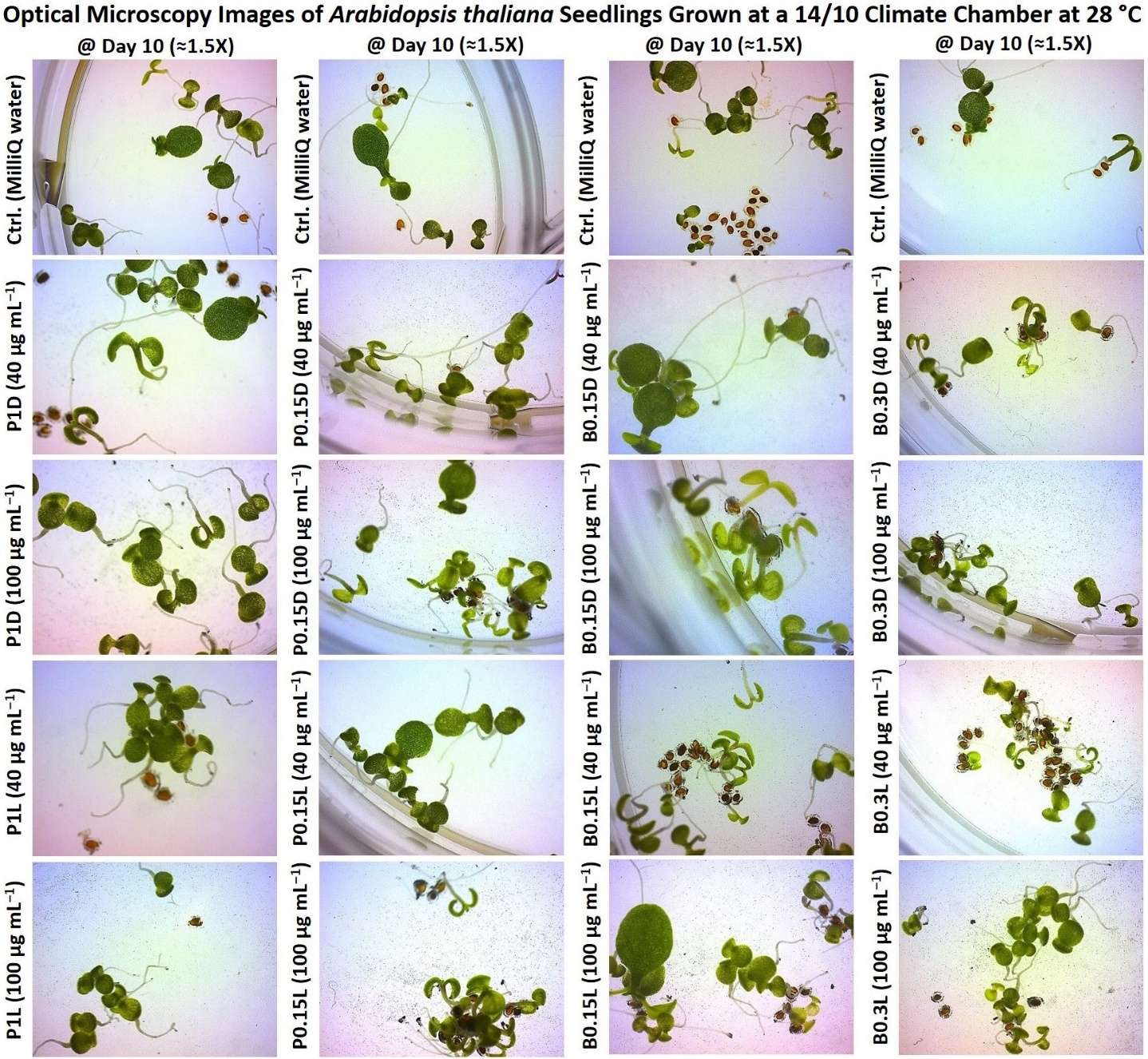
**

**Supplementary Figure S12: Assessment of phytocompatibility of these chiral MXene aqueous colloids with *Arabidopsis thaliana* Col-0 seedlings germination and maturation.** Optical microscopy at zoom magnification of 1.5X depicted their development process at day 10 post-treatment in MilliQ water-treated control and chiral B0.15D colloids at the concentrations of 40 and 100 µg mL^−1^ in a sucrose-supplemented MS medium (n=10-20). These representative bright-field images showed that the materials at both tested doses effectively promoted the maturation process of *Arabidopsis thaliana* seedlings. These microscopic observations suggest the transformation of these seeds did not impose any significant and microscope-visible adverse effects on their germination, and root/shoot development.

**
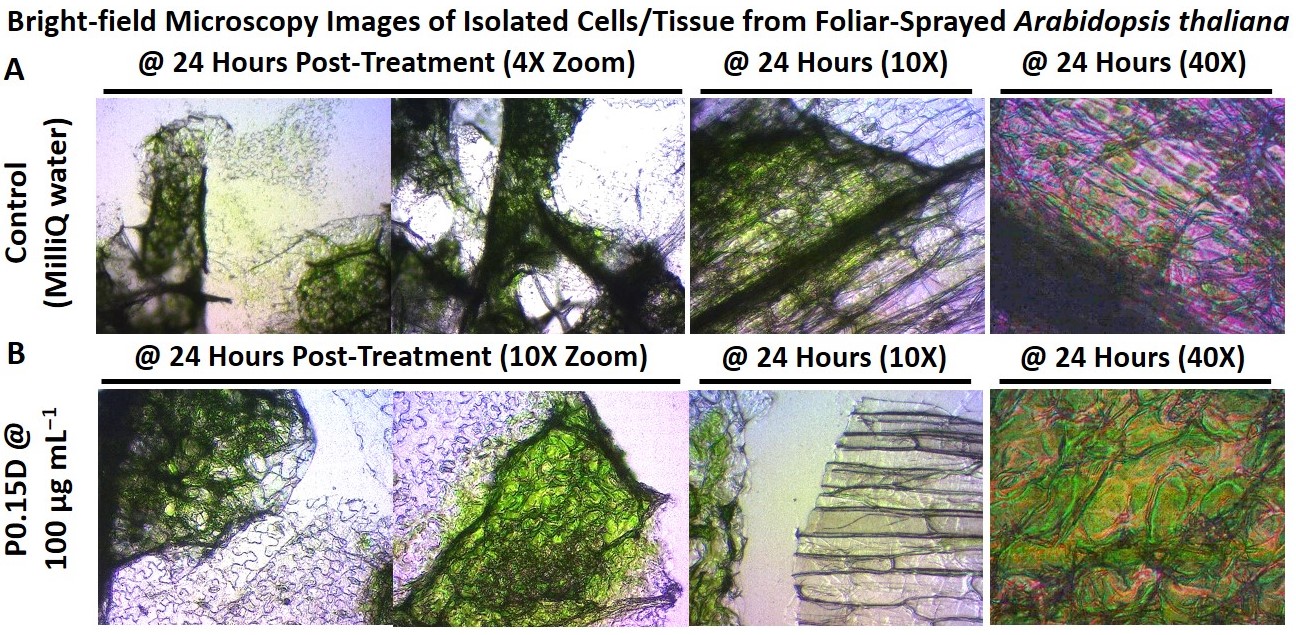
**

B

**Supplementary Figure S13: *In-planta* qualitative short-term biocompatibility and the direct nano-micro interaction of the B0.15D aqueous collides after around 2hours of foliar spraying on mature *Arabidopsis thaliana* plants.** The optical microscopy images of the isolated cells and root tissue depicted no significant changes in the structure and morphology of these plant residues compared to the water-sprayed plant, suggesting the high phyto-compatibility of these optimal chiral MXene colloids with the tested plant.

**
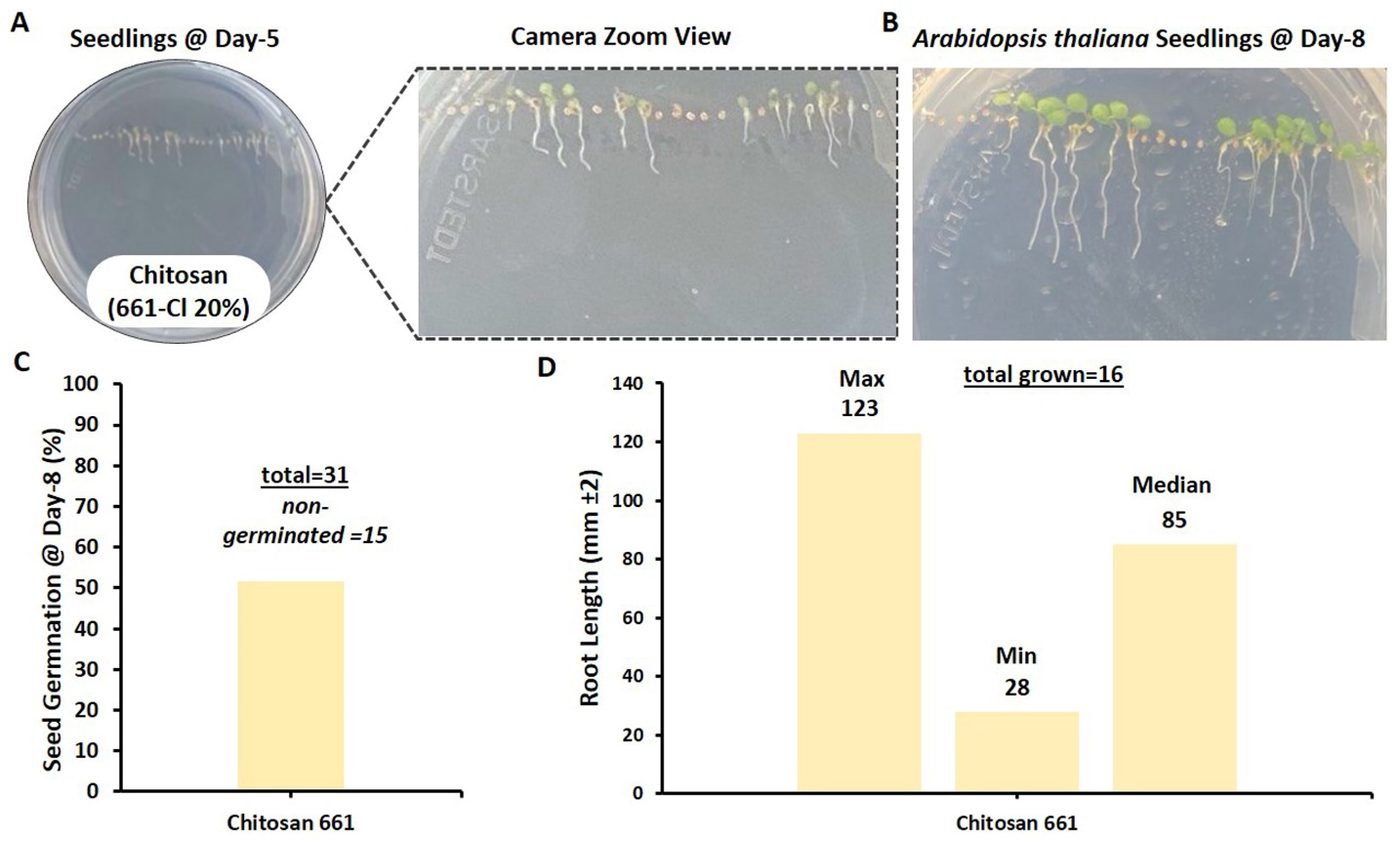
**

**Supplementary Figure S14: The biostimulant activity of a commercially available chitosan substance (661-Cl: D_A_~20%) at different short- to mid-term time points.** **A**-**D**, The seeds of *Arabidopsis thaliana* Col-0 were line-cultured in solidified media with and without this chitosan solution at a similar concentration of 88 µg mL^−1^. The camera images and their related seed germination and root length analysis were compared with the biostimulant impact of chiral MXene colloids on enhancing the seedlings' maturation at day 8 post-treatment. The ImageJ was used to measure the camera images of the roots using a similar trend with control and chiral MXene groups. The measured max, min, and median of the roots suggest the bioactivity of this chitosan, however, our data show superior biostimulant properties of B0.15D samples compared to this chitosan and control samples (n: is equal to at least twenty-five per sample).

.

**
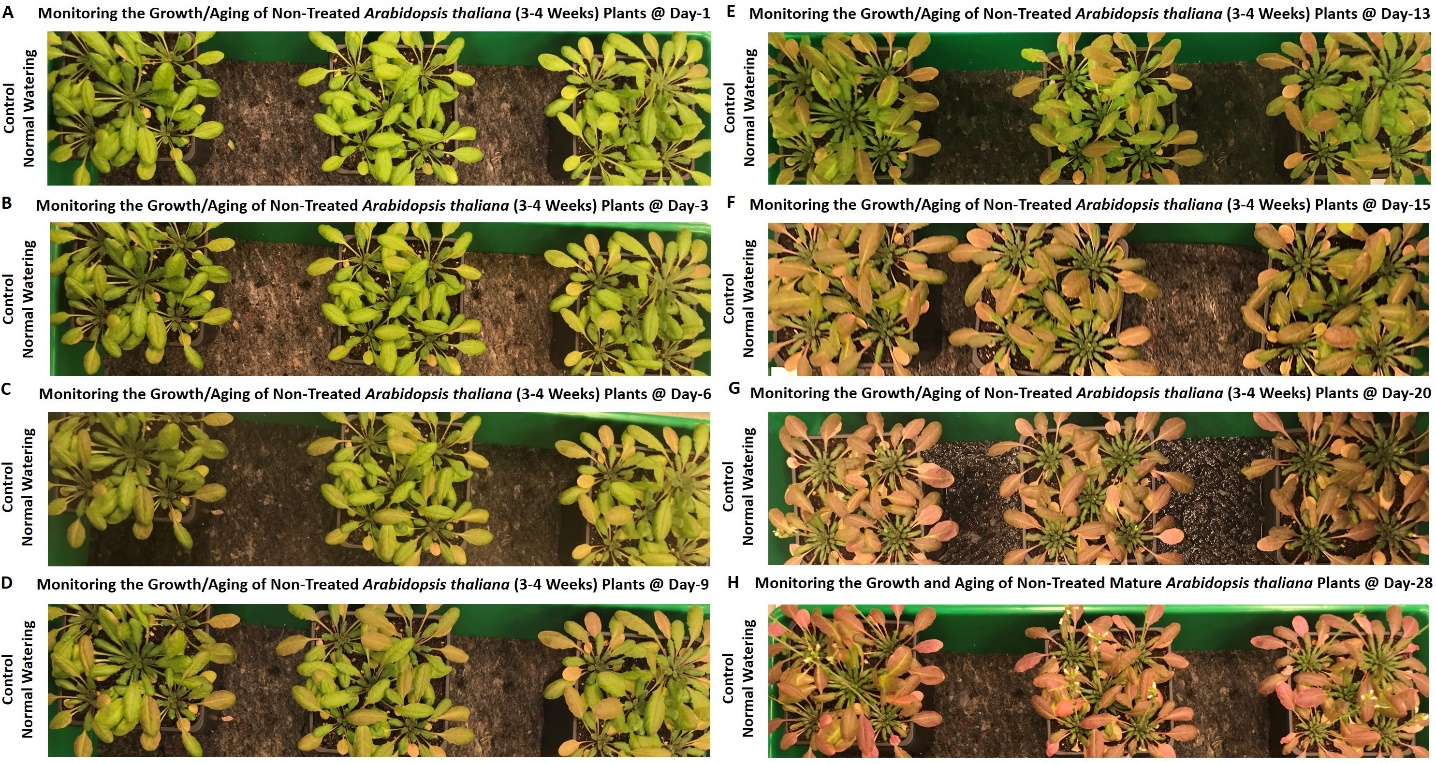
**

**Supplementary Figure S15: Qualitative monitoring of the natural growth and aging process of mature *Arabidopsis thaliana* plants inside a standard climate chamber.** Plants have been normally watered and their growth was qualitatively monitored at different time points of day-1, 3, 6, 9, 10, 13, 15, 20, and 28. As expected, no significant adverse effect was observed and the plant follow their natural aging and flowering processes.

**
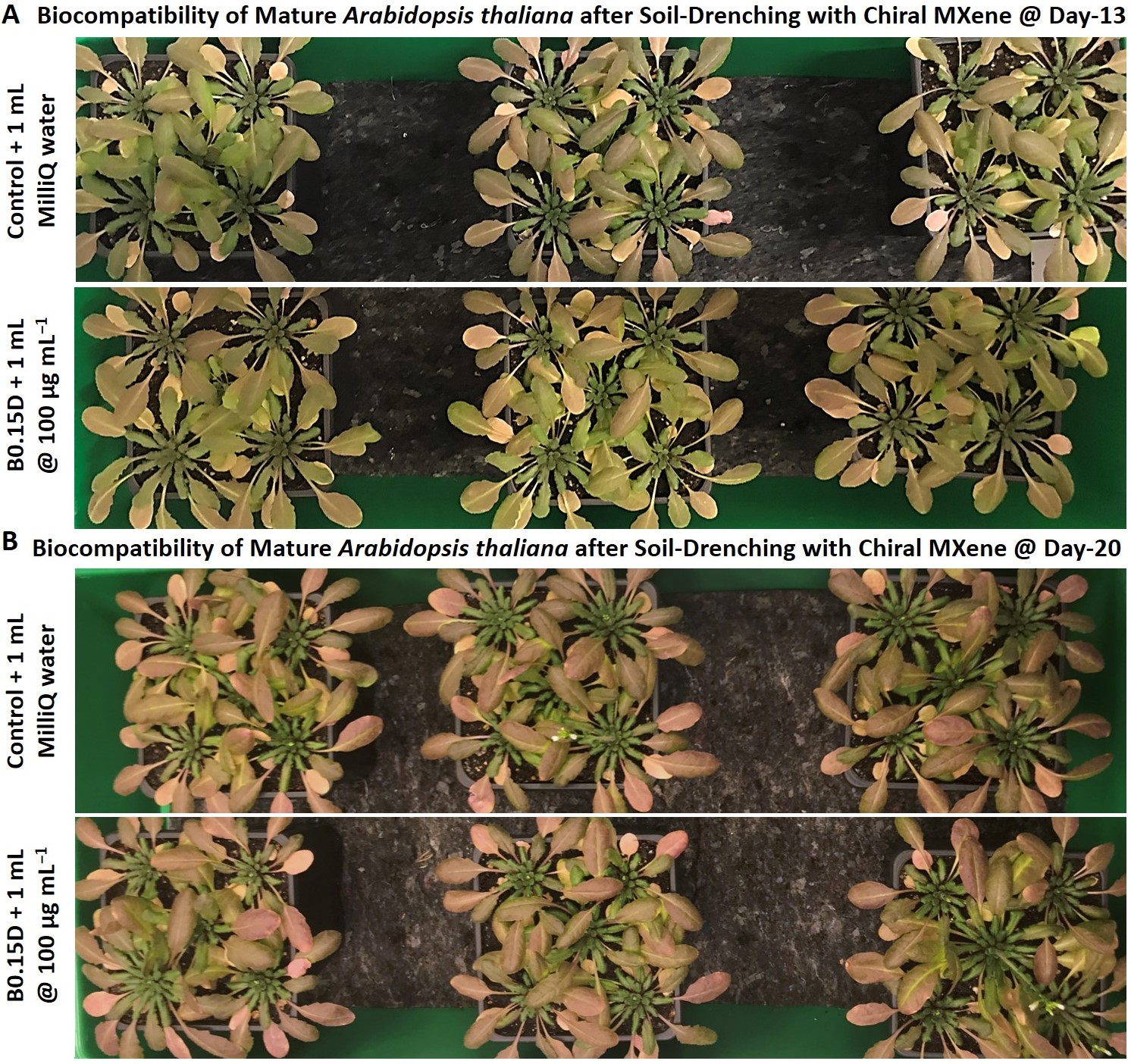
**

**Supplementary Figure S16: *In-planta* qualitative mid- and long-term biocompatibility of the B0.15D aqueous collides.** Assessment of biocompatibility and direct interaction of these colloids at the concentration of 100 µg mL^−1^ with young and mature *Arabidopsis thaliana* (3-4 weeks old) and their qualitative impacts on these plants’ overall growth, and physiological conditions at different time points of day-13 and 20 post-soil-drenching treatment applied on the entire leaf/shoot parts (n=3 and each pot represents five individual *Arabidopsis thaliana* plants). Our longer-term phyto-compatibility assessments of these colloids showed no significant adverse effects of the plant’s natural growth, aging, flowering, and overall visible physiological conditions compared to the control plants.

**
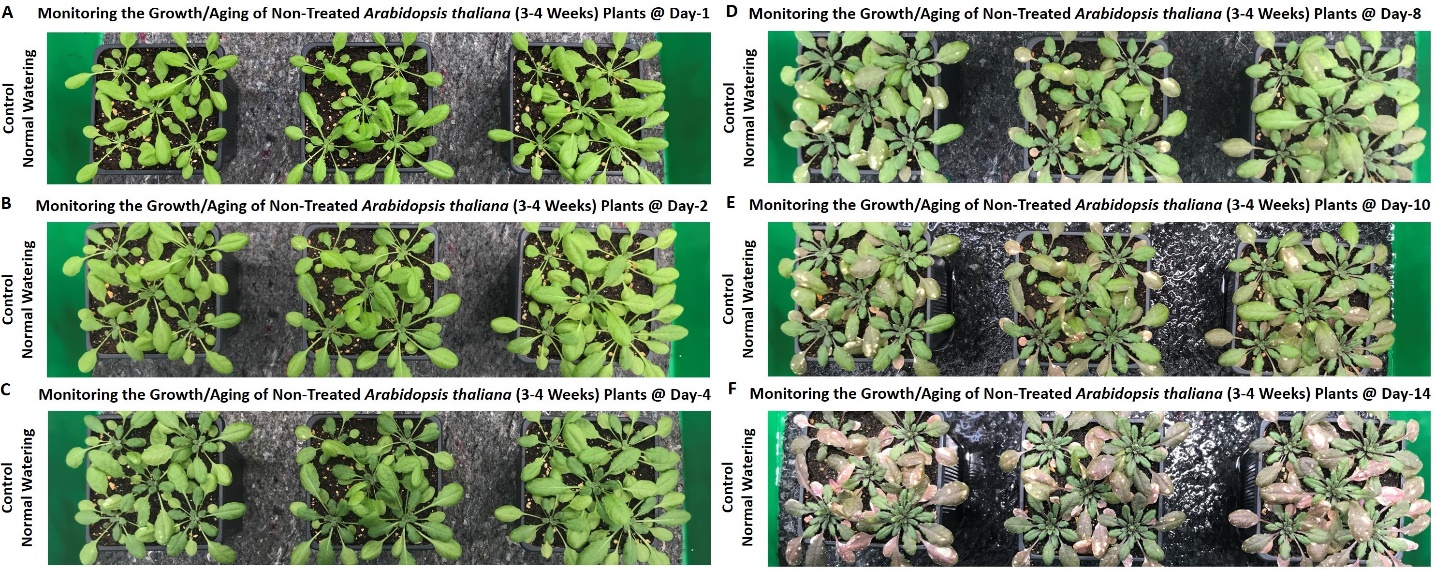
**

**Supplementary Figure S17: Qualitative monitoring of the natural growth and aging process of mature *Arabidopsis thaliana* plants under standard greenhouse conditions.** Plants have been normally watered and their growth was qualitatively monitored at different time points of day-1, 2, 4, 8, 10, and 14.

**
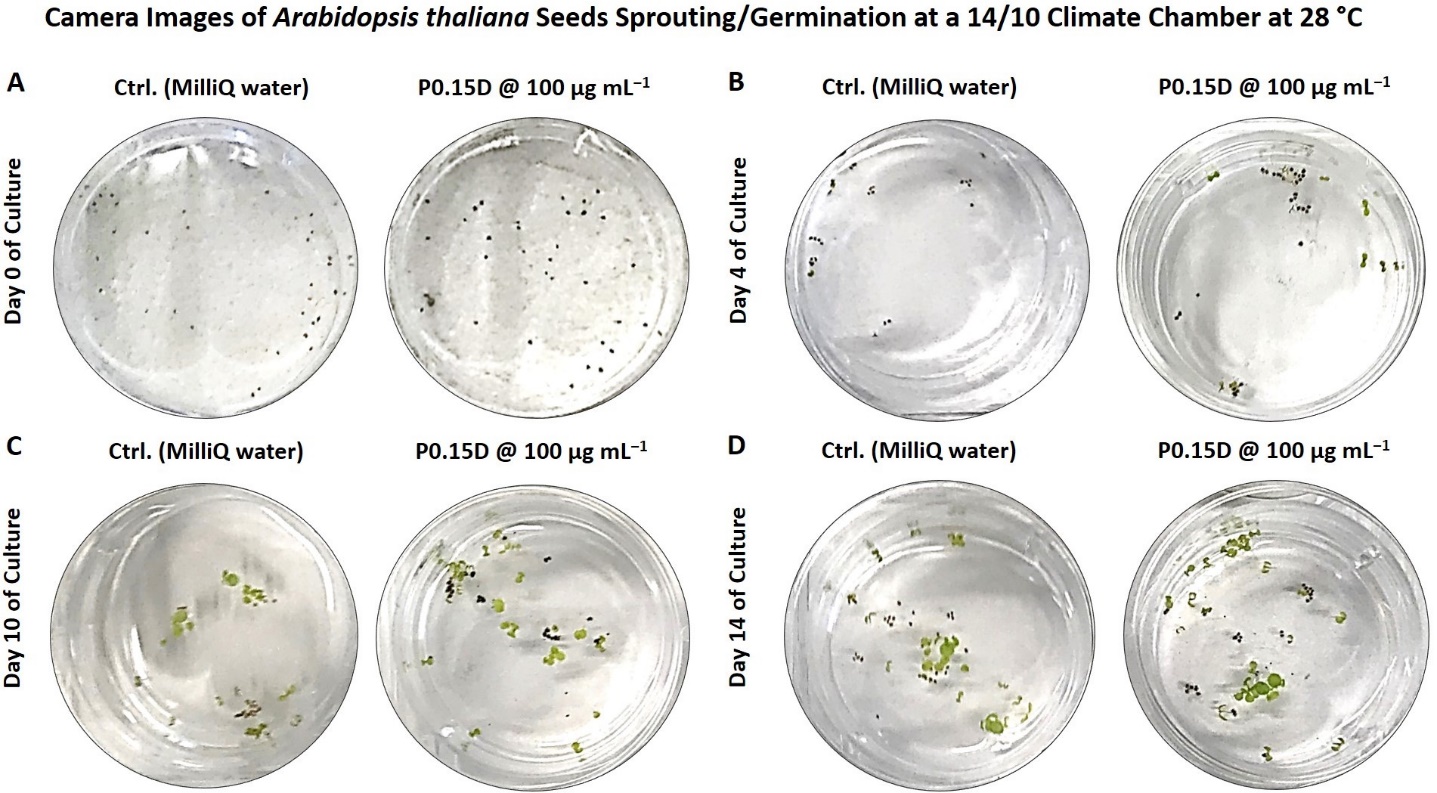
**

**Supplementary Figure SI8: Digital imaging Assessment of the seed-to-seedling transition and bioactivity of the *Arabidopsis thaliana* Col-0 with the B0.15D aqueous colloids with their biostimulant impact on enhancing seed sprouting, seedling germination, and growth.** The camera images illustrate that the applied seed-coating with these optimal chiral MXene colloids at the concentration of 100 µg mL^−1^ was not only imposed any adverse impacts but also promoted the transition and maturation stages of these seeds over the first two weeks of culture inside the climate chamber and in MS-media (n=10 to 20).

**
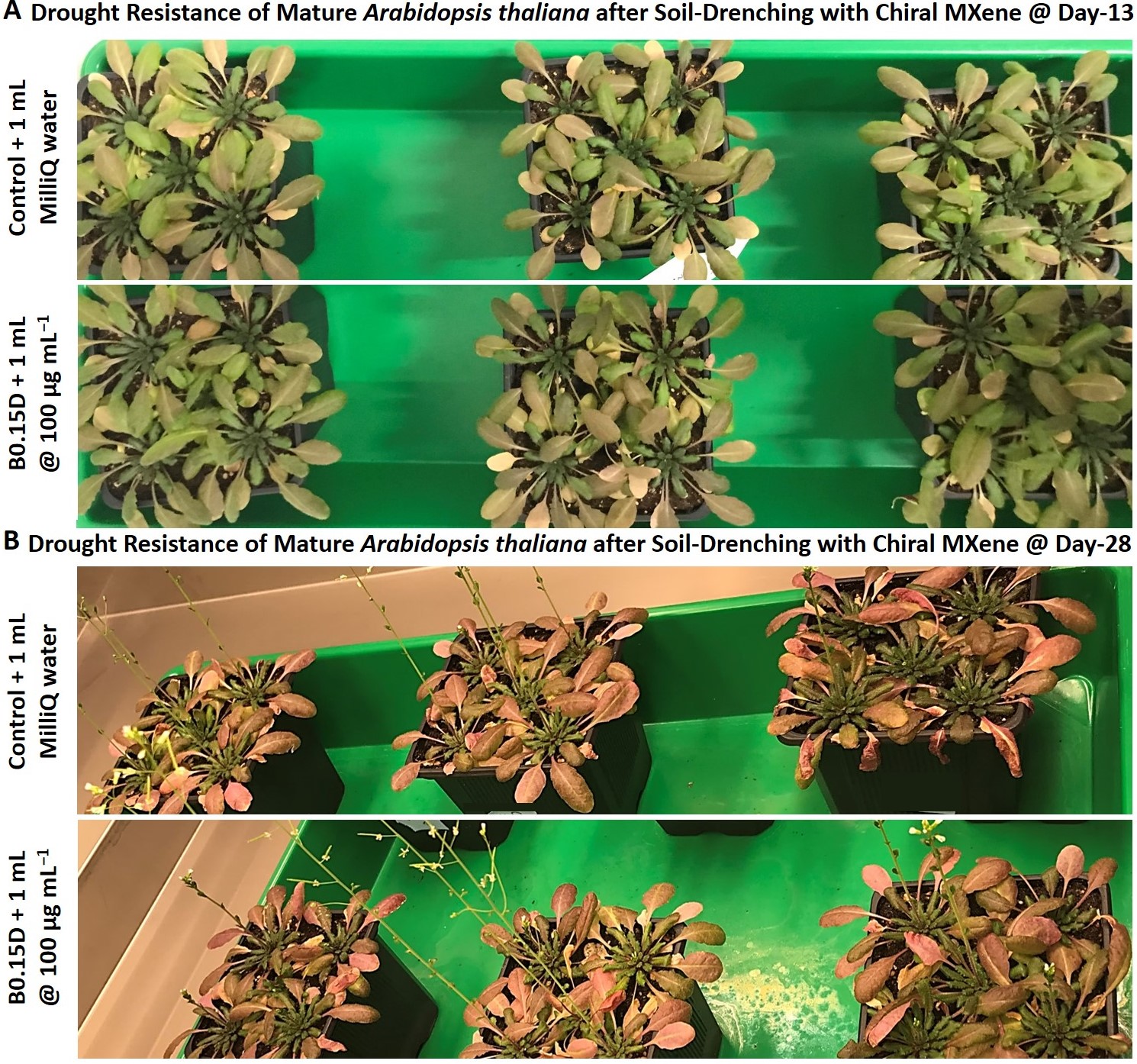
**

**Supplementary Figure S19: *In-planta* mid- and long-term drought-resistance bioactivity impact of the B0.15D aqueous collides in *Arabidopsis thaliana* inside climate chamber.** Assessment of the plant’s tolerance to continues extreme abiotic drought stress conditions at day 13 and 28 post soil-drenching treatment and maintaining under the standard climate chamber conditions. Interaction of these chiral MXene colloids at the concentration of 100 µg mL^−1^ with young and mature *Arabidopsis thaliana* (3-4 weeks old) was qualitatively monitored compared to control plants, and their impacts on these plants’ overall growth, flowering, and aging were observed by digital imaging.


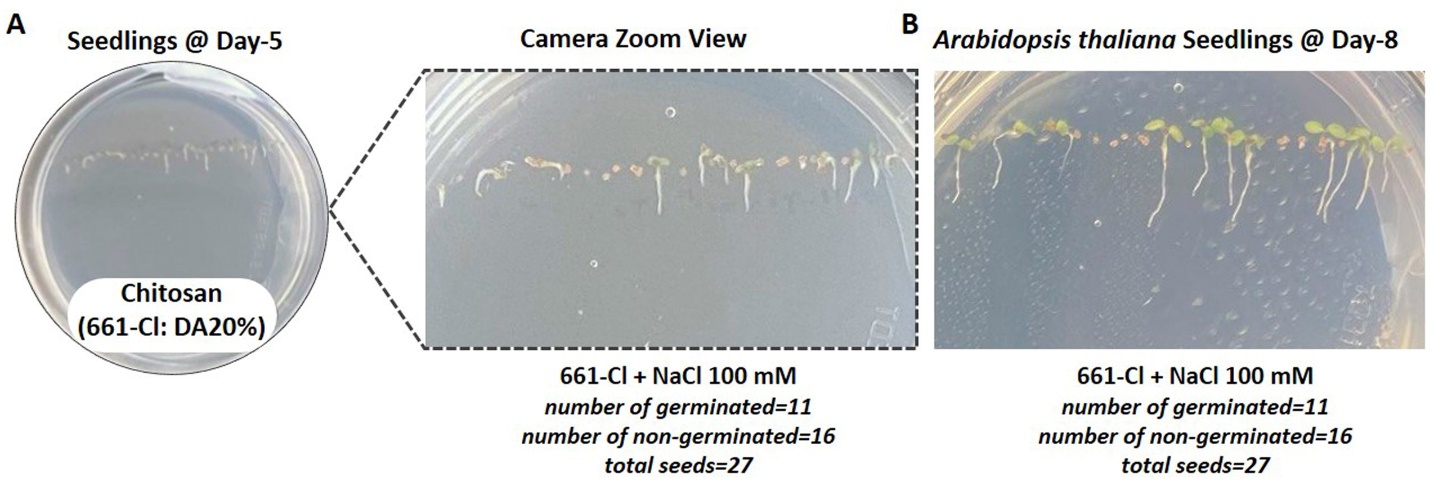


**Supplementary Figure S20: The bioactivity impact of a commercially available chitosan substance (661-Cl D_A_~20%) on enhancing seedlings tolerance to salt stress at different short- to mid-term time points.** **A**,**B**, The seeds of *Arabidopsis thaliana* Col-0 were line-cultured in solidified media with and without this chitosan solution at a similar concentration of 88 µg mL^−1^ and with 100 mM of NaCl solution. The camera images and their related seed germination analysis were compared with the bioactivity of optimal chiral MXene colloids on enhancing the seedlings’ maturation at day 5 and 8 post-treatment (n: is equal to at least twenty-five per dishes).

**Supplementary References:**

[S1] Suzuki, Nozomu, Yichun Wang, Paolo Elvati, Zhi-Bei Qu, Kyoungwon Kim, Shuang Jiang, Elizabeth Baumeister et al. "Chiral graphene quantum dots." *ACS nano* 10, no. 2 (2016): 1744-1755.

[S2] Cheng, Qingsong, Hebing Pei, Qian Ma, Ruibin Guo, Nijuan Liu, and Zunli Mo. "Chiral graphene materials for enantiomer separation." *Chemical Engineering Journal* 452 (2023): 139499.

[S3] Wang, Yichun. "Chiral Graphene Quantum Dot Enhanced Drug Loading into Exosomes." In *Electrochemical Society Meeting Abstracts 245*, no. 8, pp. 859-859. The Electrochemical Society, Inc., 2024.

[S4] Hall, Lyndon A., Deanna M. D’Alessandro, and Girish Lakhwani. "Chiral metal–organic frameworks for photonics." *Chemical Society Reviews* 52, no. 10 (2023): 3567-3590.

[S5] Zhang, Mengling, Yurong Ma, Huibo Wang, Bo Wang, Yunjie Zhou, Yang Liu, Mingwang Shao, Hui Huang, Fang Lu, and Zhenhui Kang. "Chiral control of carbon dots via surface modification for tuning the enzymatic activity of glucose oxidase." *ACS applied materials & interfaces* 13, no. 4 (2021): 5877-5886.

[S6] Zhao, Yuwan, Juan Xie, Yongzhi Tian, Stefanos Mourdikoudis, Nadesh Fiuza‐Maneiro, Yanli Du, Lakshminarayana Polavarapu, and Guangchao Zheng. "Colloidal chiral carbon dots: an emerging system for chiroptical applications." *Advanced Science* 11, no. 13 (2024): 2305797.

[S7] Ostadhossein, Fatemeh, Gururaja Vulugundam, Santosh K. Misra, Indrajit Srivastava, and Dipanjan Pan. "Chirality inversion on the carbon dot surface via covalent surface conjugation of cyclic α-amino acid capping agents." *Bioconjugate Chemistry* 29, no. 11 (2018): 3913-3922.

[S8] Li, Jinqiu, Ning Du, Ruifang Guan, and Songfang Zhao. "Construction of a chiral fluorescent probe for tryptophan enantiomers/ascorbic acid identification." *ACS Applied Materials & Interfaces* 15, no. 19 (2023): 23642-23652.

[S9] Vázquez-Nakagawa, Mikiko, Laura Rodríguez-Pérez, M. Á Herranz, and Nazario Martín. "Chirality transfer from graphene quantum dots." *Chemical Communications* 52, no. 4 (2016): 665-668.

[S10] Li, Feng, Yiye Li, Xiao Yang, Xuexiang Han, Yang Jiao, Taotao Wei, Dayong Yang, Huaping Xu, and Guangjun Nie. "Highly fluorescent chiral N‐S‐doped carbon dots from cysteine: affecting cellular energy metabolism." *Angewandte Chemie* 130, no. 9 (2018): 2401-2406.

[S11] Arshad, Farwa, and Md Palashuddin Sk. "Aggregation-induced red shift in N, S-doped chiral carbon dot emissions for moisture sensing." *New Journal of Chemistry* 43, no. 33 (2019): 13240-13248.

[S12] Yang, Yongzhen, Qin Wang, Gongjian Li, Wenjing Guo, Zuojun Yang, Hao Liu, and Xiaoyuan Deng. "Cysteine-derived chiral Carbon Quantum dots: a fibrinolytic activity Regulator for Plasmin to target the human islet amyloid polypeptide for type 2 diabetes Mellitus." *ACS Applied Materials & Interfaces* 15, no. 2 (2023): 2617-2629.

[S13] Zhang, Mengling, Huibo Wang, Bo Wang, Yurong Ma, Hui Huang, Yang Liu, Mingwang Shao, Bowen Yao, and Zhenhui Kang. "Maltase decorated by chiral carbon dots with inhibited enzyme activity for glucose level control." *Small* 15, no. 48 (2019): 1901512.

[S14] Hu, Lulu, Hao Li, Yuxiang Song, Mengling Zhang, Hui Huang, Yang Liu, and Zhenhui Kang. "Chiral evolution of carbon dots and the tuning of laccase activity." *Nanoscale* 10, no. 5 (2018): 2333-2340.

[S15] Zhang, Mengling, Xing Fan, Xin Du, Yurong Ma, Xiting Wang, Hui Huang, Yang Liu, Youyong Li, and Zhenhui Kang. "Chiral carbon dots from glucose by room temperature alkali-assisted synthesis for electrocatalytic oxidation of tryptophan enantiomers." *Nano Research* 16, no. 7 (2023): 8929-8936.

[S16] Xin, Qi, Qian Liu, Lingling Geng, Qiaojun Fang, and Jian Ru Gong. "Chiral nanoparticle as a new efficient antimicrobial nanoagent." *Advanced healthcare materials* 6, no. 4 (2017): 1601011.

[S17] Zhang, Mengling, Lulu Hu, Huibo Wang, Yuxiang Song, Yang Liu, Hao Li, Mingwang Shao, Hui Huang, and Zhenhui Kang. "One-step hydrothermal synthesis of chiral carbon dots and their effects on mung bean plant growth." *Nanoscale* 10, no. 26 (2018): 12734-12742.

[S18] Gao, Pengli, Zhigang Xie, and Min Zheng. "Chiral carbon dots-based nanosensors for Sn (II) detection and lysine enantiomers recognition." *Sensors and Actuators B: Chemical* 319 (2020): 128265.

[S19] Li, Rong Sheng, Peng Fei Gao, Hong Zhi Zhang, Lin Ling Zheng, Chun Mei Li, Jian Wang, Yuan Fang Li, Feng Liu, Na Li, and Cheng Zhi Huang. "Chiral nanoprobes for targeting and long-term imaging of the Golgi apparatus." *Chemical science* 8, no. 10 (2017): 6829-6835.

[S20] Gao, Pengli, Shuang Chen, Shi Liu, Hongxin Liu, Zhigang Xie, and Min Zheng. "Chiral carbon dots-enzyme nanoreactors with enhanced catalytic activity for cancer therapy." *ACS Applied Materials & Interfaces* 13, no. 47 (2021): 56456-56464.

[S21] Arad, Elad, Susanta Kumar Bhunia, Jürgen Jopp, Sofiya Kolusheva, Hanna Rapaport, and Raz Jelinek. "Lysine‐derived carbon dots for chiral inhibition of prion peptide fibril assembly." *Advanced Therapeutics* 1, no. 4 (2018): 1800006.

[S22] Malishev, Ravit, Elad Arad, Susanta Kumar Bhunia, Shira Shaham-Niv, Sofiya Kolusheva, Ehud Gazit, and Raz Jelinek. "Chiral modulation of amyloid beta fibrillation and cytotoxicity by enantiomeric carbon dots." *Chemical Communications* 54, no. 56 (2018): 7762-7765.

[S23] Hallaji, Zahra, Zeinab Bagheri, and Bijan Ranjbar. "One-step solvothermal synthesis of red chiral carbon dots for multioptical detection of water in organic solvents." *ACS Applied Nano Materials* 6, no. 5 (2023): 3202-3210.

[24] Liao, Xuan, Bingyan Wu, Haixia Li, Mengtao Zhang, Muzi Cai, Bozhi Lang, Zhizhen Wu et al. "Fluorescent/colorimetric dual-mode discriminating Gln and Val enantiomers based on carbon dots." *Analytical chemistry* 95, no. 39 (2023): 14573-14581.

[S25] Chekini, Mahshid, Elisabeth Prince, Lily Zhao, Haridas Mundoor, Ivan I. Smalyukh, and Eugenia Kumacheva. "Chiral carbon dots synthesized on cellulose nanocrystals." *Advanced Optical Materials* 8, no. 4 (2020): 1901911.

[S26] Ma, Shuo, Huanyu Ma, Kai Yang, Zhan’ao Tan, Biao Zhao, and Jianping Deng. "Intense circularly polarized fluorescence and room-temperature phosphorescence in carbon dots/chiral helical polymer composite films." *ACS nano* 17, no. 7 (2023): 6912-6921.

[S27] Basu, Srestha, and Nadav Amdursky. "The Role of Surface Groups in Dictating the Chiral‐Solvent‐Induced Assembly of Carbon Dots into Structures Exhibiting Circularly Polarized Luminescence." *Small* 19, no. 8 (2023): 2205880.

[S28] Jiang, Kangde, Qingyan Fan, Dekang Guo, Chunfeng Song, and Jinbao Guo. "Circularly polarized room-temperature phosphorescence with an ultrahigh dissymmetry factor from carbonized polymer dots by stacked chiral photonic films." *ACS Applied Materials & Interfaces* 15, no. 21 (2023): 26037-26046.

[S29] Niu, Xiaohui, Rui Zhao, Simeng Yan, Zengwei Pang, Hongxia Li, Xing Yang, and Kunjie Wang. "Chiral materials: progress, applications, and prospects." *Small* 19, no. 38 (2023): 2303059.

[S30] Liu, Junjun, Lin Yang, Ping Qin, Shiqing Zhang, Ken Kin Lam Yung, and Zhifeng Huang. "Recent advances in inorganic chiral nanomaterials." *Advanced Materials* 33, no. 50 (2021): 2005506.
